# Dopant-Directed Surface Energy Modulation for Controlled Growth and Color-Tunable Photoluminescence in Cu^+^‑Doped Two-Dimensional CdSe Nanoplatelets

**DOI:** 10.1021/acsnano.6c08297

**Published:** 2026-08-05

**Authors:** Vanshika Vanshika., Sara Talebi, Joshua T. Wright, Poojan Kaswekar, Anthony Moses, Yuchen Zhang, Emily Qiao, Robert W. Meulenberg, Arindam Chakraborty, Weiwei Zheng

**Affiliations:** † Department of Chemistry, 2029 Syracuse University, Syracuse, New York 13244, New York, United States; ‡ Department of Physics, 2455 Illinois Institute of Technology, Chicago 60616, Illinois, United States; § Department of Mechanical and Aerospace Engineering, Syracuse University, Syracuse 13244, New York, United States; ∥ Department of Physics and Astronomy and Frontier Institute for Research in Sensor Technologies, 6250 University of Maine, Orono 04469, Maine, United States

**Keywords:** 2D nanoplatelets, Cu^+^ doping, thickness-controlled growth, surface energy modulation, optical properties

## Abstract

Two-dimensional (2D) semiconductor nanoplatelets (NPLs) are promising building blocks for optoelectronic applications due to their strong 1D quantum confinement and large lateral dimensions. While the growth mechanisms of pristine CdSe NPLs have been extensively studied, the influence of transition-metal dopants on 2D NPL growth remains poorly understood. Here, we investigate the growth behavior of Cu^+^-doped CdSe NPLs synthesized via a nucleation-doping colloidal approach. The introduction of Cu^+^ induces a dopant-modulated three-stage growth process. At 230 °C, Cu^+^ dopants initially decelerate NPL growth, stabilizing 2-monolayer (ML) CdSe NPLs in stage I. In stage II, these 2 ML NPLs undergo a dissolution–ripening process, ultimately leading to the formation of 3 ML NPLs in stage III. The resulting Cu-doped CdSe NPLs exhibit color-tunable photoluminescence (PL) arising from both the CdSe host excitonic emission (∼390–465 nm) and a broad, Stokes-shifted Cu^+^ dopant emission (∼520–640 nm), enabled by thickness-dependent bandgap modulation. This dopant-directed three-stage growth behavior is also observed in thicker Cu:CdSe NPLs (*e.g.,* 4 ML). Theoretical simulations reveal that Cu^+^ doping increases the relative surface stabilization energy, stabilizing thinner NPLs and delaying the transition to thicker structures. This dopant-driven control over 2D NPL growth offers opportunities for engineering color-tunable and dopant-emissive optoelectronic materials.

## Graphical abstract

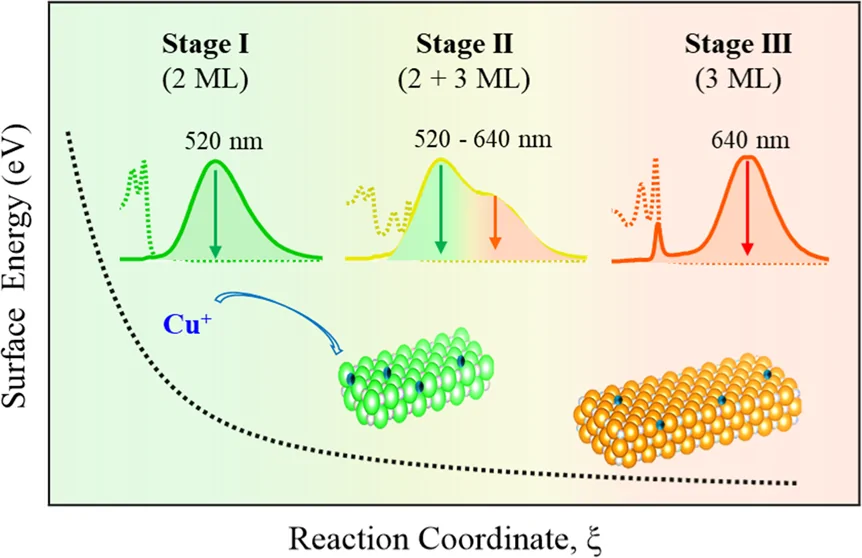

## Introduction

Two-dimensional (2D) semiconductor nanoplatelets (NPLs) with quantum confinement along their thickness (<10 nm)^1–3^ are of significant interest owing to their unique electronic structure, including extremely narrow photoluminescence (PL),^4,5^ high PL quantum yields (PL QY ∼ 80%),^6,7^ low lasing threshold,^8^ giant oscillator strength transition,^9^ fast Förster resonant energy transfer,^10^ and highly polarized emission.^8,11^ Thus, 2D NPLs are promising materials for applications in solar cell concentrators,^7^ light-emitting diodes (LEDs),^12^ lasers,^13^ and field-effect transistors.^10^ High-quality 2D anisotropic NPLs, such as CdS,^4,14^ CdSe,^4,15^ CdTe,^4^ ZnSe,^16^ and PbS^17^ with thicknesses of a few monolayers (MLs) and lateral dimensions between 20 nm and a few hundred nanometers have been synthesized using colloidal synthetic methods at temperatures between 140 °C and 250 °C.

Considerable effort has been devoted to understanding the anisotropic growth and thickness control of CdSe NPLs, which crystallize in the zinc blende lattice. Ithurria *et al.* demonstrated that CdSe NPL thickness can be tuned from 2 to 5 ML by varying the reaction temperature from 170 °C (thin NPLs) to 250 °C (thick NPLs).^4^ Thicker CdSe NPLs, ranging from 5 to 8 ML, could be obtained at higher temperatures (240–320 °C) using additives such as CdCl_2_.^18^ In addition to the temperature-controlled growth of 2D CdSe NPLs, post-synthetic thermal annealing has been shown to induce thickness evolution. For example, 2 ML CdSe NPLs annealed at 180 °C, in the presence of pentanoic acid (C_5_H_11_COOH) to form in situ reactive cadmium pentanoate, gradually transformed into 3 ML NPLs after 5 h and into 4 ML NPLs after 3 days.^19^ Also, using cadmium propionate (Cd­(C_2_H_5_COO)_2_) and Se powders at 200 °C, slow ripening of 2 ML CdSe NPLs to form thicker 3 ML NPLs was observed over the course of 3–18 h.^19,20^

Early models proposed that 2D NPLs form within amine bilayer templates via metal salt dissolution, yielding lamellar structures; however, the precise role of such templates remains unclear.^21,22^ Later, Riedinger *et al.* proposed a 2D nucleation and growth model for anisotropic CdSe NPL growth in an isotropic medium.^23^ In this model, the energy of the system (Δ*E*) is calculated at two facets (narrow and wide) of the Cd-terminated zincblende CdSe crystallite using density functional theory. This model describes the nucleation of an additional ML on the facet with a low energy barrier (*i.e.*, narrow facets along edges) via symmetry breaking, which is a thermodynamically driven process. The 2D nucleation process has a distinct nucleation barrier along different growing crystal facets, leading to fast growth on a low nucleation barrier narrow facet (001) compared to a wide facet (110). The driving force to increase the thickness of NPLs is the reduction of the total surface area of relatively less stable thin NPLs, which can be observed during the prolonged thermal treatment and 2D Ostwald ripening.^19^

To date, a few doped 2D II–VI metal chalcogenide NPLs, such as Mn-doped ZnSe^24^ and CdS,^25^ Ag-doped CdSe,^26^ and Cu-doped CdSe^27^ have been reported, with most studies focusing on dopant-related PL properties. The doping of transition metal ions (*e.g.,* Mn^2+^, Cu^+^, and Ag^+^) into semiconductor nanocrystals introduces localized dopant energy levels within the bandgap of the host lattice,^28–30^ enabling new pathways of radiative relaxation in NCs and improving their optical and electronic properties.^28–32^ For instance, Wang *et al.* have recently investigated the excited-state dynamics and light-emission mechanisms of Mn-doped zinc blende CdSe NPLs and demonstrated that the atomically thin NPLs promote strong host-dopant coupling, leading to broadband charge-transfer absorption and emission (∼575 nm) and the formation of an excited-state species (likely Mn–Mn dimer) with a characteristic near-infrared emission.^33^ In contrast, Cu-doped II–VI chalcogenide NCs exhibit strong Cu^+^-related emission arising from deep acceptor states that act as radiative recombination centers. This process involves charge recombination between the host conduction band and Cu^+^ midgap states, commonly described as metal-to-ligand charge transfer (ML_CB_CT).^34–37^ Sharma *et al.* synthesized Cu-doped CdSe NPLs (*i.e.*, Cu:CdSe) by hot injection of a Se precursor into Cd myristate and Cd acetate reaction mixture with a Cu precursor at 220 °C–250 °C.^6,7^ These Cu:CdSe NPLs display high PL QYs (80–90%), large absorption cross-section, and strongly Stokes-shifted emission.^6,7,27^ Despite these advances, the influence of dopants on the growth kinetics and thickness evolution of 2D NPLs remains largely unexplored.

In this work, we systematically studied the role of Cu^+^ dopants in the growth behavior of the 2D CdSe NPLs. Under the same reaction conditions for the direct growth of 3 ML CdSe NPLs, incorporation of Cu^+^ surprisingly induces a thickness evolution from 2 to 3 ML. This dopant-mediated growth proceeds through a distinct three-stage process: (i) formation of thin 2 ML NPLs, (ii) gradual transformation of 2 ML into 3 ML NPLs, and (iii) growth of larger 3 ML NPLs, as evidenced by thickness-dependent absorption and dual PL from both the CdSe host and Cu^+^ dopants (Scheme 1). The Cu^+^ dopant-modulated successive growth process can also be expanded to thicker Cu:CdSe NPLs (*e.g.*, 4 ML NPLs) at higher-temperature growth conditions. Stochastic growth simulations reveal that Cu^+^ doping leads to a higher relative surface stabilization energy of thinner NPLs, stabilizing 2 ML structures and delaying the transition to thicker NPLs compared to pristine CdSe NPLs. Notably, during the successive growth of Cu:CdSe NPLs, the number of Cu^+^ dopants per NPL remains constant due to nucleation doping, whereas the effective doping concentration decreases as the CdSe NPLs grow thicker and larger over time. Consequently, at the later growth stages, the reduced Cu^+^ content renders the surface energy of light Cu-doped CdSe NPLs comparable to that of pristine CdSe NPLs, favoring the formation of thicker NPLs. This interpretation is further supported by X-ray absorption fine structure (XAFS) analysis, which indicates that Cu^+^ ions are initially located on or near the surface and exhibit reduced coordination numbers, which are consistent with Se vacancy formation. As NPL growth proceeds at later stages, the reduced Cu^+^ dopant concentration leads to coordination numbers approaching those of pristine CdSe. These findings underscore the fundamental role of dopant-directed growth in 2D NPLs and open opportunities for tailoring their optical and electronic properties for advanced optoelectronic applications.

**Scheme 1:**
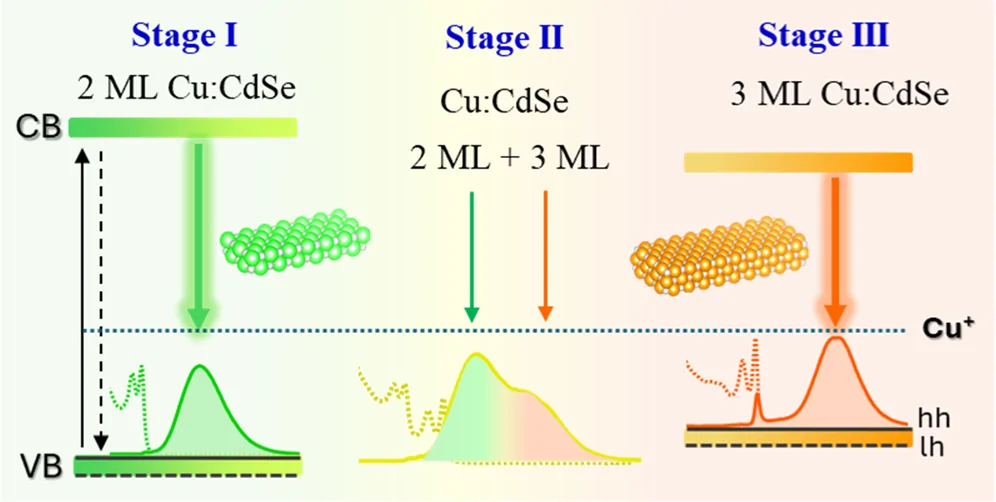
Schematic Illustration of Cu:CdSe NPLs *via* a 3-Stage Process. (I) Formation of 2 ML NPLs with Cu^+^ Dopant PL at 520 nm, (II) Appearance of 2 and 3 ML NPLs with Two Cu^+^ Dopant PL at 520 and 640 nm, and (III) Formation of 3 ML NPLs with Cu^+^ Dopant PL at 640 nm.

## Results

In this study, we synthesized Cu^+^- doped CdSe 2D NPLs (*i.e.*, Cu:CdSe) by nucleation doping of Cu^+^ ions in CdSe NPLs by the hot injection of a Se precursor into Cd acetate at 230 °C (see details in the Experimental Section). Figure 1a shows the normalized absorption and PL spectra of Cu:CdSe NPLs at different reaction times. UV–visible absorption of Cu:CdSe NPLs exhibits both electron light-hole (e-lh) and electron heavy-hole (e-hh) transitions at 372 and 393 nm from 1 to 4 min of reaction, which corresponds to 2 ML CdSe NPLs (Note: only compared e-hh transitions for different CdSe NPLs for simplification in the following discussion). A strong Cu^+^ PL peak has been seen at 520 nm because of charge transfer between the CdSe host and Cu^+^ dopant (*i.e.*, ML_CB_CT) with 100–120 nm full width at half maximum (fwhm). In addition, a very weak host bandgap PL around 395 nm was observed for Cu:CdSe NPLs grown at 4 and 5 min of reaction. The large Stokes-shifted PL in Cu:CdSe NPLs is because of the coupling between the excitons at Cu^+^ midgap states and the conduction band of CdSe NPLs.^6,7,34^ Then, two groups of absorption peaks at ∼390 nm and ∼460 nm appear for ∼5–6 min of reaction time, with two Cu^+^ PL peaks at 520 and 640 nm with a much broader apparent fwhm of 124–135 nm. After 7 min of growth, the 460 nm absorption peak from 3 ML CdSe NPLs becomes the dominant peak, and the Cu^+^ PL peak redshifts to 640 nm along with bandgap PL at 465 nm, and the fwhm of Cu^+^ PL decreased to 100–93 nm (Figure 1b–d).

**Figure 1:**
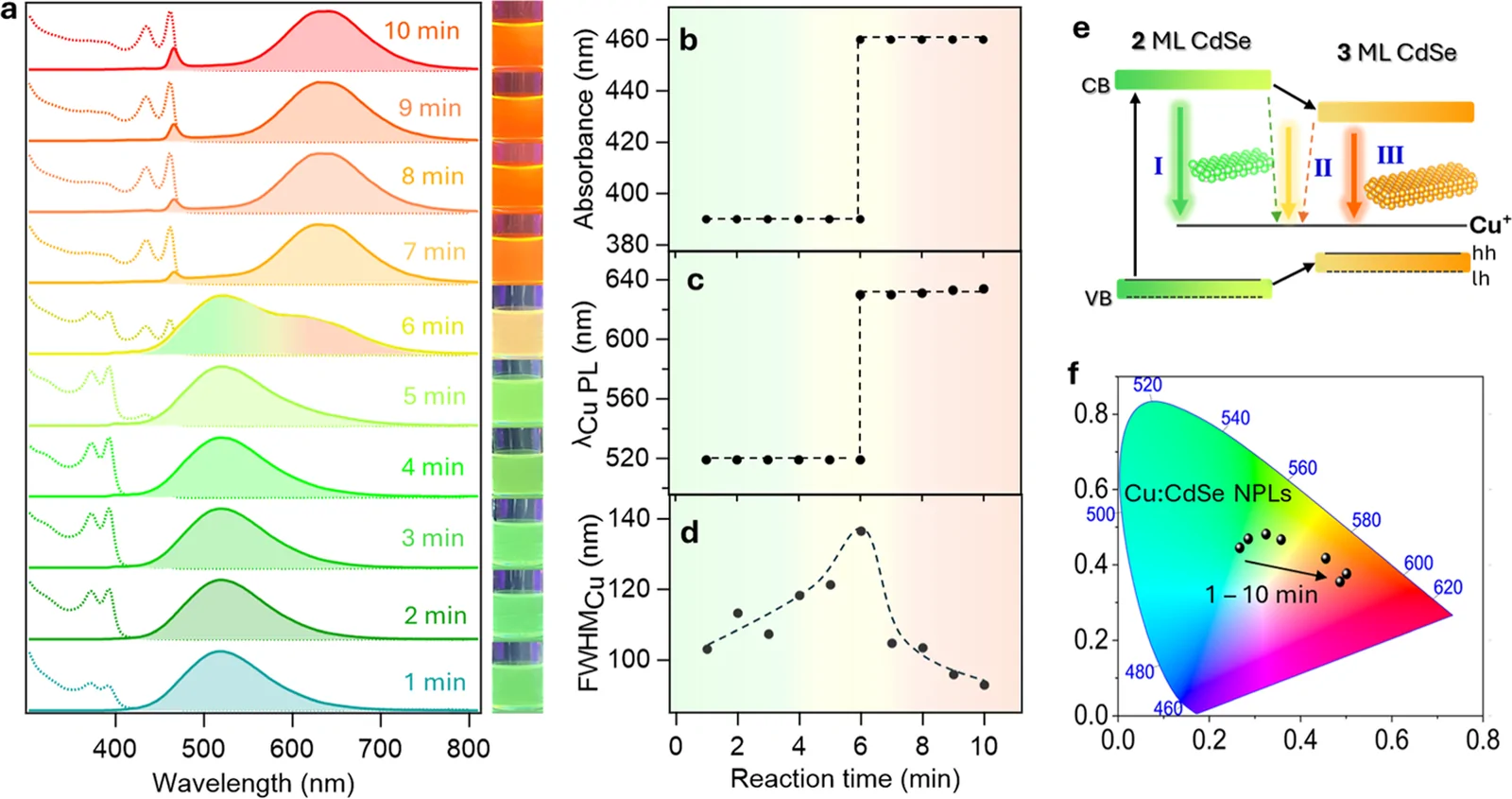
(a) Normalized absorption (dotted lines) and PL (solid lines) spectra of Cu:CdSe NPLs with optical images on the right side. (b) Host absorbance, (c) Cu^+^ dopant PL peak, and (d) and fwhm of Cu^+^ dopant PL in Cu:CdSe NPLs. (e) Schematic illustration of the thickness-dependent Cu^+^ dopant PL during three growth stages of Cu:CdSe NPLs. (f) Chromaticity coordinates of Cu:CdSe NPLs.

It is intriguing to observe the successive growth of 2D Cu:CdSe NPLs with thickness-dependent absorption and PL, which include three stages: (I) the formation of 2 ML Cu:CdSe NPLs with absorption at ∼390 nm and Cu^+^ PL centered at 520 nm, (II) mixture of 2 and 3 ML Cu:CdSe NPLs with two groups of absorption peaks at ∼390 nm and ∼460 nm with two Cu^+^ PL peaks at 520 and 640 nm, and (III) finally, 3 ML NPLs were achieved with red-shifted absorption at ∼460 nm and Cu^+^ PL at 640 nm. In a control experiment, undoped CdSe NPLs were synthesized under the same experimental conditions from 1 to 10 min. Figure S1a–c displays almost unchanged absorption at ∼460 nm and a narrow bandgap PL at 465 nm with nearly the same fwhm (∼10 nm) from 3 ML CdSe NPLs and a broad, weak trap emission similar to earlier studies for 3 ML CdSe NPLs.^7,38,39^ Both the red-shifted Cu^+^ PL and the step-like change in absorbance (∼70 nm) in Cu/CdSe NPLs (Figure 1e), compared to undoped CdSe NPLs, which show no such absorbance step between 1 and 10 min of reaction, indicate a thickness evolution from 2 to 3 ML CdSe NPLs induced by the presence of Cu^+^ ions.

The PL QY of Cu:CdSe NPLs increases from ∼20% to ∼ 80% during the thickness evolution from 2 to 3 MLs (Figure S2). Notably, the PL QY of pristine 3 ML CdSe NPLs remains relatively low (∼40%, Figure S1d) compared to that of Cu:CdSe NPLs.^6^ Therefore, the significantly enhanced PL QY in Cu:CdSe NPLs could be attributed to efficient charge transfer between the CdSe host and Cu^+^ dopants (i.e., ML_CB_CT), as well as reduced surface trap densities that promote radiative recombination in thicker 3 ML NPLs relative to 2 ML NPLs. With the transition from 2 to 3 ML Cu:CdSe NPLs, the emission color of the NPLs was continuously tuned from bright green to yellowish green and finally stabilized with an orange light emission (Figure 1f), unlike nearly constant orange color in 3 ML undoped CdSe NPLs (Figure S1e), as indicated by Commission International de l’Eclariage (CIE) coordinates and optical pictures under UV light irradiation (right panel of Figures 1a and S1a).

Transmission electron microscopy (TEM) images of the as-prepared Cu:CdSe NPLs indicate 2D NPLs with an average length of ∼70 ± 15 nm for 2 ML NPLs (Stage I, Figure 2a,b). At Stage II (Figure 2c,d), the NPLs consist of coexisting 2 and 3 ML populations with an increased average length of ∼85 ± 25 nm, which further increases to ∼140 ± 15 nm upon formation of 3 ML NPLs (Stage III, Figure 2e,f). However, the undoped CdSe NPLs maintain a dimension of approximately 62 ± 15 nm for 3 ML NPLs throughout the synthesis at the same reaction conditions (Figure S3). This contrasting growth behavior between doped and undoped CdSe NPLs might be because of the Cu^+^ dopant ions and/or vacancies, which initially stabilize the surface of NPLs, leading to the formation of small 2 ML NPLs. During the transition from 2 to 3 ML NPLs, the growth of NPLs in all three directions results in longer and wider NPLs. The reaction time-dependent TEM images from 1 to 10 min are displayed in Figure S4. The high-resolution TEM (HR-TEM) image gives a *d*-spacing of ∼0.25 nm from the (220) plane in Cu:CdSe NPLs at three different stages, which suggests that 3 ML NPLs grow from 2 ML NPLs without affecting the original crystal structure (Figure S5). Furthermore, because ultrathin NPLs tend to roll up,^15,40^ we passivated the surface of Cu:CdSe NPLs with a CdS shell to increase their rigidity and induce unrolling of the 2D NPLs (Figure S6). After surface passivation, Cu:CdSe/CdS nanosheets were obtained with lengths comparable to those of the uncoated Cu:CdSe NPLs; however, their widths increased by approximately 3–3.5 times across all three growth stages.

**Figure 2:**
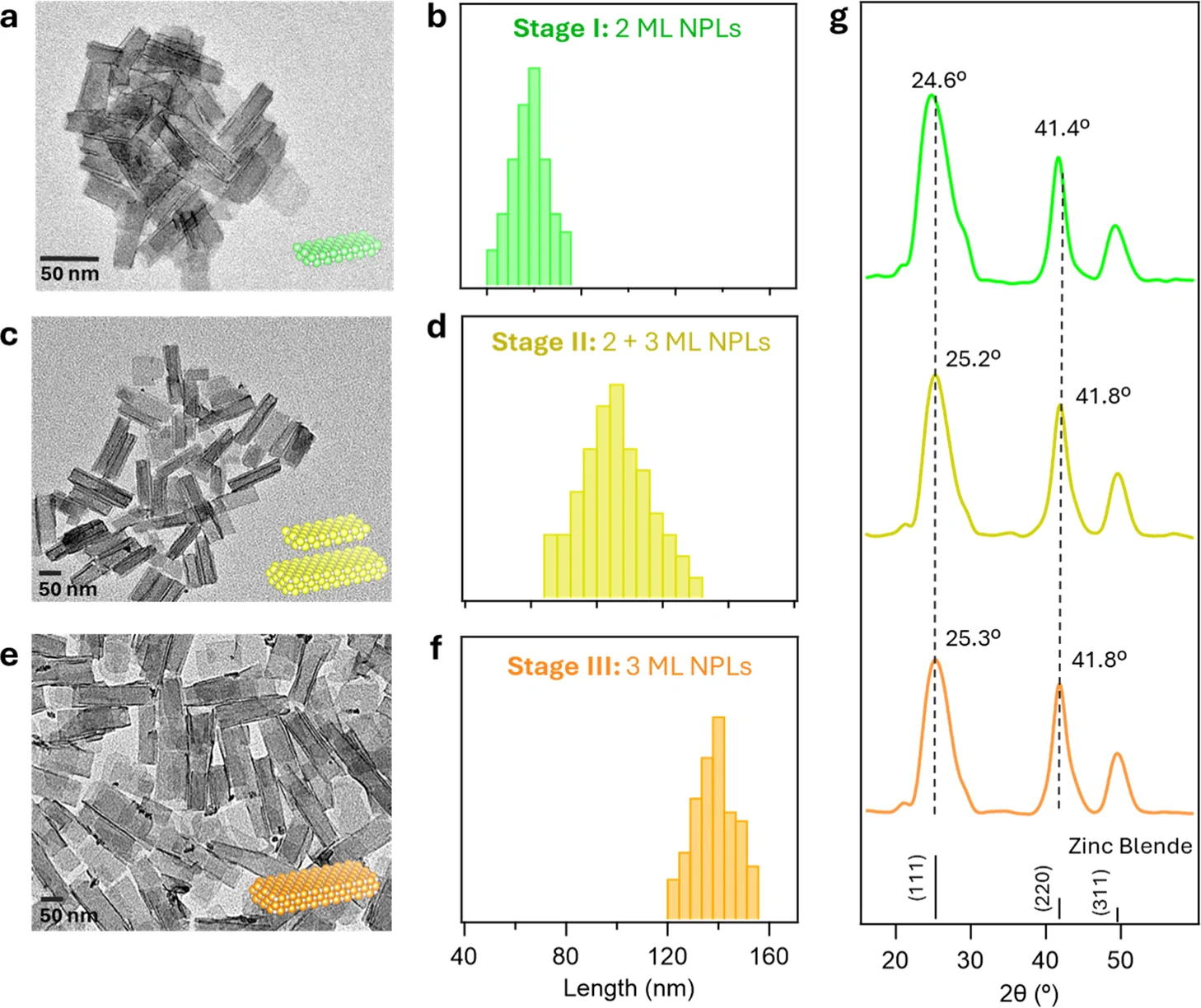
TEM images and size histograms of (a,b) Stage I: 2 ML, (c,d) Stage II: 2 + 3 ML, and (e,f) Stage III: 3 ML Cu:CdSe NPLs. (g) X-ray diffraction (XRD) pattern at three growth stages of Cu:CdSe NPLs.

Powder X-ray diffraction (XRD) measurements reveal that the Cu:CdSe NPLs (Figure 2g) and undoped CdSe NPLs (Figure S7) possess a zinc blende structure of CdSe. For 2 ML NPLs (stage I), the (111) and (220) diffraction peaks shift to lower angles (24.6° and 41.4°, respectively) relative to bulk cubic CdSe and exhibit noticeable peak broadening, as reflected by the increased fwhm (Figure S8). These features indicate that ultrathin 2 ML Cu:CdSe NPLs experience both uniform and nonuniform lattice distortions, consistent with previous reports.^41^ Upon the formation of thicker 3 ML NPLs at stages II and III, the (111) peak becomes more symmetric with decreased fwhm (Figure S8b,c) and shifts to a higher angle (25.3°), approaching the values of bulk zinc blende CdSe and reported 3 ML CdSe NPLs (Figures 2g and S8).^2,18^

Additionally, the (220) diffraction is observed to split into a sharp (220) peak and a broader (202) component, a phenomenon attributed to nonuniform lattice deformation in NPLs.^42,43^ These diffractions arise from crystallographic planes belonging to the same family but oriented along the lateral [(220), sharp] and thickness [(202), broad] directions of the NPLs (Figure S9). The lattice parameters along the lateral and thickness directions were calculated using eq 1.^42^

1 $$\frac{1}{d^{2}}=\left(\frac{h^{2}+k^{2}}{a^{2}}\right)+\left(\frac{l^{2}}{c^{2}}\right)$$

where *a* and *c* denote the lattice parameters along the lateral and thickness directions, respectively, and *d* is the interplanar spacing. The calculated lateral lattice parameters for 2 and 3 ML Cu:CdSe NPLs (6.356 Å and 6.212 Å, respectively; Supporting Information Section A) are larger than that of bulk cubic CdSe (6.05 Å), whereas the lattice parameters along the thickness direction are smaller (5.986 Å and 5.947 Å for 2 and 3 ML, respectively). These results indicate lattice contraction along the thickness direction accompanied by lateral lattice expansion, in good agreement with previous observations by Peng *et al*.^2^

Inductively coupled plasma-optical emission spectrometry (ICP-OES) measurements indicate that the doping concentration (calculated from the ratio of Cu^+^/Cd^2+^ ions) significantly decreased from 0.6% to 0.1% as a function of reaction time (Figure S10a). Interestingly, despite continuous NPL growth with increasing reaction time, the calculated number of Cu^+^ dopant ions per NPL remains nearly constant within experimental error (∼263 ± 28 for 2 ML, 243 ± 42 for mixed 2 + 3 ML, and 239 ± 20 for 3 ML NPLs) throughout the growth of NPLs with ∼ 4-fold increasing volume from smaller 2 ML to larger 3 ML NPLs as determined from TEM analysis (Figure S10b). The same number of Cu^+^ dopants for both 2 and 3 ML CdSe NPLs supports the nucleation doping in our synthesis.

## Discussion

### Exciton Relaxation and Charge Transfer Behavior

Time-resolved PL measurements of the Cu:CdSe NPLs were performed to study the exciton relaxation and charge transfer behavior (Figure 3a). The τ_av_ of Cu^+^ dopants PL significantly increases from ∼90 ns to ∼ 270 ns from 2 ML (1–6 min @ 520 nm) to 3 ML (7–10 min @ 635 nm) Cu:CdSe NPLs, which is in agreement with previously reported Cu:CdSe NPLs.^18,44^ The growth of thicker NPLs leads to large band-edge oscillator strength and exciton coupling, as shown by the red-shifted CdSe and Cu^+^ PL peak in Figure 1a which amplifies τ_av_. During the transition from 2 to 3 ML NPLs in Stage II (5–7 min), two Cu^+^ PL peaks around 520 and 635 nm have been observed with two lifetime decay (τ_av_ ∼ 90 ns and ∼270 ns, respectively, Figure S11).

**Figure 3:**
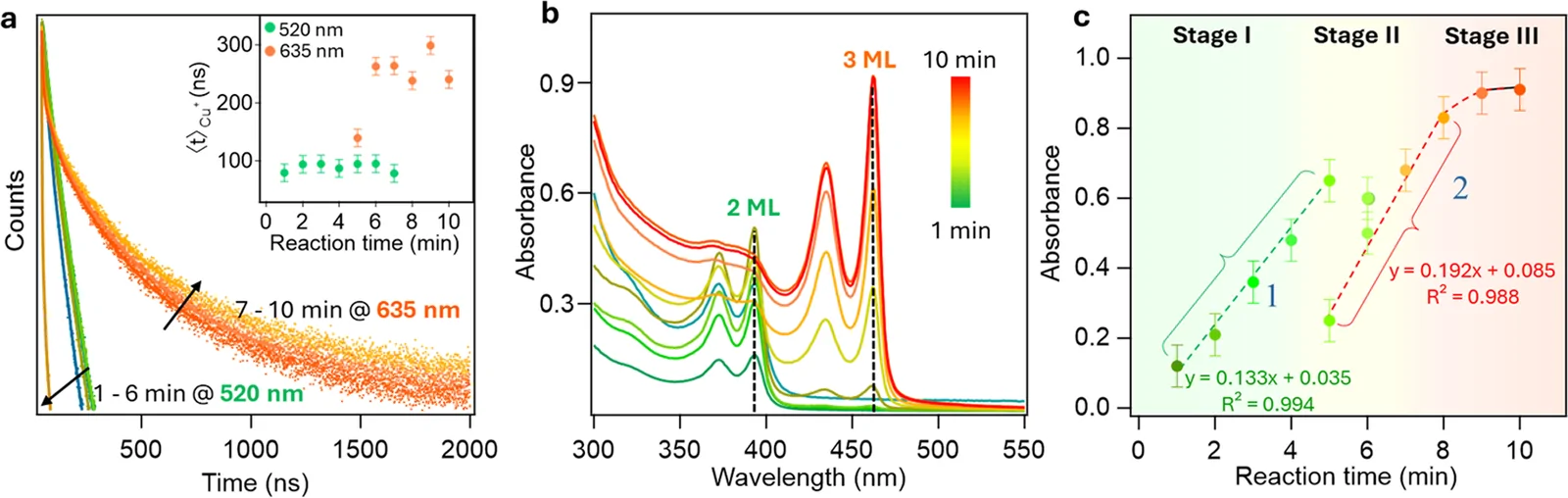
(a) PL decay of Cu^+^ dopants of Cu:CdSe NPLs as a function of reaction time. (b) Time-dependent absorbance at 390 and 465 nm is fitted by (c) a linear relationship for 2 and 3 ML Cu:CdSe NPLs.

Furthermore, the growth of 3 ML NPLs from 2 ML NPLs could be confirmed by the varied contribution of decay parameters of Cu^+^ PL at the three growth stages for Cu:CdSe NPLs (Table S1). The PL decay of Cu:CdSe NPLs, I­(t), is fitted to a three-exponential decay model

2 $$I\left(t\right)=B_{1}e^{‐t/\tau 1}+B_{2}e^{‐t/\tau 2}+B_{3}{}_{e}^{‐t/\tau 3}$$

where B_1_, B_2_, and B_3_ are pre-exponential factors for the corresponding decay time constants τ_1_, τ_2_, and τ_3_. Initially, Cu^+^ dopant PL from the 2 ML NPLs obtained in Stage I has an average contribution of 77% from τ_2_. For 3 ML NPLs obtained in Stage III, 90% of the average contribution of lifetime is from the τ_3_ decay component. In the transition phase (Stage II), both 2 and 3 ML NPLs are present, and τ_2_ and τ_3_ have intermediate contributing decay constants (40% and 58%, respectively) between those of Stage I and III, which suggests significant radiative decay from both 2 and 3 ML NPL centers at Stage II. The varied contribution from different decay parameters signifies the tunable host-to-dopant charge transfer resulting from the different radiative centers and PL QYs.

To gain further insights into the growth process of Cu:CdSe NPLs, we studied time-dependent absorbance of Cu:CdSe NPLs by taking 20 μL of aliquots at each reaction time and dissolved in 1 mL of toluene for absorption spectroscopy (Figure 3b). We observed the absorbance peak shift from ∼390 nm (2 ML NPLs) to ∼ 460 nm (3 ML NPLs) with increasing reaction time. More importantly, the intensity of each absorption peak changes with reaction time and can be fitted to a linear relationship with a slope of 0.133 and 0.192 corresponding to the growth of 2 and 3 ML NPLs, respectively (Figure 3c), which might indicate a slower growth of 3 ML Cu:CdSe NPLs at the early stage (Stage I) and slightly faster at the later stage (Stage III) for 3 ML Cu:CdSe.^45^ The absorption intensity ∼460 nm from 3 ML NPLs remains nearly constant after ∼9 min of reaction, indicating depletion of the reactants and completion of 3 ML NPL formation. In contrast, undoped CdSe NPLs (Figure S12) exhibit a linear increase in absorbance at 465 nm as a function of reaction time, consistent with the continuous growth of 3 ML CdSe NPLs.

### Impact of Cu^+^ Dopants in the Growth of CdSe NPLs

The growth behavior of CdSe NPLs is impacted by the incorporation of Cu^+^ dopant ions and shows interesting 3-stage 2D NPL growth. The Cu^+^ dopant ions are incorporated during the nucleation stage of NPL formation, with the number of Cu^+^ dopant ions per NPL nearly constant; therefore, the Cu^+^ doping concentration keeps decreasing with time throughout the expansion of 2 to 3 ML NPLs (Figure S9). On the basis of the nucleation theory for the growth of 2D NPLs, islands of precursor nucleate on the facet(s) with low nucleation barriers, and it has a thermodynamic driving force to form an ML by expanding onto the facet(s).^5,16,46^ The observed thickness evolution of NPLs (2 to 3 ML) could be explained by dissolution-ripening of laterally smaller 2 ML NPLs and discrete growth of thick 3 ML NPLs, which is referred to as 2D Ostwald ripening for 2D nanomaterials.^20^

To understand the role of Cu^+^ dopants in the growth of Cu:CdSe NPLs, we have synthesized Cu:CdSe NPLs with different Cu^+^ dopant concentrations by adding more Cu acetate precursors (2% and 3% in synthesis) under the same reaction conditions (Figure 4a,b). ICP-OES analysis indicates a high Cu^+^ dopant concentration, where the initial Cu^+^/Cd^2+^ ratio is 1.1% (for 2% Cu) and 1.5% (for 3% Cu), which gradually decreases as the reaction progresses (Figure S13). XRD reveals that the zinc blende crystal structure remains intact with increasing Cu^+^ dopant concentration (Figure S14).

**Figure 4:**
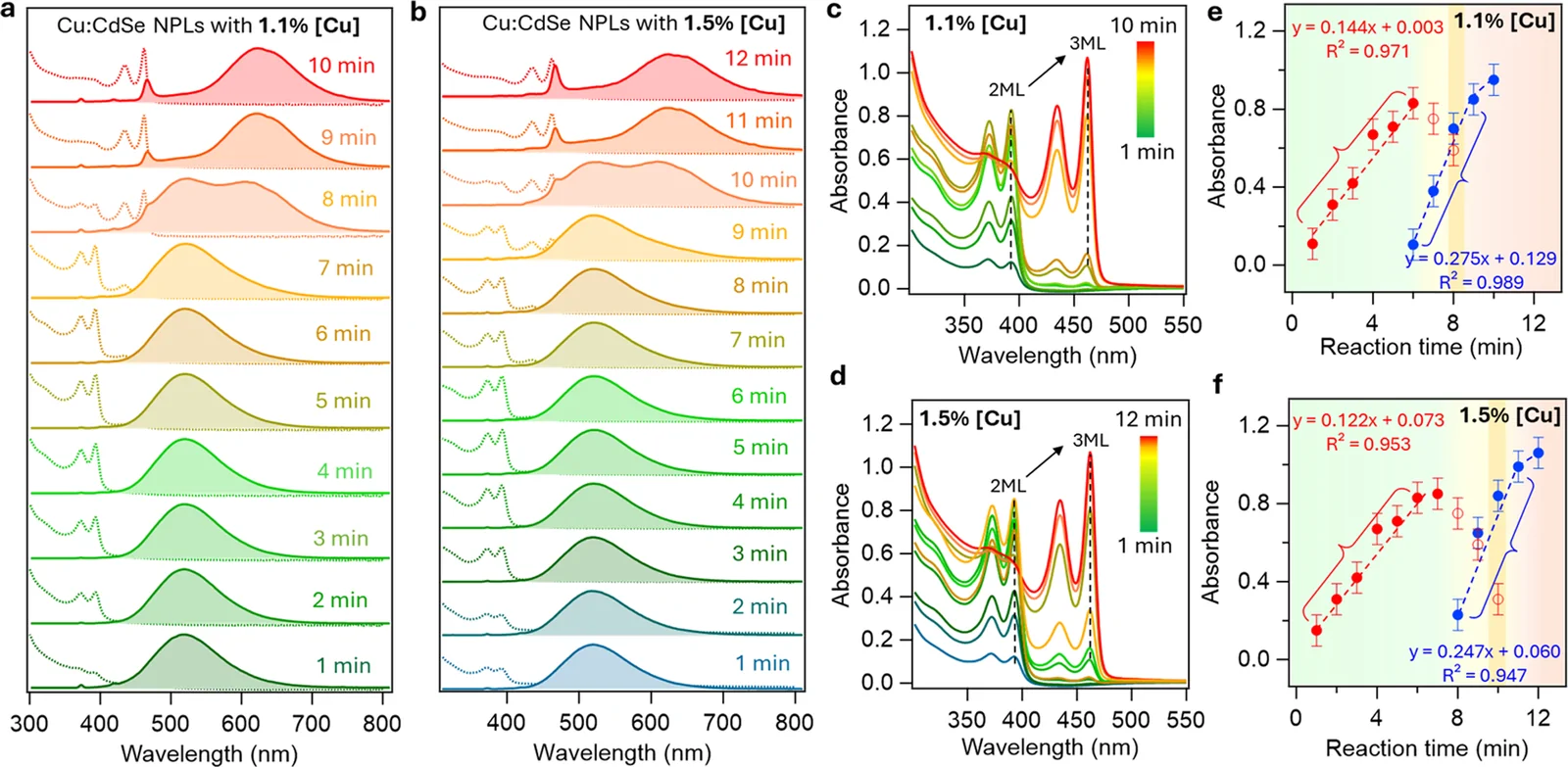
Normalized absorbance (dotted lines) and PL (solid lines) spectra of Cu:CdSe NPLs with (a) 1.1% [Cu] and (b) 1.5% [Cu]. Time-dependent absorbance with (c) 1.1% Cu and (d) 1.5% Cu is fitted by a linear relationship for 2 and 3 ML Cu:CdSe NPLs at (e) 1.1% Cu and (f) 1.5% Cu (error bars represent uncertainties from nine independent reactions).

At early reaction time, the absorbance at ∼390 nm and Cu^+^ PL at 520 nm correspond to 2 ML Cu:CdSe NPLs of the 1.1% and 1.5% Cu:CdSe NPLs. As the growth of 3 ML NPLs occurs, both absorbance and Cu^+^ PL redshift to ∼460 and 640 nm, respectively (Figure 4d). The successive, thickness-controlled growth of 2 and 3 ML Cu:CdSe NPLs at higher Cu dopant concentrations exhibits behavior similar to that shown in Figure 1. However, the complete transition from 2 to 3 ML NPLs occurs at ∼9.0 ± 0.7 min for samples with 1.1% Cu^+^ dopants, whereas it is delayed to ∼11.0 ± 0.6 min for those with 1.5% Cu^+^ dopants. This trend indicates progressively decelerated CdSe NPL growth kinetics with increased Cu^+^ incorporation (Table S2; standard deviations calculated from nine independent syntheses).

Time-dependent absorbance studies were performed for NPLs with different Cu^+^ dopant concentrations (Figure 4c,d). The absorbance intensity fitted linearly with reaction time for 1.1% and 1.5% Cu:CdSe NPLs (Figure 4e,f). However, the slope of the time-dependent absorbance of 1.5% Cu-doped 2 and 3 ML NPLs (0.122 and 0.247) is less than that of 1.1% Cu^+^ dopants (0.144 and 0.275). This reveals that the growth of Cu:CdSe NPLs with higher Cu^+^ doping concentration is relatively slow as compared to lower concentrations. This suggests that more Cu^+^ dopants could introduce additional lattice defects, leading to modified surface energy of the distinct ML and altering the reaction dynamics evident by further delayed formation of 3 ML Cu:CdSe NPLs.

### Thermal Energy-Dependent Growth of Cu:CdSe NPLs

In this study, we observed a 3-stage growth behavior of 2 and 3 ML Cu:CdSe NPLs at 230 °C. To better understand the growth of Cu:CdSe NPLs and whether it is a thermodynamically driven process, we did a temperature-dependent synthesis of Cu:CdSe NPLs at lower (200 °C) and higher temperatures (250 °C). Figure 5a depicts the absorption and PL spectra of Cu:CdSe NPLs at 200 °C, where the absorption is initially centered at ∼390 nm and Cu^+^ PL at 520 nm for 2 ML NPLs. The transition from 2 to 3 ML NPLs, exhibited by red-shifted absorbance at ∼460 nm and PL peak at 635 nm, occurs after 25 min, which is a much longer time than the transition at 6 min for the reactions at 230 °C (Figure 1a). In contrast, at a higher temperature of 250 °C, the 2 to 3 ML transition can take place more readily within only 3 min (Figure 5b). Therefore, the formation and growth of 3 ML NPLs are decelerated due to insufficient thermal energy at a lower temperature, whereas ample thermal energy quickens the 3 ML NPL growth.

**Figure 5:**
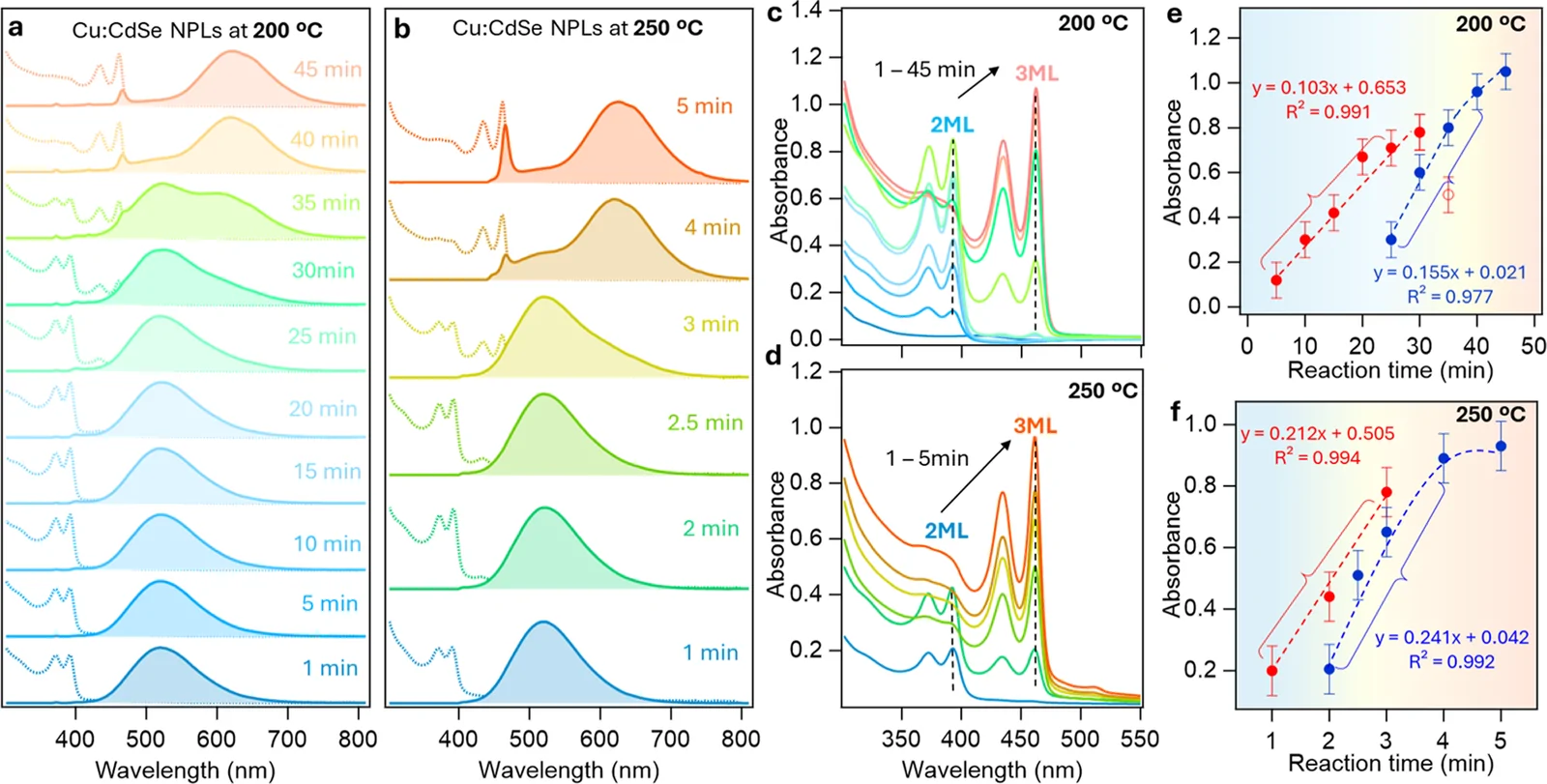
Temperature-dependent normalized absorbance (dotted lines) and PL (solid lines) of Cu:CdSe NPLs at (a) 200 °C and (b) 250 °C. Time-dependent absorbance at (c) 200 °C and (d) 250 °C is fitted by a linear relationship for 2 and 3 ML Cu:CdSe NPLs at (e) 200 °C and (f) 250 °C.

In addition, we analyzed the time-dependent absorbance of NPLs for the thickness evolution of NPLs grown at 200 and 250 °C (Figure 5c,d). The slope of the linearly fitted absorption intensity with respect to time for 2 and 3 ML Cu:CdSe NPLs synthesized at 250 °C is 0.241 and 0.212, respectively, which is almost double that of the 2 and 3 ML Cu:CdSe NPLs at 200 °C with a slope of 0.103 and 0.155, respectively (Figure 5e,f). This signifies delayed growth at lower thermal energy at 200 °C and faster growth at higher temperatures. The controlled experiment for pristine CdSe NPLs was performed at 200 °C to verify the influence of Cu^+^ ions on the growth of CdSe NPLs. Figure S15a shows the normalized absorbance at ∼460 nm and PL at 465 nm for undoped 3 ML CdSe NPLs throughout the reaction at 200 °C, which implies that even at a lower temperature, undoped CdSe directly results in 3 ML NPLs. Also, we analyzed the growth of undoped CdSe NPLs at a higher temperature (i.e., 250 °C), which directly leads to the 3 ML NPLs (Figure S15b).

We have calculated the apparent activation energy for the growth of Cu:CdSe NPLs using the Arrhenius equation (Supporting Information, Section A). The apparent activation energy at early growth stages I and II is ∼31.0 ± 3.9 kJ/mol, compared to ∼18.0 ± 5.2 kJ/mol at later growth stages II and III, indicating that Cu^+^ dopants slow the growth kinetics by raising the activation energy barrier at early stages. As Cu^+^ concentration decreases during the reaction, the activation energy is reduced, facilitating the formation of 3 ML Cu:CdSe NPLs. This suggests Cu^+^ dopant ions could modulate the surface energy of growing 2D CdSe NPLs. The early stage growth of Cu:CdSe NPLs is primarily governed by thermodynamic driving forces that favor the formation of stable, ultrathin 2 ML NPLs. As the reaction progresses, the effective Cu^+^ dopant concentration decreases, thereby reducing the dopant-induced surface energy difference between pristine and Cu-doped CdSe NPLs. Concurrently, the continued supply of Cd and Se monomers enables further growth, during which attachment kinetics and facet-dependent growth rates become increasingly important, promoting the formation of thicker 3 ML NPLs. Consequently, the subsequent evolution of 3 ML Cu:CdSe NPLs is governed by a combination of thermodynamic and kinetic factors.

Moreover, to study the possible further growth of 3 ML NPLs to even thicker NPLs at 230 °C, the Cu:CdSe NPLs were synthesized for an extended reaction time (up to 1 h) at 230 °C under the same reaction conditions. As shown in Figure S16, the 2 to 3 ML CdSe NPL transition occurs around 6 min. However, the prolonged annealing of 3 ML Cu:CdSe NPLs for 1 h did not cause 4 ML Cu:CdSe NPLs to form, which implies available thermal energy at this reaction condition is not adequate for thicker NPLs. In contrast, traces of 4 ML NPLs were observed in the absorption spectra of Cu:CdSe NPLs synthesized at 250 °C after the complete growth of 3 ML NPLs. In Figure S17, the absorption peak ∼515 nm in the tail of the absorbance spectra is attributed to 4 ML CdSe NPLs, confirming their formation at elevated temperature. This behavior can be rationalized by the higher nucleation barrier associated with the formation of 4 ML (or thicker) NPLs compared to 3 ML NPLs, such that sufficient thermal energy is required to overcome this barrier and enable further thickness growth.

### Thickness-Dependent Growth of Cu:CdSe NPLs

To further prove the influence of Cu^+^ dopants on the growth of CdSe NPLs, we doped Cu^+^ ions into thicker CdSe NPLs using Cd myristate in a modified synthesis method at 240 °C.^7^ In Figure 6a, the host PL peaks around 466 and 517 nm correspond to 3 and 4 ML CdSe NPLs with a broad Cu^+^ PL centered at 675 nm. Interestingly, the host PL ratio of 3 and 4 ML CdSe NPLs continually changes during growth of NPLs and fully changes to 4 ML NPLs PL at ∼25 min. An exponential increase in the I_4 ML_/I_3 ML_ ratio indicates the formation of 4 ML NPLs from 3 ML NPLs (Figure 6c,d), which is also evidenced by the disappearance of the 3 ML absorption peak at 466 nm and the emergence of 4 ML NPL absorption at 517 nm over reaction time (Figure 6b). It should be noted that, under the same reaction conditions, the transition from undoped 3 to 4 ML CdSe NPLs occurs within 5 min of reaction time (Figure S18). Initially, the fwhm of Cu^+^ PL is ∼95 nm (from 3 and 4 ML NPLs), which decreases to ∼80 nm with the complete formation of 4 ML NPLs around 25 min. The reduction in the fwhm of Cu^+^ PL from 3 and 4 ML NPLs (from ∼ 95 to ∼ 80 nm) is less pronounced than that observed during the transition from 2 to 3 ML NPLs (from ∼ 135 to 100 nm), due to the smaller bandgap difference between 3 and 4 ML NPLs (0.29 eV) compared with that between 2 and 3 ML NPLs (0.48 eV).^46,47^ The lifetime of Cu^+^ PL at 670 nm increases from ∼ 415 ns to ∼ 490 ns with the growth of 4 ML NPLs, similar to that previously reported for 4 ML Cu:CdSe NPLs^7^ (Figure S19 and Table S3). The emission color of the NPLs was continuously tuned from pale yellow (3 ML NPLs) to reddish orange (4 ML NPLs), as indicated by CIE coordinates (Figure 6f) and optical pictures under UV light irradiation (Figure 6a, **inset**).

**Figure 6:**
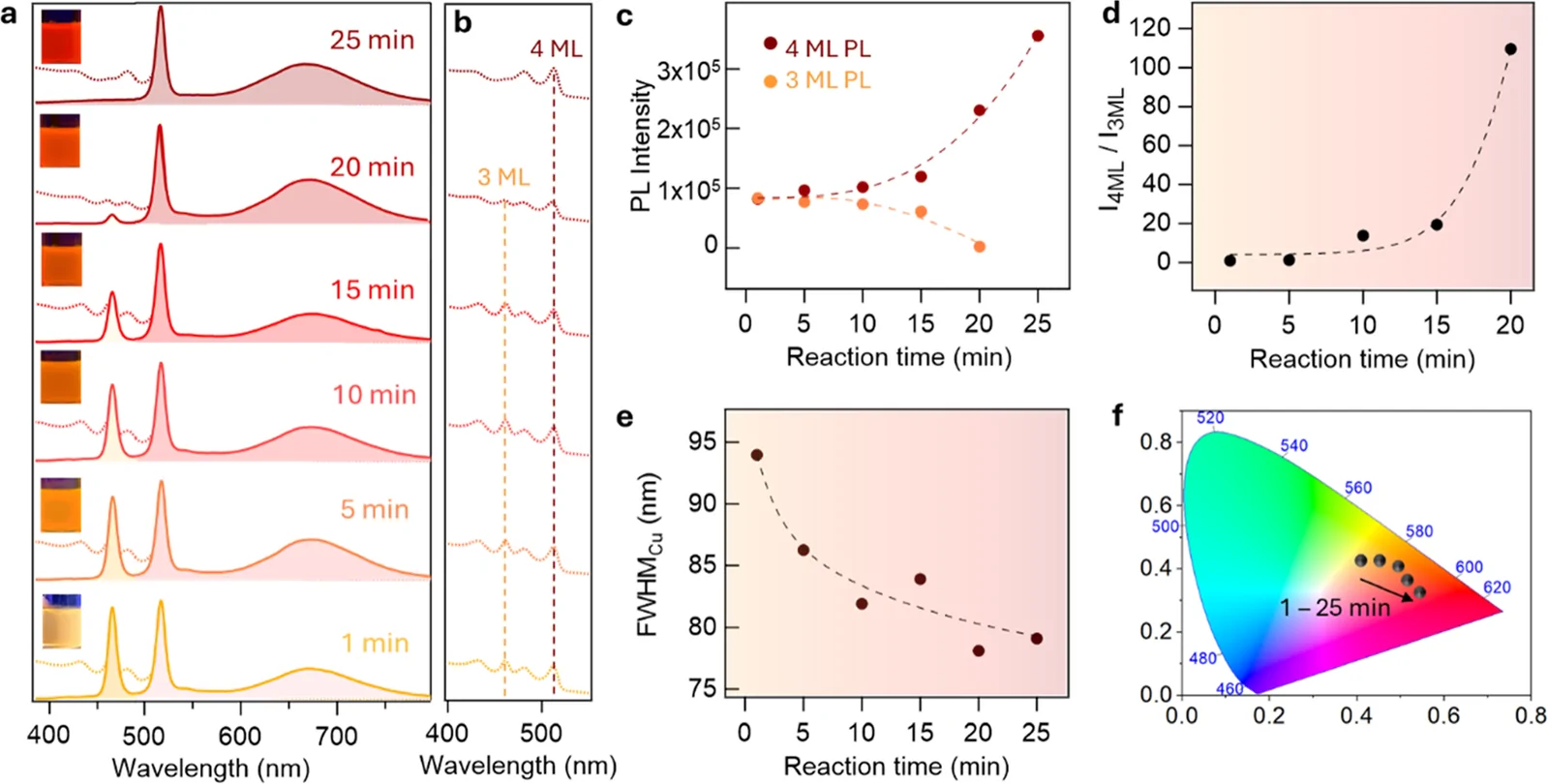
(a) Normalized absorption (dotted lines) and PL (solid lines) spectra of 3 and 4 ML Cu:CdSe NPLs with inset optical images and (b) zoomed-in normalized absorption. (c and d) PL intensity and PL peak ratio of 4 (I_4 ML_) and 3 ML (I_3 ML_) Cu:CdSe NPLs. (e and f) fwhm of Cu^+^ PL and chromaticity diagram of Cu:CdSe NPLs, respectively.

The 4 ML Cu:CdSe NPLs also possess a zinc blende structure, as evidenced in XRD (Figure S20). TEM analysis of 4 ML Cu:CdSe NPLs shows lateral growth of NPLs from ∼ 27 ± 4 to 42 ± 4 nm (Figure S21). Similar to the growth behavior observed for 2 and 3 ML Cu:CdSe NPLs, the number of Cu^+^ dopants remains nearly constant (within experimental error) for 3 and 4 MLs Cu:CdSe NPLs, leading to the decreased Cu^+^/Cd^2+^ ratio (0.8% to 0.1%) during the transition from 3 to 4 ML NPLs (Figure S22). In addition, growth of 4 ML Cu:CdSe NPLs is confirmed by the linear relationship between absorbance and reaction time similar to that of 3 ML Cu:CdSe NPLs (Figure S23).

### Growth Mechanism and Microscopic Picture of Cu:CdSe NPLs

The introduction of heterovalent Cu^+^ dopants into the CdSe lattice can induce lattice defects and Se vacancies to maintain charge neutrality in Cu:CdSe NPLs, in contrast to isovalent substitutional Mn^2+^ doping at Cd^2+^ sites, which minimally perturbs the lattice (Figure 7a).^36,48,49^ We propose that the incorporation of Cu^+^ dopant ions decreases the surface energy of NPLs, thereby increasing the nucleation barrier, which hinders the addition of Cd^2+^ ions for thicker NPLs (see further discussion in the Theoretical Simulations on the Growth of Cu:CdSe NPLs section). In the early stage, NPLs grow laterally on the side facet with a low nucleation barrier, resulting in long NPLs, as evident in TEM images (Figure 2).^46^ Eventually, the energy barrier for further adding monomers to form 3 ML NPLs drops due to the decreased Cu^+^ doping concentration and extended thermal treatment as the NPL grows bigger (inset table in Figure 7a) and results in the formation of 3 ML Cu:CdSe NPLs. On the basis of the 2D nucleation model,^46^ the energy barrier for the formation of 3 ML CdSe NPLs is 1.5 eV, which is higher than that of 2 ML CdSe NPLs with an energy barrier of 0.9 eV. Thus, rapid growth of 2 ML CdSe NPLs occurs first, leading to the more table 3 ML CdSe NPLs by ripening in the later stage of the growth.

**Figure 7:**
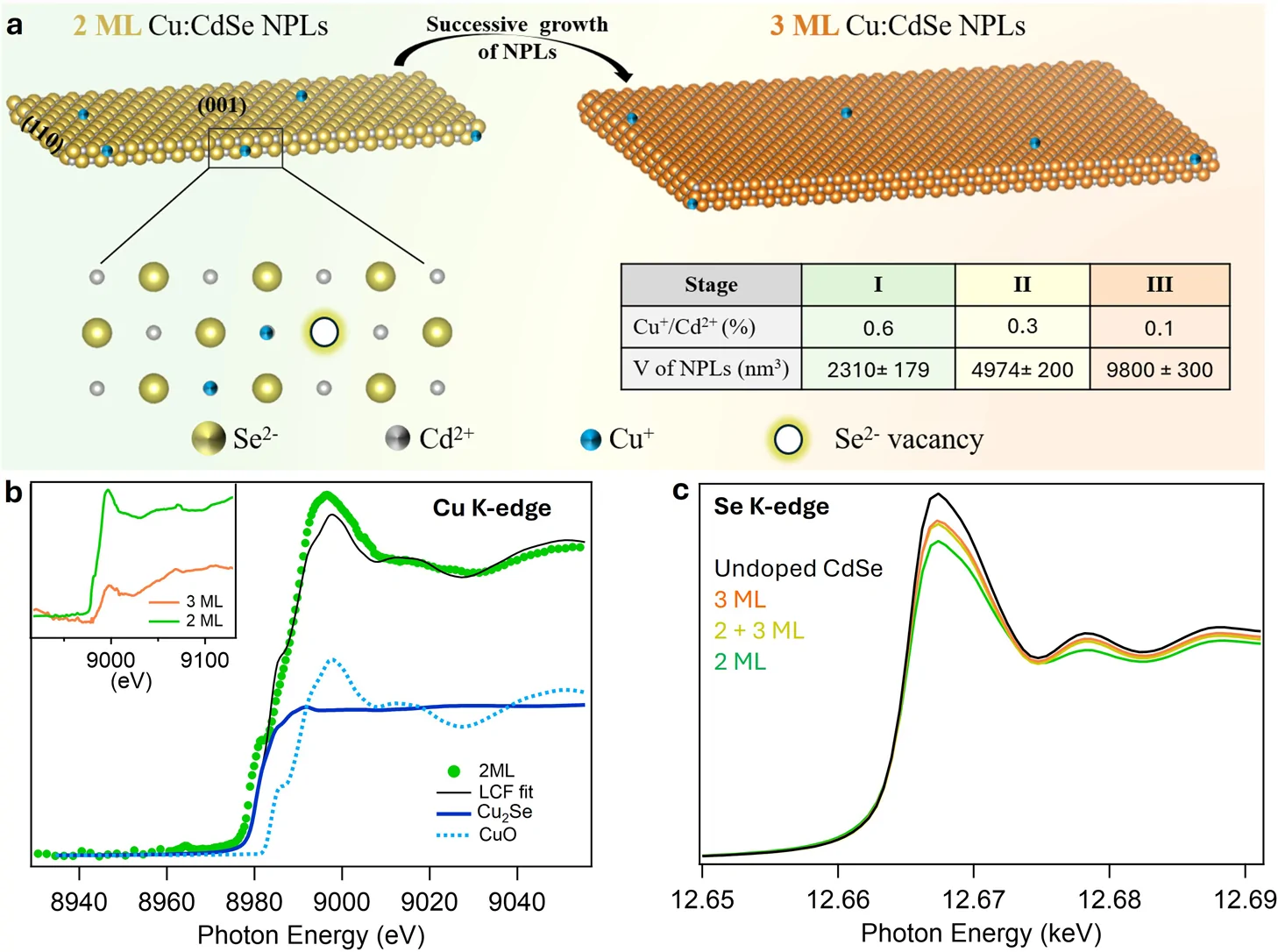
(a) Schematic of the growth mechanism for 3 ML Cu:CdSe NPLs via the formation of 2 ML NPLs. The table shows a decrease in the molar ratio of Cu^+^ and Cd^2+^ ions with lateral expansion (Volume, V) of NPLs. XAFS data for (b) Cu K-edge and (c) Se K-edge of 2 ML (stage I, green), 2 + 3 ML (stage II, yellow), and 3 ML (stage III, orange) Cu:CdSe NPLs.

To elucidate the microscopic picture underlying the growth of Cu:CdSe NPLs, we performed X-ray absorption fine structure (XAFS) measurements on undoped and Cu doped CdSe NPLs. For 2 ML Cu:CdSe NPLs (stage I), a pronounced Cu K-edge signal is clearly observed (Figure 7b), whereas only a very weak Cu K-edge signal is detectable for 3 ML Cu:CdSe NPLs (Figure 7b, **inset**). This observation is consistent with ICP-OES results, which show a decrease in the Cu^+^/Cd^2+^ ratio from 0.6% to 0.1% with an increase in NPL thickness. In addition, the XAFS spectra of 2 ML Cu:CdSe NPLs can be well fit using reference spectra of Cu_2_Se and CuO, indicating that a fraction of Cu^+^ ions is coordinated to Se^2–^, while others are partially oxidized. The CuO-like character observed under the open-air XAFS measurements suggests that a fraction of Cu^+^ dopants locate at or near the NPL surface during the early growth stage, where they are susceptible to oxidation upon air exposure.

More importantly, Se K-edge XAFS analysis (Figures 7c and S24) reveals a systematic increase in the coordination number (N) from 3.3 for 2 ML to 3.7 for 3 ML NPLs. Consistently, Cd K-edge XAFS data (Figure S25 and Table S4) exhibit a similar increasing trend in N. These results can be explained by the substitution of Cd^2+^ with Cu^+^ during stage I growth, which introduces Se^2–^ vacancies and reduces the local coordination numbers. The resulting strong electrostatic perturbation, combined with the softer Lewis acidity of Cu^+^ relative to Cd^2–^, promotes a transition in local bonding from predominantly ionic Cd–Se interactions toward more covalent Cu–Se bonding. As growth proceeds and the effective Cu^+^ concentration decreases, the lattice relaxes, leading to coordination numbers approaching those of undoped CdSe NPLs (*N* ≈ 4).

Fits to both Se and Cd K-edge XAFS data show negligible changes in the Cd–Se first-shell distance and bond lengths (Table S4), which is expected for two main reasons. First, XRD results indicate that lattice strain in NPLs is accommodated anisotropically through angular distortions and internal relaxation rather than uniform bond contraction. Thus, changes in lattice parameters inferred from XRD can coexist with minimal variations in local bond lengths measured by extended XAFS (EXAFS). Second, at low Cu concentrations (1–3%), both the Cd and Se K-edge EXAFS signals remain dominated by the majority host lattice environment, as previously reported.^50^ Although Se is intrinsically more sensitive to Cu substitution at Cd sites, the fraction of Se atoms directly coordinated to Cu remains too small to significantly alter the ensemble-averaged first-shell distance. In contrast, Se K-edge X-ray absorption near edge structure (XANES) spectra (Figure 7c) exhibit noticeable changes in near-edge intensity and line shape, indicating that even low-level Cu doping perturbs the local electronic structure around Se atoms, particularly the unoccupied Se *p*-derived states.

Monte Carlo simulations (Figure S26) further show that Cu^+^ dopants exhibit a strong preference for localization within the interior of thin 2 ML NPLs at an early growth stage, with a small fraction of Cu^+^ dopants on or near the surface. This trend is consistent across independent simulations and statistically significant. However, given that 2 ML NPLs consist of only a few atomic layers, the distinction between the core and surface becomes ambiguous. Consequently, most Cu^+^ dopants reside near or on the surface, where they significantly modulate the surface energies of the 2D NPLs. In contrast, for thicker 3 ML Cu:CdSe NPLs, the majority of Cu^+^ dopants are located within the lattice interior, consistent with nucleation doping.

It should also be noted that surface ligands can influence crystal surface energies and facilitate one- and two-dimensional nanocrystal growth.^3,51,52^ To exclude ligand effects as the primary driver of Cu:CdSe NPL growth behavior, we conducted control experiments by replacing the Cu precursor with trioctylphosphine (TOP), acetic acid, or Cd acetate (Figure S27). When TOP was used, direct formation of 3 ML CdSe NPLs occurred within 1 min, as evidenced by absorption and PL features at ∼460 nm. In contrast, substituting the Cu precursor with acetic acid and Cd acetate resulted in the simultaneous appearance of absorption peaks at ∼390 nm and ∼ 460 nm, corresponding to 2 and 3 ML CdSe NPLs, along with broad defect-related PL at ∼510 nm within 1 min. However, in these ligand-only systems, the transition from 2 to 3 ML NPLs was extremely rapid and did not exhibit the pronounced morphological evolution observed in Cu-modulated growth, which occurs over 5–7 min. These results confirm that Cu^+^ dopant incorporation, rather than ligand effects, is the dominant factor governing surface energy modulation and thickness-controlled growth in Cu:CdSe NPLs.

### Theoretical Simulations on the Growth of Cu:CdSe NPLs

To further reveal the controlled growth mechanism of the Cu:CdSe NPLs, we performed theoretical simulations on the growth behavior of CdSe NPLs as a function of Cu^+^ doping concentration and the host NPL thickness. To simulate growth, the NPL begins with a small initial nucleation site and grows through an energy-minimizing attachment procedure. At each growth step, all geometrically permissible candidate positions surrounding the current structure are identified, and the site yielding the lowest total potential energy upon trial addition is accepted. The total interatomic potential energy, computed as a sum of pairwise interatomic interactions, reflecting the relative surface stabilities and the surface stabilization energy, has been derived from potential energy. This procedure does not restrict growth to a predefined direction or surface. Instead, the structure evolves toward the most energetically favorable and locally more stable configuration at each step. The surface stabilization energy per surface atom, also termed as surface excess energy (γ), at each configuration was calculated along a collective reaction coordinate. Figure 8a indicates that the relative surface stabilization of Cu:CdSe NPLs shows a *4-fold* increase with increasing Cu^+^ concentration from 1% to 3%.

**Figure 8:**
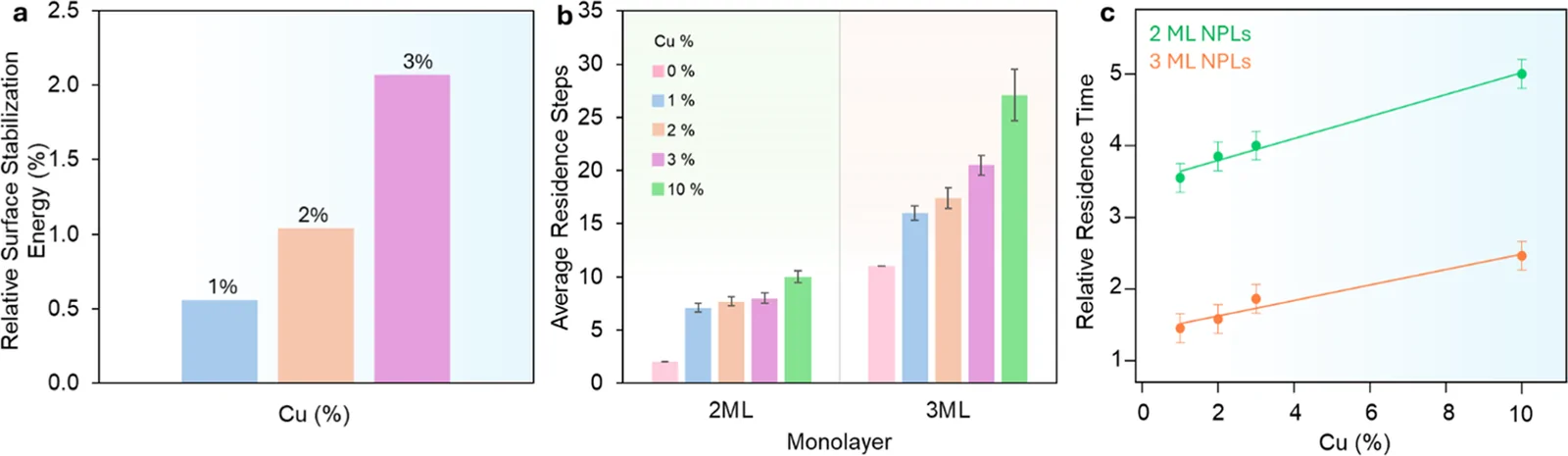
(a) Relative surface stabilization energy (%) of Cu:CdSe NPLs compared to the undoped CdSe NPLs. (b) Average residence steps at the 2 and 3 ML for undoped CdSe and Cu:CdSe NPLs. (c) Relative residence time per ML normalized with respect to *S*_res_^undoped^ across doped systems.

We recorded the growth step at which 2 ML first appears and the step at which it transitions to 3 ML. The average residence steps (*S*_res_) at each ML serve as a proxy for the structural stability and kinetic persistence of specific thickness during simulated growth. In the undoped CdSe NPLs, the value of *S*_res_< 2 at 2 ML, whereas *S*_res_ > 10 for 3 ML NPLs; a more than *5-fold* increase in *S*_res_ from 2 to 3 ML CdSe exhibits significantly longer residence at 3 ML NPLs (Figure 8b). This suggests undoped CdSe NPLs are energetically unstable at the 2 ML configuration and could follow an energy-minimizing pathway to swiftly form stable 3 ML NPLs. In contrast, Cu:CdSe NPLs show significantly higher *S*_res_ (∼8, for 1% Cu) at the 2 ML configuration than that of undoped CdSe NPLs. While higher *S*_res_ (∼15, for 1% Cu) is observed at 3 ML Cu:CdSe NPLs, the smaller difference in *S*_res_ (∼7) is observed with only a ∼ *2-fold* increase in *S*_res_ from 2 to 3 ML Cu:CdSe NPLs. Therefore, successive growth from 2 to 3 ML CdSe NPLs could be achieved by the incorporation of Cu^+^ dopants.

The *S*_res_ is also Cu^+^ doping concentration-dependent and increased from 1% to 3% in our simulation. This is consistent with the delayed 2 to 3 ML transition for a higher Cu^+^ doping concentration into CdSe NPLs, as discussed in the subsection of Impact of Cu^+^ Dopants in the Growth of CdSe NPLs (Figure 4). The relative residence time, *t*_res_, of Cu:CdSe NPLs with respect to undoped CdSe NPLs is calculated at each ML, and *t*_res_ increases linearly with the increasing Cu^+^ concentration. Interestingly, *t*_res_ at a 2 ML configuration is between 3.5 and 5, whereas *t*_res_ for 3 ML is around 1.5–2.5 (Figure 8c). The high *t*_res_ for 2 ML Cu:CdSe NPLs indicates the enhanced stability of thin 2 ML NPLs with the incorporation of Cu^+^ dopants. The Cu:CdSe NPLs show progressively longer residence steps at each ML compared with undoped CdSe NPLs, which implies the stabilization of thinner ML configurations. The Cu^+^ dopants kinetically trap the CdSe NPLs in thinner configurations by increasing the surface stabilization energy of NPLs and becoming energetically favorable. The impact of Cu^+^ dopants is enhanced by increasing their concentration and is more profound in thinner NPLs.

The simulation results are consistent with our experimental data, suggesting the delayed growth of 3 ML Cu:CdSe NPLs by introducing Cu^+^ dopants. In addition, there is a large energy difference between Cu:CdSe and pristine CdSe NPLs at stage I due to the high concentration of Cu^+^ ions and strong Cu–Se bonds.^53^ Based on the ICP-OES, the Cu^+^ dopant ion concentration decreases with the growth of NPLs (increasing volume). Therefore, as the concentration of Cu^+^ ions falls during the formation of larger NPLs, the energy difference between Cu:CdSe and pristine CdSe NPLs also drops at later stages (II and III). This facilitates the formation of 3 ML Cu:CdSe NPLs, as it replicates the same energy landscape for undoped 3 ML CdSe NPLs.

## Conclusion

In summary, we demonstrate that Cu^+^ dopant ions can effectively modulate the surface energy and, consequently, the growth kinetics of 2D CdSe NPLs. Under reaction conditions that directly yield 3 ML pristine CdSe NPLs at 230 °C, incorporation of Cu^+^ during nucleation reduces surface reactivity and suppresses immediate 3 ML formation by altering the NPL surface energy. As a result, the growth of Cu:CdSe NPLs proceeds through a distinct three-stage process, characterized by continuous red shifts in both absorption and PL: (i) formation of thin 2 ML NPLs, (ii) gradual transformation from 2 to 3 ML, and (iii) eventual formation of larger 3 ML NPLs. Notably, nucleation doping leads to a nearly constant number of Cu^+^ dopants per NPL, resulting in a decreasing dopant concentration as the NPLs grow thicker and larger. This reduction in effective dopant concentration lowers the energetic barrier for 3 ML formation, thereby facilitating the emergence of 3 ML NPLs at later reaction stages. Higher initial Cu^+^ doping further increases the NPLs’ surface stabilization energy, extending the residence time at thinner thicknesses and requiring longer growth times to reach thicker structures, while elevated reaction temperatures accelerate these transitions. XAFS analysis reveals reduced coordination numbers, which are consistent with Se-vacancy formation upon Cu^+^ incorporation into 2D CdSe NPLs. Computational simulations reveal that Cu^+^ dopants reshape the energetic landscape governing ML transitions and stabilize thinner NPL configurations relative to undoped CdSe. Overall, dopant-directed control of thickness evolution in 2D semiconductor NPLs enables precise tuning of structure and color-tunable PL, offering a versatile strategy for designing functional materials for advanced optoelectronic applications.

## Experimental Section

### Chemicals

Cadmium acetate dihydrate (Cd­(Ac)_2_·2H_2_O, >98%, Sigma-Aldrich), selenium powder (Se, >99.5% trace metal, Thermo Fisher), copper acetate anhydrous (Cu­(Ac)_2_, 98%, Alfa Aesar), TOP (tech. 90%, Thermo Fisher), myristic acid (99%, Thermo Fisher), sodium hydroxide (thermos fisher, 95%), oleic acid (OA, 90%, Sigma-Aldrich), toluene (99.6%, Pharmco), octadecene (ODE, 90%, Thermo Fisher), octylamine (>99%, Thermo Fisher), thioacetamide (99%, Thermo Fisher), chloroform (spectroscopic grade, Thermo Fisher), ethanol (≥99.5%, Pharmco), methanol (99.8%, Pharmco), and hexane (>98%, Pharmco) were purchased and used without further purification.

### Synthesis of 3 ML Cu-Doped CdSe NPLs

Three ML Cu-doped CdSe NPLs were synthesized using a modified colloidal hot injection technique following the reported procedure.^2,7,54^ In a 50 mL three-neck flask, 0.5 mmol Cd­(Ac)_2_·2H_2_O, 0.3 mmol OA, and 15 mL of ODE were added. The mixture was vacuumed at room temperature for 10 min; then, different amounts of Cu precursor (0.01 M, 1–3% doping concentration from synthesis) were added at room temperature under Ar. Subsequently, the mixture was degassed at 70 °C for 1 h, then the temperature was increased to 230 °C, and 2.5 mL of the Se-ODE precursor was swiftly injected into the solution, resulting in a gradual color change from colorless to yellow. The reaction mixture was taken at each minute for 10 min and was then cooled to room temperature using a water bath. The solution was centrifuged at 2500 rpm for 10 min, and ethanol was added to the supernatant until turbidity appeared. The solution was centrifuged again at 5000 rpm for 6 min, and then the precipitates were dissolved in toluene for optical analysis. The washing process was repeated three times for TEM, XRD, and ICP-OES measurements.

### Synthesis of 4 ML Cu-Doped CdSe NPLs

Four ML Cu-doped CdSe NPLs were synthesized using a modified colloidal hot injection technique following the reported procedure.^7^ In a 50 mL three-neck flask, 0.3 mmol of cadmium myristate, 0.15 mmol of Se, and 15 mL of ODE were added. The mixture was vacuumed at 95 °C for 1 h, and then 3 purge, degassed cycles were performed. The reaction temperature was set at 240 °C under an Ar atmosphere, and a Cu precursor (1–2%) was dropwise added at 190 °C. When the solution became yellowish, 60 mg of cadmium acetate was quickly added to the mixture. The NPLs grew at 240 °C, and reaction mixtures were taken out at different time intervals, and 0.5 mL of OA was injected. The reaction was quenched using a water bath. The solution was centrifuged at 5000 rpm for 5 min to separate out 4 ML NPLs from the mixture. The precipitates were washed with excess ethanol and toluene. The purified NPLs were used for optical characterization, and NPLs were washed three times for TEM, XRD, and ICP-OES measurements.

### Synthesis of Undoped 3 and 4 ML CdSe NPLs

Undoped CdSe NPLs were also synthesized using the above procedure except for the addition of a Cu precursor.

### Characterization

Sample size, morphology, and dispersity were analyzed by TEM using a JEOL JEM-2100F TEM operated at an accelerating voltage of 200 kV. Powder XRD patterns were taken on a Bruker D2 Phaser with a LYKXEYE 1D silicon strip detector using Cu Kα radiation (λ = 1.5406 Å). The elemental analysis of the samples was conducted by a PerkinElmer Avio-220 Max ICP-OES. An Agilent Cary 60 UV–vis spectrophotometer was used to collect absorption spectra of the samples in a 1 cm quartz cell. PL measurements were conducted with a Horiba FluoroMax Plus spectrofluorometer. Time-resolved emission measurements were performed using an Edinburgh FLS-980 fluorescence spectrometer equipped with 365 nm picosecond pulsed lasers (EPLSs). XAFS data at the Cd, Se, and Cu K-edges were recorded using fluorescence measurements (PIPS detector for the Cd and Se edges; Vortex silicon drift detector for the Cu edge) at the MRCAT beamline 10-BM at Argonne National Laboratory.^55^ Samples were ground as pure powders and loaded into Nylon plastic washers (ID 9.9 mm) using Kapton tape on both ends. The beamline was calibrated using standard metal foils for all measured absorption edges.

## Computational Methods

CdSe NPL systems with up to 4 MLs were created by replicating a CdSe unit cell to form an extended 2D slab, ensuring a stoichiometric balance and charge neutrality. Cu^+^ doping during the simulation was introduced by randomly substituting Cd^2+^ ions with Cu^+^ dopants within the NPL structure. A proportional number of Se vacancies was introduced to ensure overall charge neutrality. Four different simulation conditions of Cu:CdSe NPLs were constructed, representing 1%, 2%, 3%, and 10% Cu^+^ doping concentration with respect to the total number of Cd^2+^ sites.

### Energy Calculations and Simulations Setup

The total interatomic potential energy of each configuration was evaluated as a summation of pairwise interatomic interactions from previously published work on CdSe and β-Cu_2_Se.^56,57^ The two-body interatomic potential was approximated as a sum of Coulomb potential and Lennard-Jones potential, as shown in eq 3

3 $$U_{ij}=\frac{q_{i}q_{j}}{4\pi \varepsilon_{0}r_{ij}}+4\epsilon_{ij}\left\{{\left(\frac{\sigma_{ij}}{r_{ij}}\right)}^{12}-{\left(\frac{\sigma_{ij}}{r_{ij}}\right)}^{6}\right\}$$

here, i and j denote two interacting atoms with interatomic separation distance *r*_*ij*_ and permittivity of vacuum ε_0_. All Lennard-Jones parameters (ϵ and σ) and partial charges were obtained from previously validated empirical potentials,^56,57^ and the complete set of values is summarized in Supporting Information (Section B).

The total potential energy of the NPL is given by

4 $$U_{total}\left(\left\{r_{Cd}\right\},\left\{r_{Se}\right\},\left\{r_{Cu}\right\}\right)=\sum_{\alpha =Cd,Se,Cu}\sum_{i<j}^{N_{\alpha }}U_{ij}^{\alpha \alpha }\left(r_{ij}\right)+\sum_{\alpha <\beta =Cd,Se,Cu}\sum_{i=1}^{N_{\alpha }}\sum_{j=1}^{N_{\beta }}U_{ij}^{\alpha \beta }\left(r_{ij}\right)$$

where *N*_Cd_,*N*_Se_ and *N*_Cu_ are the number of atoms for the respective chemical species. In the following derivation, we will use the following compact notation to write the expression for the total potential energy as follows

5 $$U_{total}\left(r^{N}\right)\equiv U_{total}\left(\left\{r_{Cd}\right\},\left\{r_{Se}\right\},\left\{r_{Cu}\right\}\right)$$

here, ***r*** ^*N*^ represents the coordinates of the existing N atom NPL. Similarly, the potential energy after addition of a new atom to the NPL is denoted as *U*_total_(**r** ^*N*+1^).

### Minimum Energy Path in Growth Process

The NPL begins with a small initial nucleation site and grows through an energy-minimizing attachment procedure. At each accepted growth step, the total potential energy was normalized per atom and plotted as a function of a collective reaction coordinate (ξ), which monotonically tracks the progression of the growth trajectory. The corresponding energy-minimizing pathway for the undoped system is shown in Figure S28, illustrating a continuous picture of how stabilization proceeds as the NPL expands. The detailed procedure used to construct this collective reaction coordinate and its energy profile per atom is provided in the Supporting Information (Section B).

The thickness at each step is computed from the vertical (*z*-axis) extent of the atomic coordinates. An ML transition is detected when this thickness exceeds the threshold corresponding to the new ML formation. For each ML, we record the growth step at which it first appears and the step at which it transitions to the next ML. The difference defines the residence steps, *S*_res_, of that ML in eq 6

6 $$S_{res}=G_{End}-G_{Start}$$

where *G*_Start_ and *G*_End_ are the growth steps marking the first and last appearance of that ML to quantify how long the system remains stabilized at a particular ML thickness before transitioning to a thicker state. Residence steps were computed for the 2 and 3 ML NPLs. For doped systems, due to the inherent stochasticity of random dopant and vacancy placement, the reported residence steps represent averages over 10 independent growth simulations. To compare the stability of doped and undoped structures, a relative residence time (*t*_res_) was calculated for each ML individually, defined in eq 7

7 $$t_{res}=\frac{S_{res}^{doped}}{S_{res}^{undoped}}$$

Relative residence time was used as a metric to analyze and investigate the impact of dopants on the kinetic stability of ML formation, and the results from this analysis are presented in the Supporting Information.

## Supplementary Material



## Notes

Additional experimental details, computational methods, and characterization data including XRD, TEM, and optical data of undoped CdSe NPLs and 4 ML Cu:CdSe NPLs (PDF)
