# Supplementary material for: Dopant-Directed Surface Energy Modulation for Controlled Growth and Color-Tunable Photoluminescence in Cu+‑Doped Two-Dimensional CdSe Nanoplatelets
Source: ACS Nano. 2026 Aug 5;20(32):22851–64. doi: 10.1021/acsnano.6c08297 (PMC13492462; doi:10.1021/acsnano.6c08297)
Supplement: Supplementary file 1 [file nn6c08297_si_001.pdf]

Supporting Information for

Dopant-Directed Surface Energy Modulation for Controlled Growth and Color-Tunable Photoluminescence in Cu^{+}-Doped Two-Dimensional CdSe Nanoplatelets

Vanshika Vanshika,[a] *Sara Talebi,*[a] *Joshua T. Wright,*[b] *Poojan Kaswekar,*[c] *Anthony Moses,*[a] *Yuchen Zhang,*[c] *Emily Qiao,*[a] *Robert W. Meulenberg,*[d] *Arindam Chakraborty,*[a]* *Weiwei Zheng* [a]*
[a]Department of Chemistry, Syracuse University, Syracuse, New York 13244, United States
[b]Department of Physics, Illinois Institute of Technology, Chicago, Illinois 60616, United States
[c]Department of Mechanical and Aerospace Engineering, Syracuse University, Syracuse 13244, United States
[d] Department of Physics and Astronomy and Frontier Institute for Research in Sensor Technologies, University of Maine, Orono, Maine 04469, United States

AUTHOR INFORMATION

Corresponding Author

*Email: wzhen104@syr.edu (Weiwei Zheng)

*Email: archakra@syr.edu (Arindam Chakraborty)

Table of contents

A. Experimental

B. Computational Methods

C. Figures and Tables

Figure S1. (a) Normalized absorption (dotted lines) and PL (solid lines) of undoped 3 ML CdSe NPLs with optical images on the right side. (b and c) PL peak and FWHM of band-edge PL of 3 ML CdSe NPLs. (d and e) PL QY and chromaticity coordinates of undoped 3 ML CdSe NPLs, respectively.

Figure S2. Photoluminescence quantum yield (PL QY) of Cu:CdSe NPLs.

Figure S3. TEM images and length histograms of undoped 3 ML CdSe NPLs at (a, d) 3 min, (b, e) 6 min, and (c, f) 10 min reaction time.

Figure S4. TEM images of Cu:CdSe NPLs at different reaction times from 1 to 10 min, indicating 2D morphology.

Figure S5. High-resolution TEM images show a d-spacing value of ~0.25 nm for the cubic (220) plane of Cu:CdSe NPLs.

Figure S6. (a) Normalized absorbance of Cu:CdSe NPLs (dotted line) and Cu:CdSe/CdS core/shell NPLs (solid lines). TEM images of Cu:CdSe/CdS core/shell NPLs at (b) Stage I, (c) Stage II, and (d) Stage III.

Figure S7. XRD pattern of undoped 3ML CdSe NPLs as a function of time.

Figure S8. (a)XRD patterns of Cu:CdSe NPLs at different reaction times. XRD analysis of full width at half maxima (FWHM) of (b) (111) diffraction peak, and (c) (220) diffraction peak of the Cu:CdSe NPLs as a function of reaction time.

Figure S9. Fitting of (220) XRD peak into broad (202) and sharp (220) components for 2 and 3 ML Cu:CdSe NPLs.

Figure S10. (a) Decreasing Cu^{+}/Cd^{2+} ratio in Cu:CdSe NPLs with an increase in reaction time from 1 to 10 min from inductively coupled plasma optical emission spectroscopy (ICP-OES). (b) Volume of NPLs analyzed from TEM images (pink) and number of Cu^{+} dopants per NPL in Cu:CdSe NPLs (blue) as a function of reaction time.

Figure S11. (a) Cu^+ dopant PL and (b) Cu^+ dopant PL lifetime decay for Cu:CdSe NPLs with two PL peaks ~520 nm and ~635 nm during transition from 2 to 3 ML NPLs at 5 to 7 min reaction times.

Table S1. The lifetime decay parameters of Cu^+ dopant PL for 2 and 3 ML Cu:CdSe NPLs at three growth stages.

Figure S12. (a) Time-dependent absorbance at 465 nm is fitted by (b) a linear relationship for 3 ML CdSe NPLs.

Figure S13. Cu^+/Cd^{2+} ratio from ICP-OES analysis of Cu:CdSe NPLs at higher initial Cu doping concentration (1.1% and 1.5%).

Figure S14. XRD pattern of Cu:CdSe NPLs with (a) 1.1% [Cu] and (b) 1.5% [Cu].

Table S2. Time of the complete transition to 3 ML Cu:CdSe NPLs among 9 repeated reactions.

Figure S15. Normalized absorption (dotted lines) and PL (solid lines) of undoped 3 ML CdSe NPLs at (a) 200 ºC and (b) 250 ºC.

Figure S16. Normalized absorbance (dotted lines) and PL (solid lines) of Cu:CdSe NPLs annealed at longer reaction time (~1 h).

Figure S17. Time-dependent absorbance of Cu:CdSe NPLs at 250 ºC.

Figure S18. Normalized absorption (dotted lines) and PL (solid lines) of undoped 4 ML CdSe NPLs.

Figure S19. PL lifetime decay of Cu^+ PL at 670 nm is increasing with the formation of 4 ML Cu:CdSe NPLs.

Table S3. The lifetime decay parameters of Cu^+ dopant PL for 3 and 4 ML Cu:CdSe NPLs at three growth stages.

Figure S20. XRD pattern of 4ML Cu:CdSe NPLs as a function of reaction time.

Figure S21. TEM images, size histograms of length, and width of 4 ML Cu:CdSe NPLs at (a-c) 1 min, (d-f) 15 min, and (g-i) 25 min reaction time.

Figure S22. ICP-OES of 4 ML Cu:CdSe NPLs at different reaction time.

Figure S23. (a) Time-dependent absorbance and (b) linear relationship of 4 ML Cu:CdSe NPLs.

Figure S24. Se K-edge (a) k^2-weighted EXAFS, $k^2c(k)$ and (b) magnitude of the Fourier-transformed EXAFS, $|c(R)|$, of 2 ML (Stage I, green), 2+3 ML (Stage II, yellow), and 3 ML (Stage III, orange) Cu:CdSe NPLs.

Figure S25. Cd K-edge (a) XANES, (b) k^2-weighted EXAFS, $k^2c(k)$, and (c) magnitude of the Fourier-transformed EXAFS, $|c(R)|$, of 2 ML (Stage I, green), 2+3 ML (Stage II, yellow), and 3 ML (Stage III, orange) Cu:CdSe NPLs.

Table S4. Summary of Cd and Se K-edge XAFS fits including coordination number (N), bond length (R), mean-squared relative displacements (σ^2), and energy shifts (ΔE_0).

Figure S26. The ratio of number of Cu^+ inside the inner cube and total number of Cu^+ in the NPLs ($N\alpha/NT$) as a function of scale factor, α.

Figure S27. Normalized absorbance (dotted lines) and emission (solid lines) of ligand-dependent synthesis of CdSe NPLs using (a) TOP and ODE, (b) TOP, ODE, and acetic acid, and (c) $Cd(OAc)_2$, TOP, and ODE.

Figure S28. Potential energy per atom as a function of the collective reaction coordinate (ξ) for the undoped CdSe system. The curve reflects the energy-minimizing pathway followed during nanoplatelet growth and is truncated to the first 200 accepted growth steps.

A. EXPERIMENTAL

Synthesis of Se precursor. 3 mmol Se powder and 30 mL ODE were added into a 50 mL three-neck flask. The solution was degassed for 1 h at room temperature and heated to 180 ºC under Argon (Ar). The reaction kept stirring at 180 ºC for overnight (24 h). After 24 h, the solution was cooled to room temperature and stored in the dark before use.

Synthesis of Cu precursor. In a 20 mL vial, 0.08 mmol $Cu(Ac)_2$, 2 mL TOP, and 5 mL ODE were added. The 0.01 M Cu precursor solution was degassed for 1 h at room temperature and heated to 90 ºC under Ar. The reaction was stirred at 90 ºC for 24 h and stored in the dark after completion for further use.

Cadmium myristate. In a 500 ml flask, 6 mmol sodium hydroxide, 6 mmol myristic acid was dissolved in 240 mL methanol at room temperature for 15 minutes. Then, solution of cadmium nitrate (2 mmol) in 40 ml was added dropwise to the first solution. The solution was stirred vigorously till turbidity. After that, the precipitates were washed three times with methanol and dried under vacuum overnight.

Unrolling of Cu:CdSe NPLs. 400 µL of as-synthesized NPLs were dissolved in 2mL chloroform. 20 mg of thioacetamide (TAA) and 200 µL octylamine were added to the mixture. The solution was centrifuged for ~5 min till the complete dissolution of TAA and color changed to yellow to orange. Then, 0.1 ML $Cd(oleate)_2$ solution in octylamine (200 µL) was added and reaction allowed to proceed for ~35-40 min at room temperature. The precipitates were collected using a few drops of ethanol and suspended in chloroform. Hexane was used as solvent for the TEM analysis. To improve the NPLs dispersion, the NPLs were capped with $Cd(oleate)_2$ and isolated with UV light for 1 h.

Calculation of Cu^+ ions/ NPL

Lattice constant for zinc blende CdSe, a = ~0.627 nm from XRD measurement

Unit cell volume of zinc blende CdSe, $V_{unit\ cell} = a^3 = (0.627)^3 = 0.246\ nm^3$

Cd atoms per unit cell = 4

Volume of NPL, VNPLs calculated from TEM data

# of Cu atoms per NPL = $V_{NPLs} / V_{unit\ cell}$ x 4 x $Ratio_{Cu/Cd}$ (Note: $Ratio_{Cu/Cd}$ is the ratio of Cu/Cd from ICP-OES measurement)

Table A1. Calculation of Cu^+ ions per NPL.

Stage	V_{NPLs} (nm^3)	$Ratio_{Cu/Cd}$	# of Cu/ NPL
I	2310± 179	0.007 ± 0.0005	263 ± 28
II	4974± 200	0.003 ± 0.0005	243 ± 42
III	9800 ± 300	0.001 ± 0.0001	239 ± 20

Calculation for the lattice parameters of Cu:CdSe NPLs

The symmetry of cubic CdSe is reduced to tetragonal because of limited MLs of atoms along the thickness direction and non-uniform lattice strain (**Figure A1**). Therefore, the lattice parameters in lateral (a) and thickness (c) direction were calculated using equation:

$$\frac{1}{d^2} = \left(\frac{h^2 + k^2}{a^2}\right) + \left(\frac{l^2}{c^2}\right)$$

$$\frac{1}{d_{lateral}^2} = \left(\frac{2^2 + 2^2}{a^2}\right) + \left(\frac{0^2}{c^2}\right)$$

$$\frac{1}{d_{thickness}^2} = \left(\frac{2^2 + 0^2}{a^2}\right) + \left(\frac{2^2}{c^2}\right)$$

where a and c are lattice parameter in lateral and thickness direction, respectively d is the d-spacing.

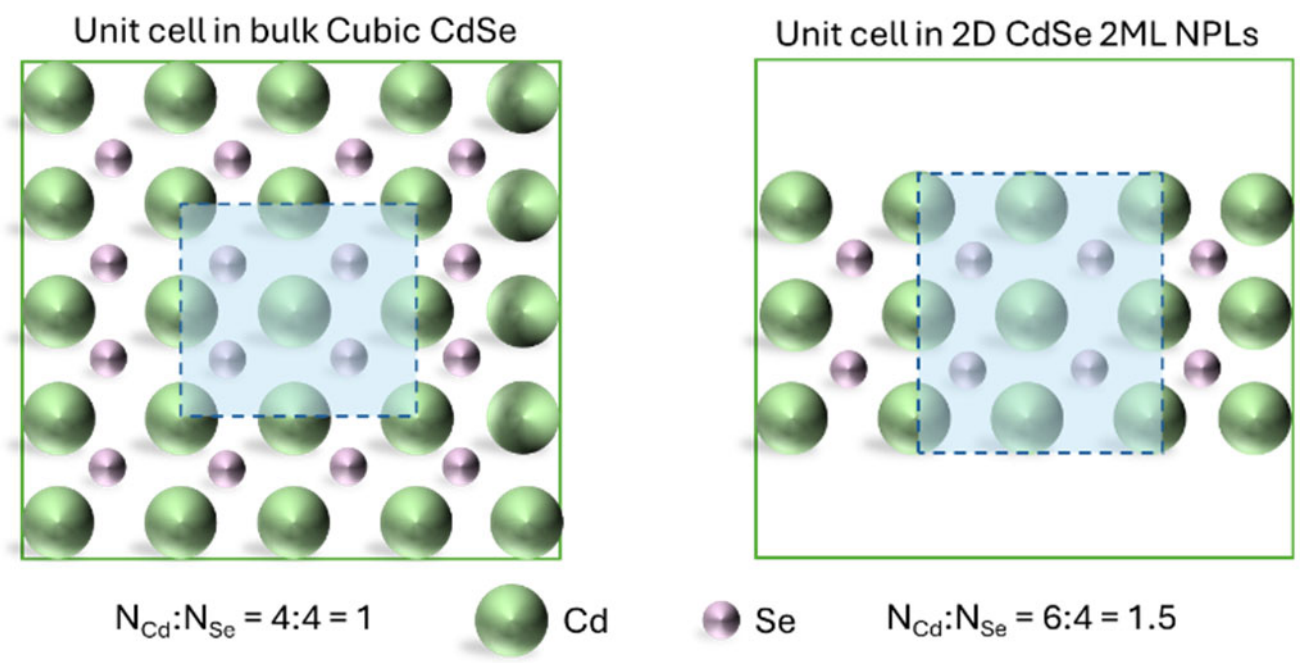

Figure A1. Difference of unit cells between bulk cubic CdSe and 2 ML CdSe NPLs.

Table A2. Lattice parameter calculation for 2 and 3 ML Cu:CdSe NPLs using (220) and (202) diffraction plane

ML	2θ		d (Å)		$a_{lateral}$ (Å) (220)	$c_{thickness}$ (Å) (202)
	(220)	(202)	(220)	(202)		
2	41.4	42.7	2.224	2.162	6.356	5.986
3	41.8	42.9	2.204	2.153	6.212	5.947

Calculation of activation energy for the growth of Cu-doped CdSe NPLs

The activation energy of Cu:CdSe NPLs is calculated using temperature-dependent absorbance data in Figure 5e-f.

$$y = kx + b$$

where y is absorbance, x is reaction time (min), k is apparent rate (abs/min), b is intercept
Arrhenius equation:

$$k = A_0 e^{-Ea/RT}$$

where k is rate constant, A_0 is pre-exponential factor, E_a is activation energy (J/mol), R is gas constant (8.314 J/mol/K), T is absolute temperature (K)

$$\text{Also, } \ln\left(\frac{k2}{k1}\right) = \frac{Ea}{R}\left(\frac{1}{T1} - \frac{1}{T2}\right)$$

where T_1 and T_2 are temperatures at 200 °C and 250 °C, respectively with corresponding rate constants k_1 and k_2.

Table A3. Apparent rates and activation energy of growth of Cu:CdSe NPLs

	200 °C (abs/min)	250 °C (abs/min)	Ea (kJ/mol)
Early growth (Stages I & II)	0.103 ± 0.006	0.212 ± 0.017	~ 31.0 ± 3.9
Later growth (Stages II & III)	0.155 ± 0.016	0.240 ± 0.015	~18.0 ± 5.2

B. COMPUTAIONAL METHODS

S1. Interatomic Potential Parameters Used in CdSe and Cu-Doped CdSe Simulations

All Lennard-Jones (ϵ and σ) and Coulomb (partial charge) parameters used in this work are summarized in **Table B1**. Mixed Lennard-Jones parameters for cross-atomic interactions were computed using Lorentz-Berthelot mixing rules of $\sigma_{ij} = \frac{\sigma_i+\sigma_j}{2}$ and $\epsilon_{ij} = \sqrt{\epsilon_i \epsilon_j}$. A 10 Å cutoff was applied to all Lennard-Jones interactions.

Table B1: Partial charges and Lennard-Jones parameters used for Cd, Se, and Cu atoms.

	q	σ (˚A)	ϵ (eV)
Cd	+1.18	1.98	0.00145
Se	−1.18	5.24	0.00128
Cu-Se	+0.59*	2.17	0.4370

* Charge applies only to Cu; Se retains −1.18.

S2. Minimum Energy Path in Growth Process.

In this work, we are interested in evaluating the minimum energy pathway (MEP) for the growth of the NPLs.[1, 2] Because of the high dimensionality of the configuration space, we use a stochastic gradient method for finding the MEP.[1, 3] The minimum energy path for NPL growth was constructed using equation:

$$\mathbf{r}^{N+1} \leftarrow \underset{\alpha,\boldsymbol{\eta}}{\operatorname{argmin}} \left[U_{\text{total}}(\mathbf{r}^N + \alpha\boldsymbol{\eta}) \right]$$

Here, $\boldsymbol{\eta}$ is the unit vector and α is the adjustable scaling parameter. To reduce computational, we discretize the continuous search space and use real-space grid points. As a consequence of that, the set of values for $\boldsymbol{\eta}$ is not continuous, but is obtained from a finite discrete set $\{\boldsymbol{\eta}_1, \boldsymbol{\eta}_2, \ldots, \boldsymbol{\eta}_M\}_N$ where M is the number of unit vectors. However, we note that this set depends on the total number of atoms N, and the set of unit vectors sampled changes with each addition of atom to the NPL. In this work, $M = 10^6$ was used which implies that each atom-by-atom growth step was obtained after sampling energies along a one million directions and performing line-minimization along α. We use a stochastic scheme to make the overall minimization problem tractable. Starting with the initial structure containing N atoms with the coordinates $\mathbf{r}^N$, a guess structure for an NPL with N+1 atoms is generated by randomly placing the incoming atom at location $\alpha\boldsymbol{\eta}$. The new configuration is then defined as $\mathbf{r}_{\text{g}}^{N+1} = \mathbf{r}^N + \alpha\boldsymbol{\eta}$, followed by line-minimization with respect to α. After that, a new direction that is orthogonal to $\boldsymbol{\eta}$ is randomly selected and the procedure is repeated. New structures that minimize energy are saved and structures that result in increase in total energy are discarded.

At each new saved structure, the total potential energy was normalized per atom and plotted as a function of a collective reaction coordinate (ξ), which monotonically tracks the progression of the growth trajectory. The corresponding energy-minimizing path for the undoped system is shown in **Figure S28**, illustrating a continuous picture of how stabilization proceeds as the nanoplatelet expands. The detailed procedure for constructing this collective reaction coordinate and its per-atom energy profile is described in Section S3.

S3. Construction of the Collective Reaction Coordinate and Minimum Energy Growth Profile

To visualize the energetic progression of the growth pathway, we computed the energy profile per atom along a collective reaction coordinate, denoted ξ, as illustrated in **Figure S28**. This coordinate represents the cumulative progression of accepted growth steps and provides a continuous mapping from the initial nucleus to the final expanded nanoplatelet. At each growth step, a trial atom was added to all geometrically valid surface sites, and the resulting structures were evaluated using the same total potential energy model employed throughout this study. The candidate configuration with the minimum total energy was accepted as the next state of the system. After acceptance, the total potential energy of the configuration was normalized per atom.

The reaction coordinate (ξ) used in this work is constructed from the hyperradius of the nanoplatelet at each accepted growth step. Initially, the system consists only of the seed cluster. For a configuration with N atoms, the hyperradius R_N is computed directly from the atomic Cartesian coordinates. This usage is closely analogous to hyperspherical treatments of few- and many-body systems, where a single hyperradius serves as a global collective coordinate parameterizing the system size and the associated potential energy surface.[4-6] No reduced mass or effective mass weighting is applied, since all atoms are treated with equal importance. Therefore, the hyperradius is obtained solely from the spatial distribution of atoms, defined as:

$$R_N = \sqrt{\sum_{i}^{N} x_i^2 + y_i^2 + z_i^2}$$

The hyperradius has an intuitive geometric interpretation: it is small when nucleation begins at the initial seed and increases steadily as atoms attach and the structure grows. These properties make the hyperradius a natural reaction coordinate, as it provides a smooth, monotonic mapping of the geometric evolution of the system into a single scalar variable.[6, 7] The unitless reaction coordinate was then constructed as:

$$\xi_N = \frac{R_N}{1\,nm}$$

S4. Surface Stabilization Energy Calculation

To quantify the effect of Cu doping on nanoplatelet stability, we calculate the surface excess energy per surface atom, γ, following an atom-resolved formulation.[8] The surface excess energy, is defined as:

$$\gamma = \frac{1}{N_{\mathrm{surf}}} \sum_{i=1}^{N_{\mathrm{surf}}} \left[U_i - N_{\mathrm{surf}} \bar{U}_{\mathrm{bulk}} \right] = \bar{U}_{\mathrm{surf}} - \bar{U}_{\mathrm{bulk}}$$

where N N_{surf} is the number of surface atoms, and U_i is the interaction energy associated with surface atom i, arising from its interactions with the remaining N-1 atoms The bulk reference energy per atom, $\overline{U}_{bulk}$ is obtained by averaging the total interaction energy, ($U_{tot}(\boldsymbol{r}^N)$), over all atoms and $\overline{U}_{surf}$ is the average interaction energy per surface atoms. This atom-resolved formulation enables direct evaluation of local energetic variations at the surface and is particularly suitable for doped and finite-sized systems with heterogeneous surface environments. To compare the relative stability of doped and undoped systems, we compute the relative surface stabilization energy with respect to the undoped nanoplatelet,

$$Relative\ Surface\ Stabilization\ energy\ (\%)\ = \frac{\gamma_{0\%} - \gamma_{[Cu]\%}}{\gamma_{0\%}} \times 100$$

where $\gamma_{0\%}$ and $\gamma_{[Cu]\%}$ corresponds to the undoped and Cu-doped NPLs, respectively. The results from the surface energy analysis revealed the Cu-doping increases the surface stabilization energy of the NPL.

S5. Spatial Distribution of Cu^+ Dopants

To further quantify the spatial distribution of Cu dopants within the CdSe NPLs, we introduce a volume-based concentration ratio defined over an inner subregion of the NPL. Specifically, we define a cubic region centered within the NPL, in which dimensions are scaled by a factor $\alpha \in [0,1]$ relative to the full NPL dimensions.

For an NPL with dimensions $L_x \times L_y \times L_z$, the inner cube is defined as $\alpha L_x \times \alpha L_y \times \alpha L_z$.

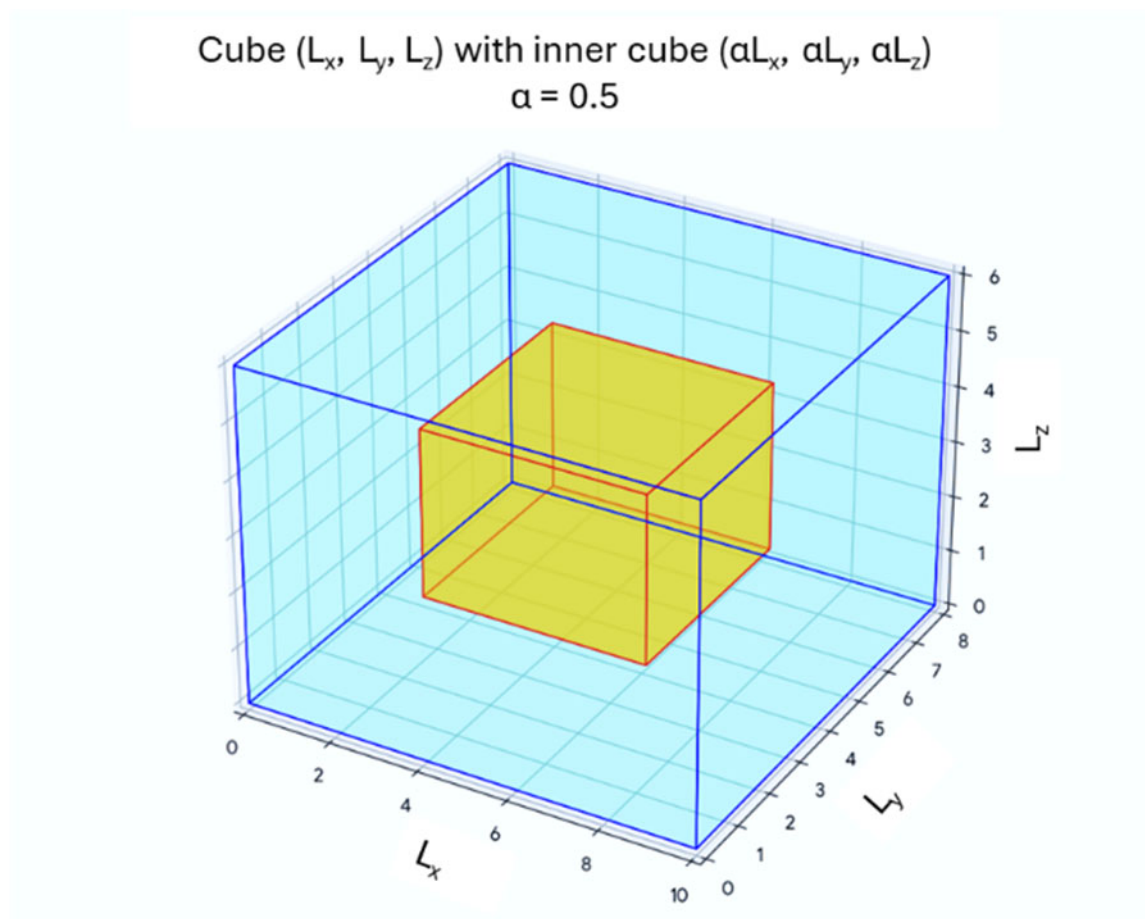

For each value of α, we computed the ratio:

$$R(\alpha) = \frac{N_{Cu}^{inner}(\alpha)}{N_{Cu}^{total}}$$

Where, $N_{Cu}^{inner}(\alpha)$ is the number of Cu atoms located within the inner cube, and N_{Cu}^{total} is the total number of Cu atoms in the full NPLs. By construction,

$$\lim_{\alpha \to 1} R(\alpha) = 1$$

This approach provides a quantitative analysis of dopant localization. A linear scaling of $R(\alpha)$ with the cube volume ($\propto \alpha$^3) would indicate a uniform spatial distribution of Cu atoms.

If the dopant is homogeneously distributed then

$$R_{\text{homo}}(\alpha) = \frac{N_\alpha}{N_T} = \frac{\alpha^3 V\rho}{V\rho} = \alpha^3$$

Deviations from this behavior indicate preferential localization, such as clustering toward the center or accumulation near the surface of the NPLs. The results from the stochastic simulation show deviation from the homogeneous scaling law.

C. FIGURES

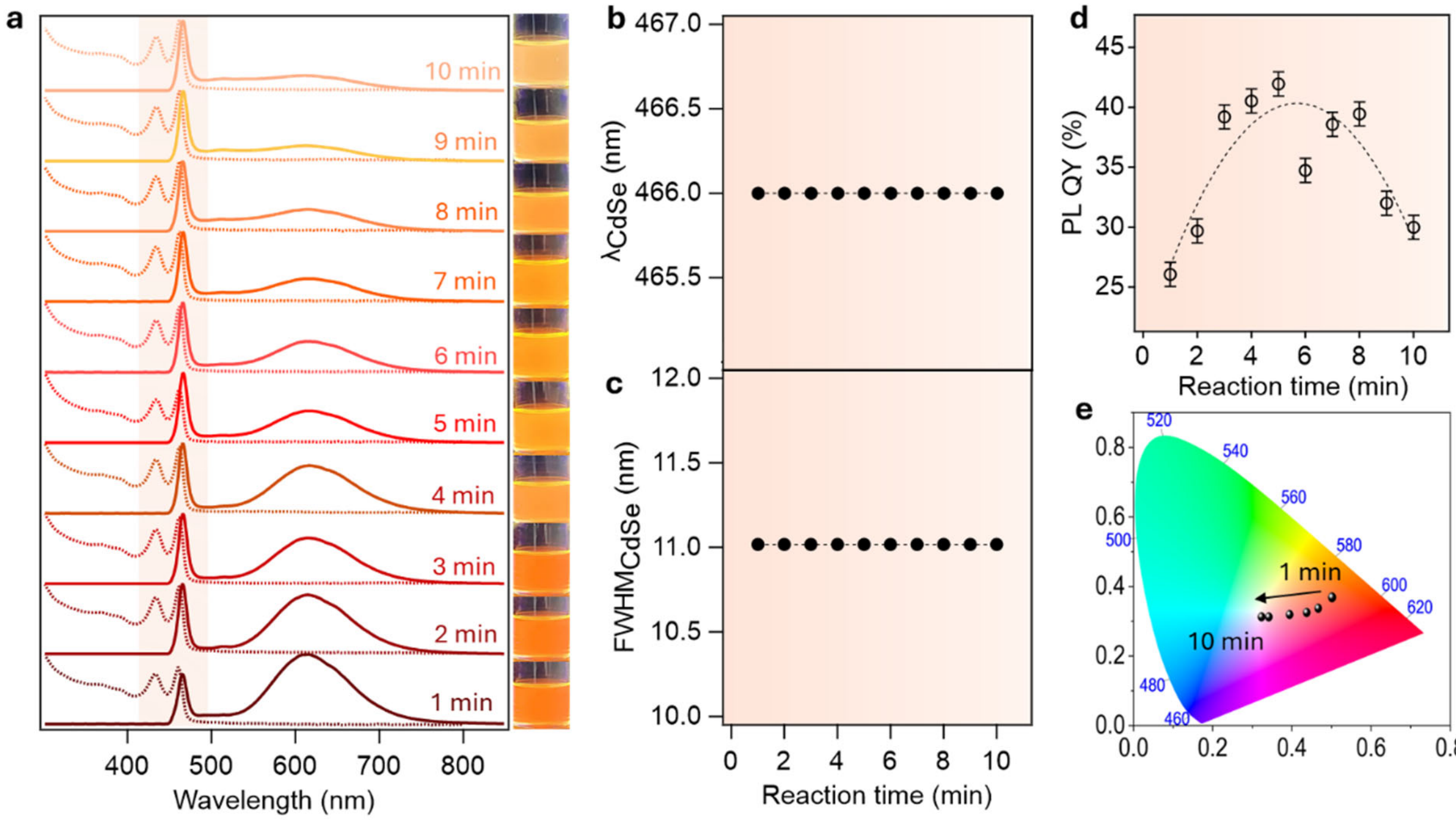

Figure S1. (a) Normalized absorption (dotted lines) and PL (solid lines) spectra of undoped CdSe NPLs with optical images on the right side. (b and c) PL peak and FWHM of band-edge PL of CdSe NPLs. (d and e) PL QY and chromaticity coordinates of undoped CdSe NPLs, respectively.

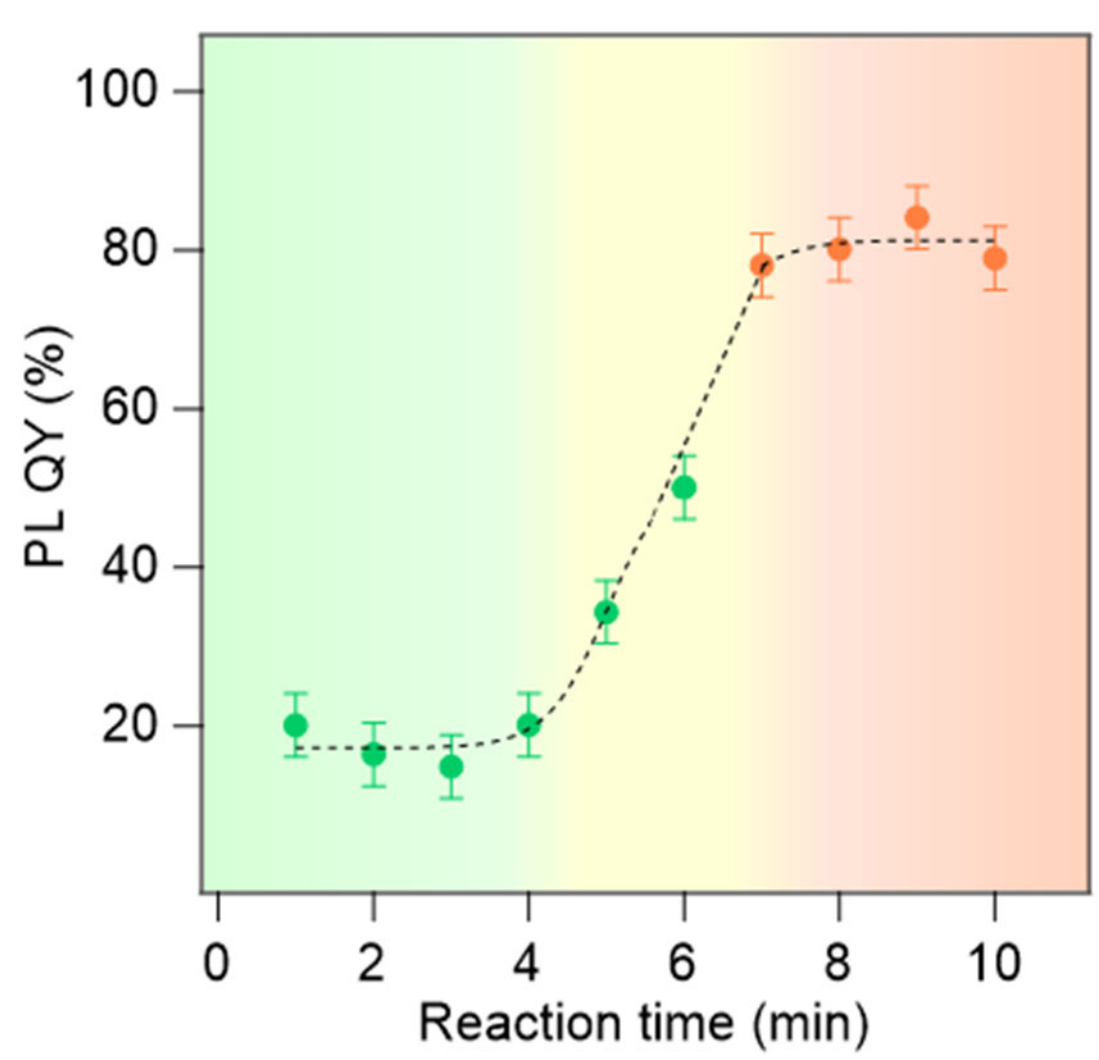

Figure S2. Photoluminescence quantum yield (PL QY) of Cu:CdSe NPLs.

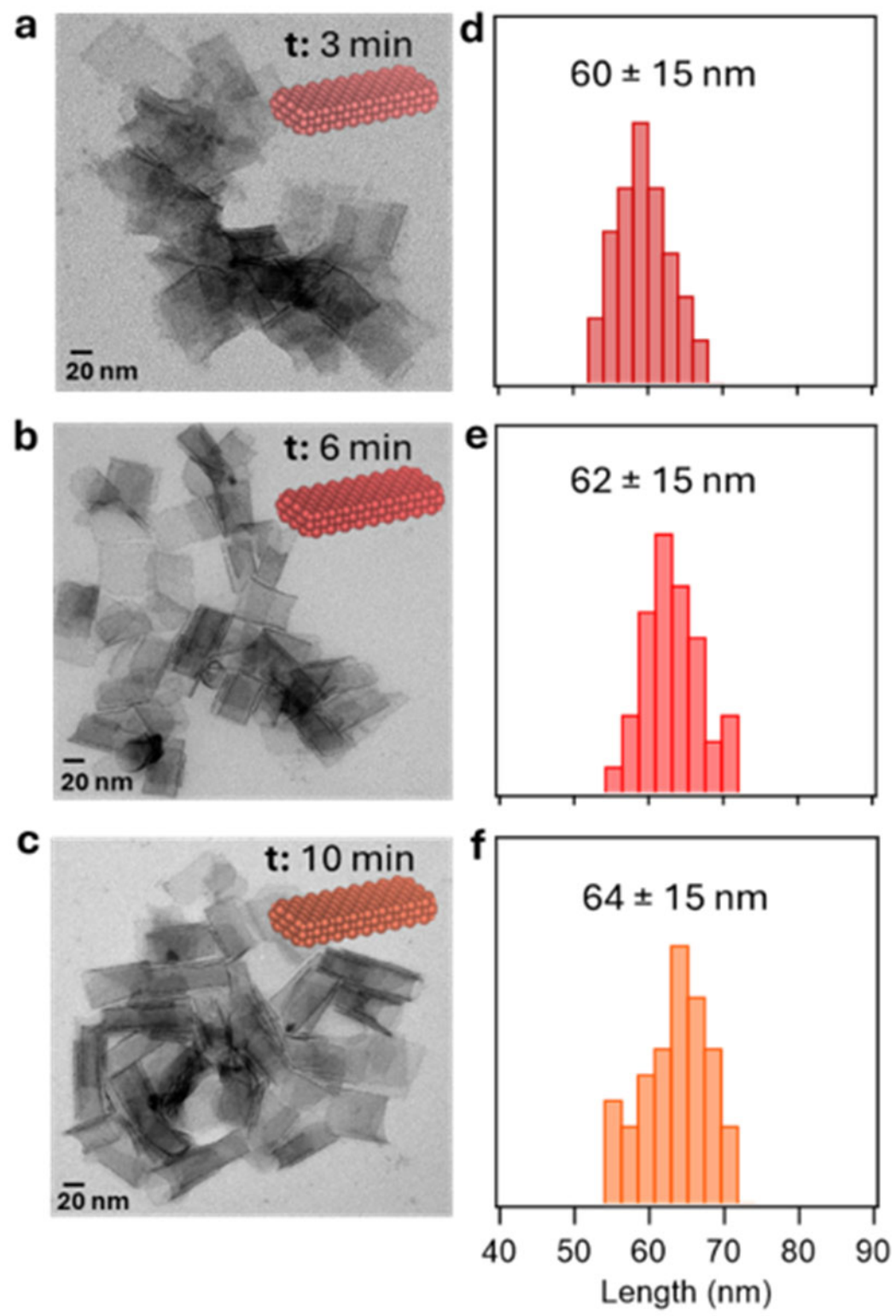

Figure S3. TEM images and length histograms of undoped 3 ML CdSe NPLs at (a, d) 3 min, (b, e) 6 min, and (c, f) 10 min reaction time.

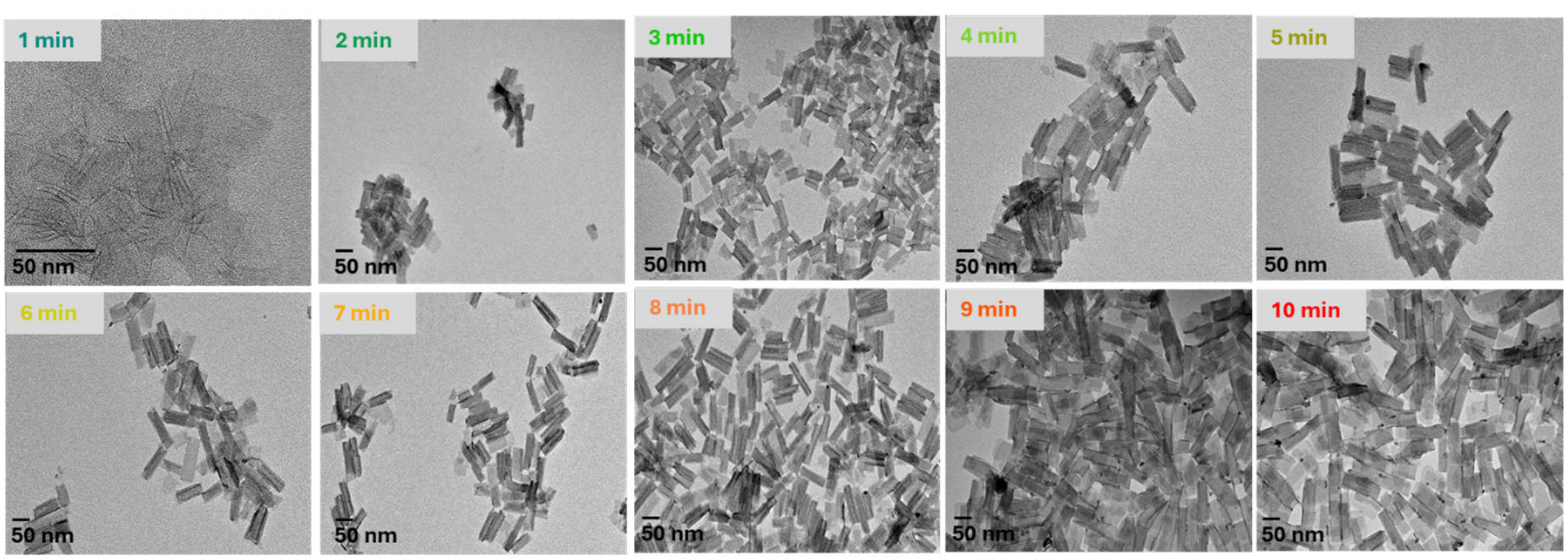

Figure S4. TEM images of Cu:CdSe NPLs at different reaction times from 1 to 10 min, indicating 2D morphology.

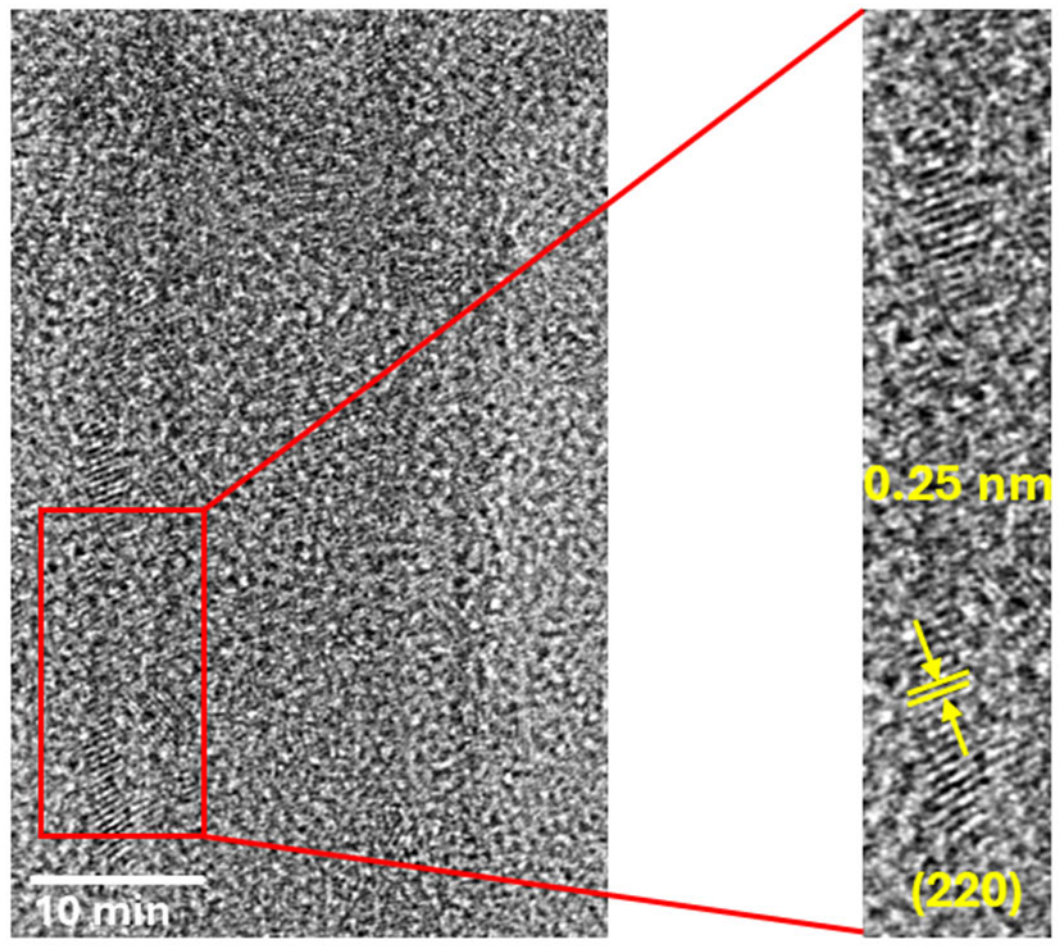

Figure S5. High-resolution TEM images show a d-spacing value of ~0.25 nm for the cubic (220) plane of Cu:CdSe NPLs.

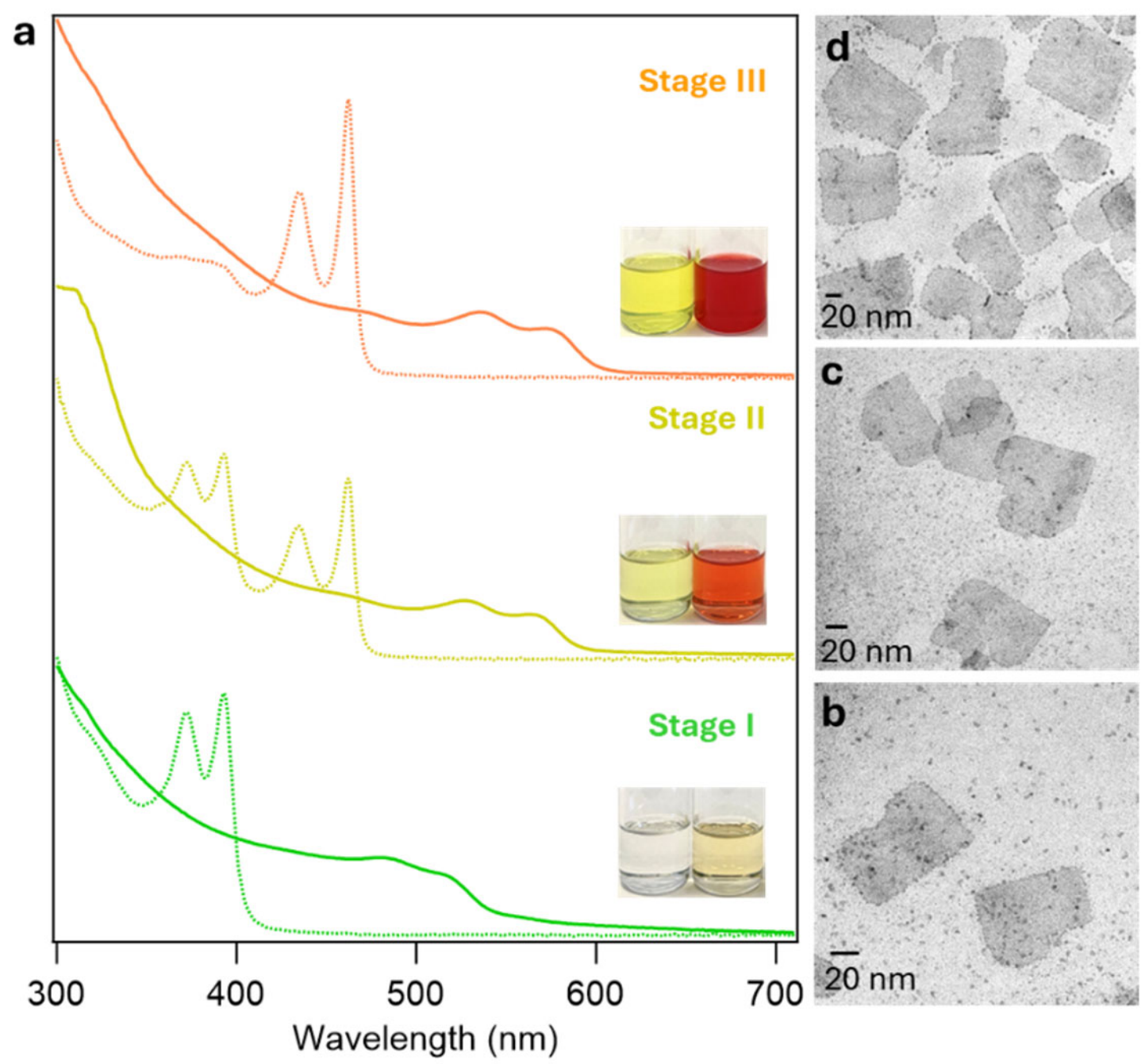

Figure S6. (a) Normalized absorbance of Cu:CdSe NPLs (dotted line) and Cu:CdSe/CdS core/shell NPLs (solid lines). TEM images of Cu:CdSe/CdS core/shell NPLs at (b) Stage I, (c) Stage II, and (d) Stage III.

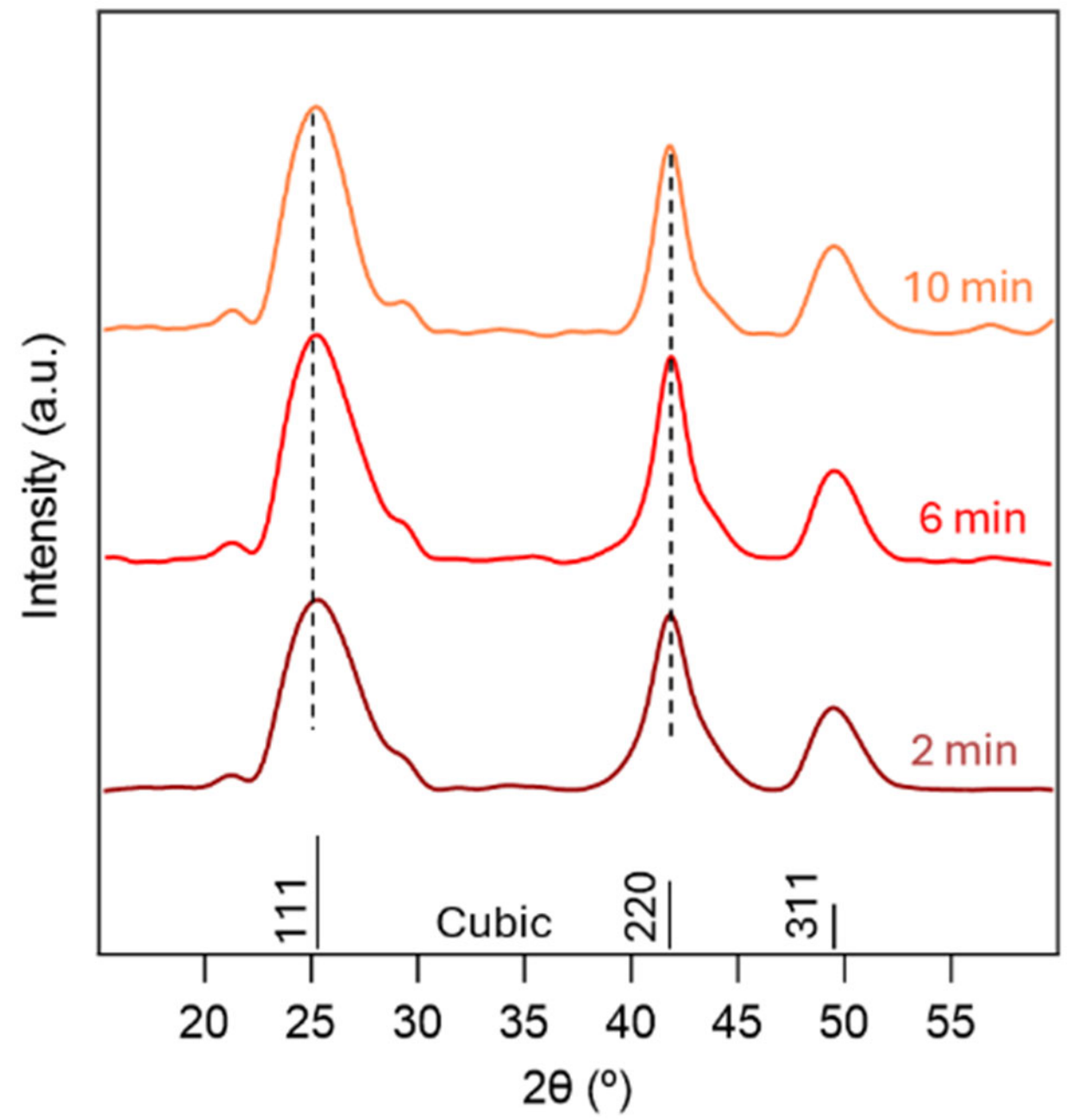

Figure S7. XRD pattern of undoped 3 ML CdSe NPLs as a function of reaction time.

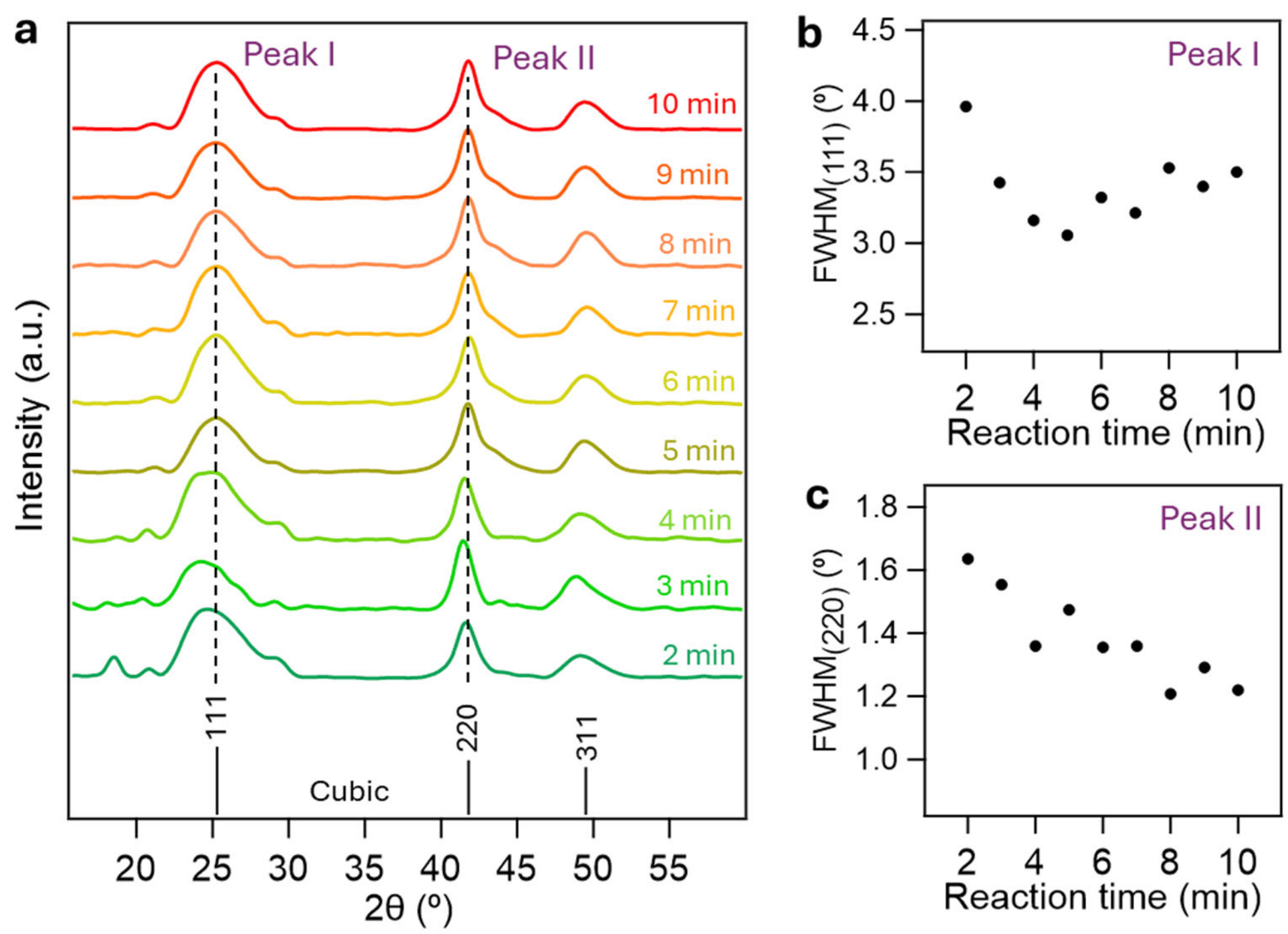

Figure S8. (a)XRD patterns of Cu:CdSe NPLs at different reaction times. XRD analysis of full width at half maxima (FWHM) of (b) (111) diffraction peak, and (c) (220) diffraction peak of the Cu:CdSe NPLs as a function of reaction time.

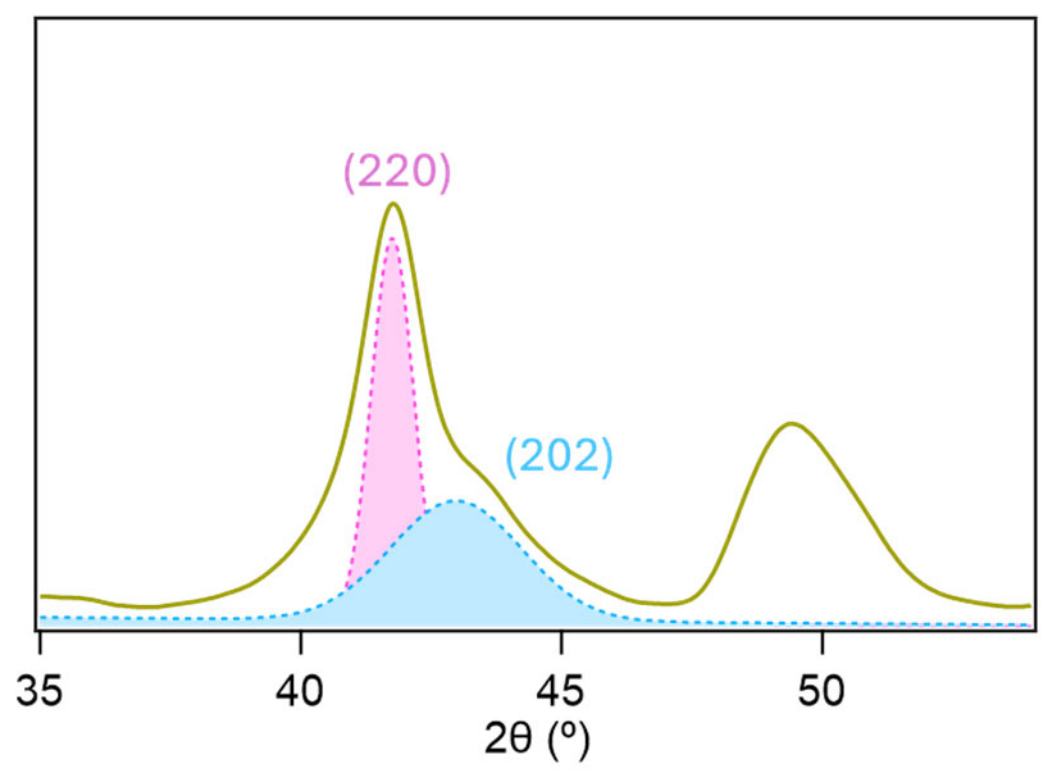

Figure S9. Fitting of (220) XRD peak into broad (202) and sharp (220) components for 2 and 3 ML Cu:CdSe NPLs.

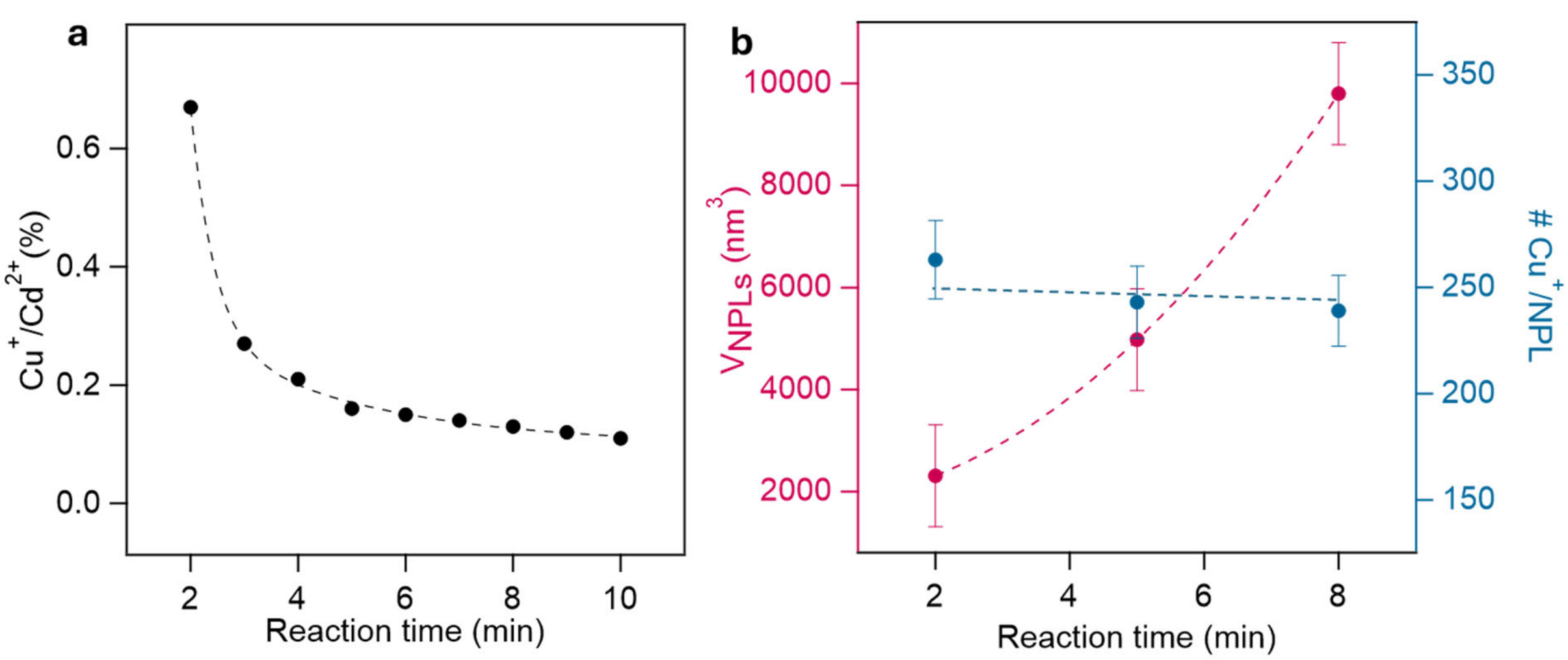

Figure S10. (a) Decreasing Cu^{+}/Cd^{2+} ratio in Cu:CdSe NPLs with an increase in reaction time from 1 to 10 min from inductively coupled plasma optical emission spectroscopy (ICP-OES). (b) Volume of NPLs analyzed from TEM images (pink) and number of Cu^{+} dopants per NPL in Cu:CdSe NPLs (blue) as a function of reaction time.

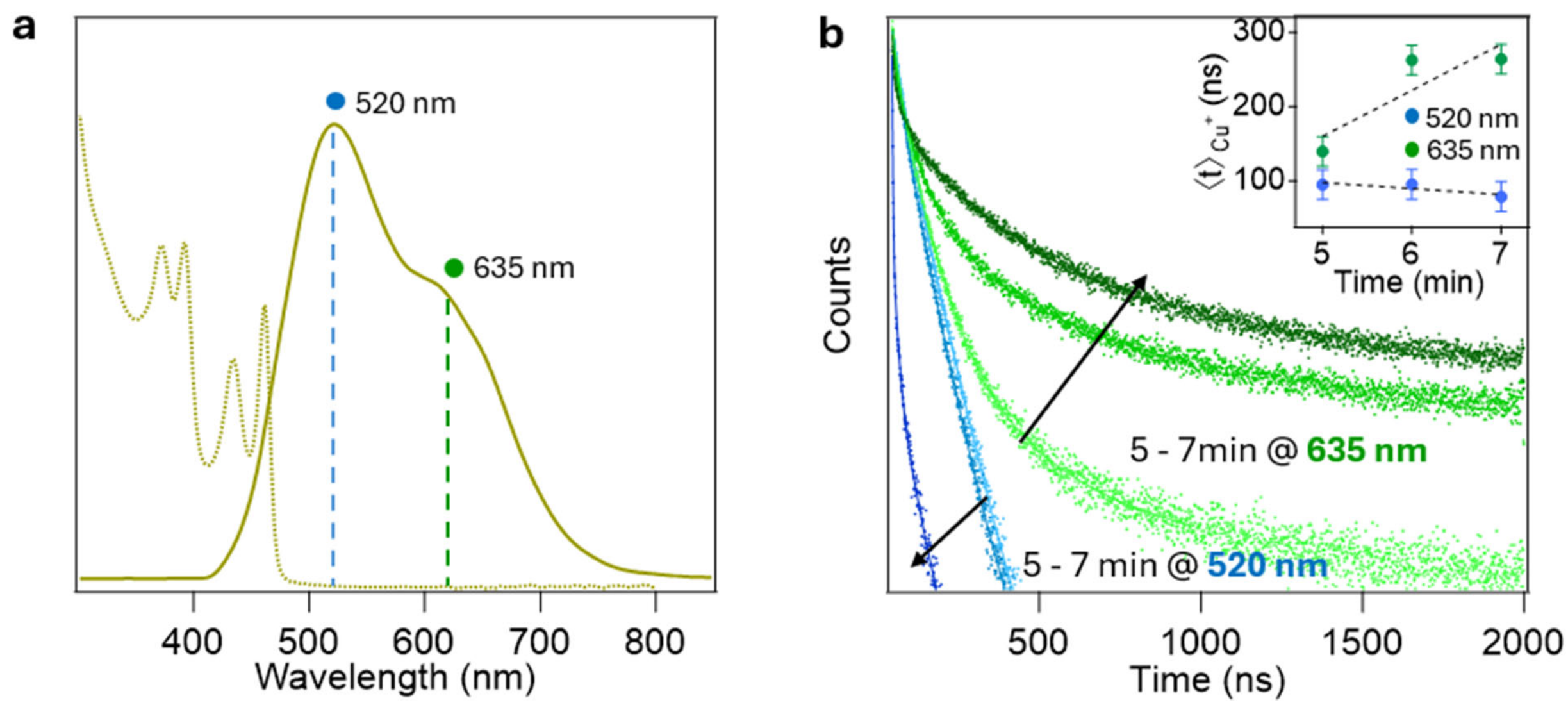

Figure S11. (a) Cu^{+} dopant PL and (b) Cu^{+} dopant PL lifetime decay for Cu:CdSe NPLs with two PL peaks ~520 nm and ~635 nm during transition from 2 ML to 3 ML NPLs at 5 to 7 min reaction times.

Table S1. The lifetime decay parameters of Cu^{+} dopant PL for 2 and 3 ML Cu:CdSe NPLs at three growth stages.

Stages	Time (min)	τ_1 (s)	%	τ_2 (s)	%	τ_3 (s)	%	τ_1 (s)	%	τ_2 (s)	%	τ_3 (s)	%
I	1	1.77E-08	13.3	6.39E-08	72.1	2.13E-07	14.6						
	2	2.93E-08	12.7	8.33E-08	78.9	2.92E-07	8.4						
	3	3.17E-08	15.5	8.59E-08	78.1	3.58E-07	6.3						
	4	1.78E-08	4.8	7.43E-08	79.5	1.76E-07	15.6						
II	5	3.26E-08	2.6	8.76E-08	43.7	3.86E-07	53.6	1.81E-08	6.7	7.31E-08	59.2	2.79E-07	34.1
	6	4.37E-08	2.3	9.32E-08	36.1	4.05E-07	61.8	1.56E-08	3.2	7.35E-08	30.5	3.62E-07	66.2
	7	1.74E-08	2.6	6.40E-08	37.2	1.27E-07	60.1	5.12E-09	1.5	3.86E-08	7.3	2.86E-07	91.2
III	8							4.65E-09	1.6	3.61E-08	7.5	2.60E-07	90.8
	9							6.83E-09	2.2	7.22E-08	12.6	3.40E-07	85.2
	10							4.52E-09	1.7	3.89E-08	7.7	2.62E-07	90.5

Stage I & II: 520 nm Cu^{+} PL from 2 ML NPLs

Stage II & III: 635 nm Cu^{+} PL from 3 ML NPLs

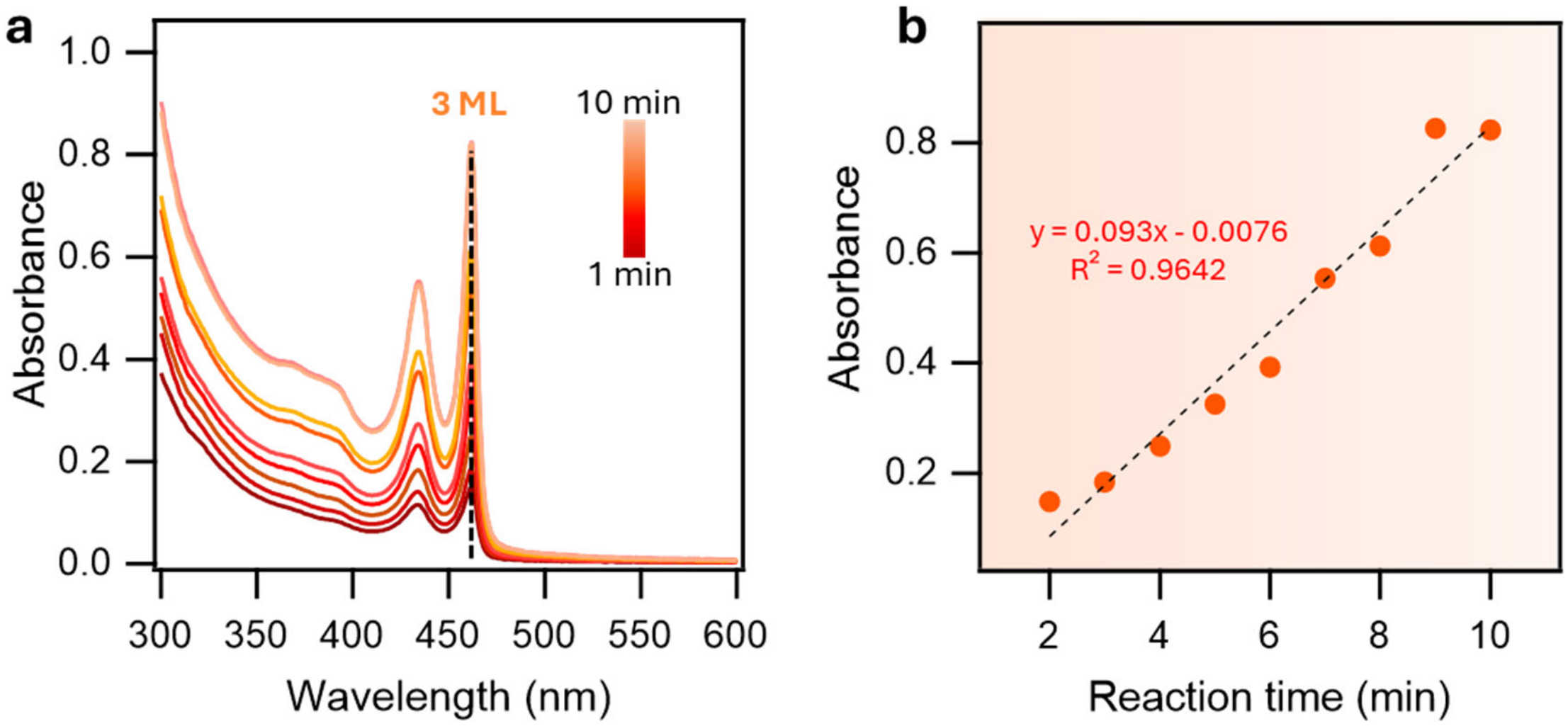

Figure S12. (a) Time-dependent absorbance at 465 nm is fitted by (b) a linear relationship for 3 ML CdSe NPLs.

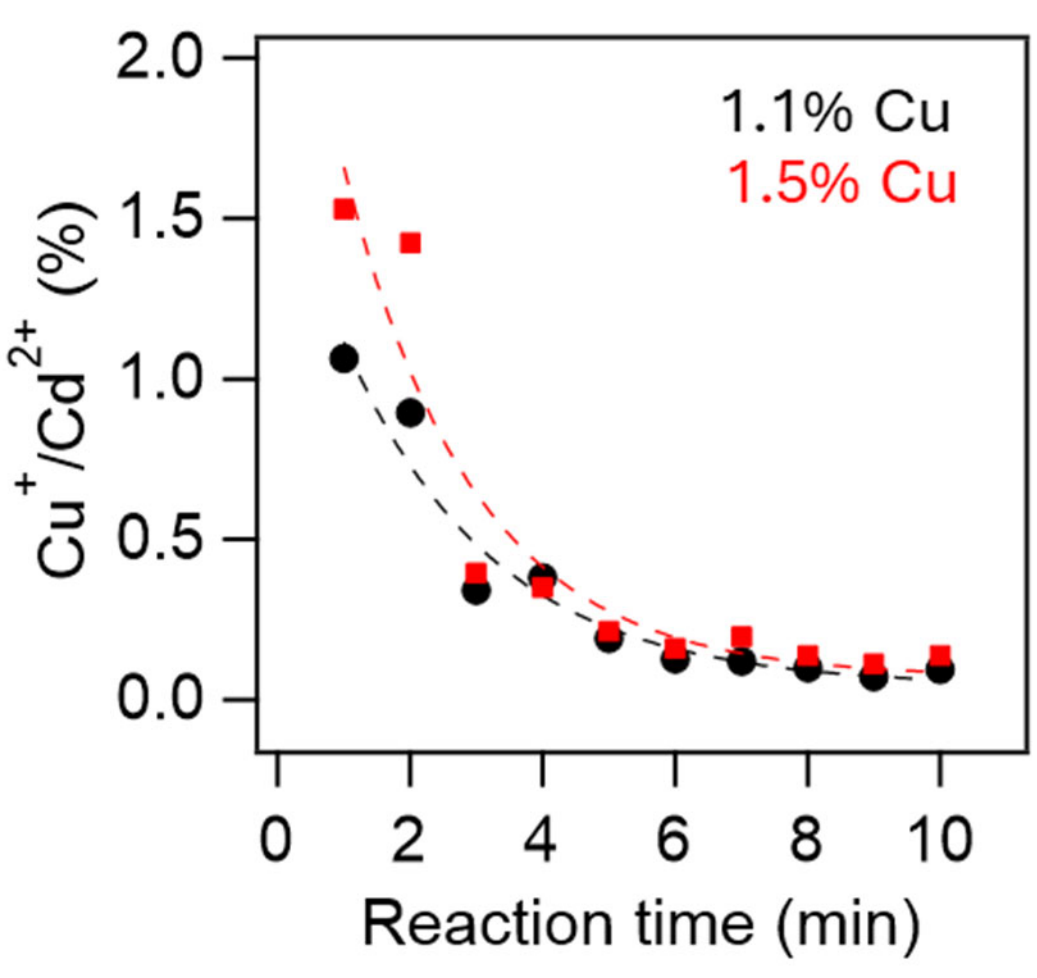

Figure S13. Cu^+/Cd^{2+} ratio from ICP-OES analysis of Cu:CdSe NPLs at higher initial Cu doping concentration (1.1% and 1.5%).

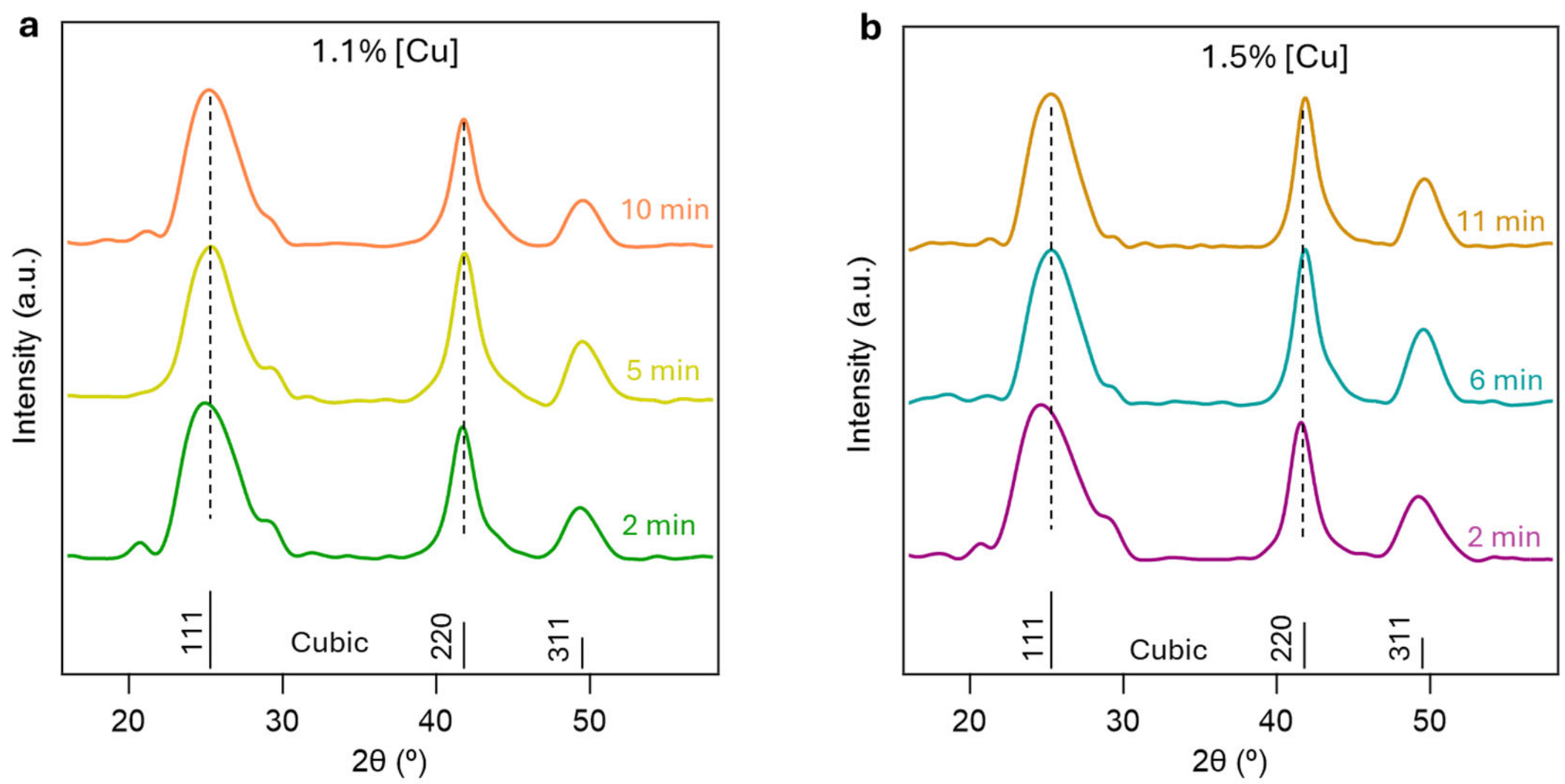

Figure S14. XRD pattern of Cu:CdSe NPLs with (a) 1.1% [Cu] and (b) 1.5% [Cu].

Table S2. Time of the complete transition to 3 ML Cu:CdSe NPLs among 9 repeated reactions.

	**Transition time (min)**	
**S.NO.**	**1.1 % Cu**	**1.5% Cu**
1	9	11
2	8	11
3	9	12
4	10	12
5	8	11
6	9	10
7	10	11
8	8	11
9	9	12
Average	8.9	11.2
Standard deviation	0.7	0.6

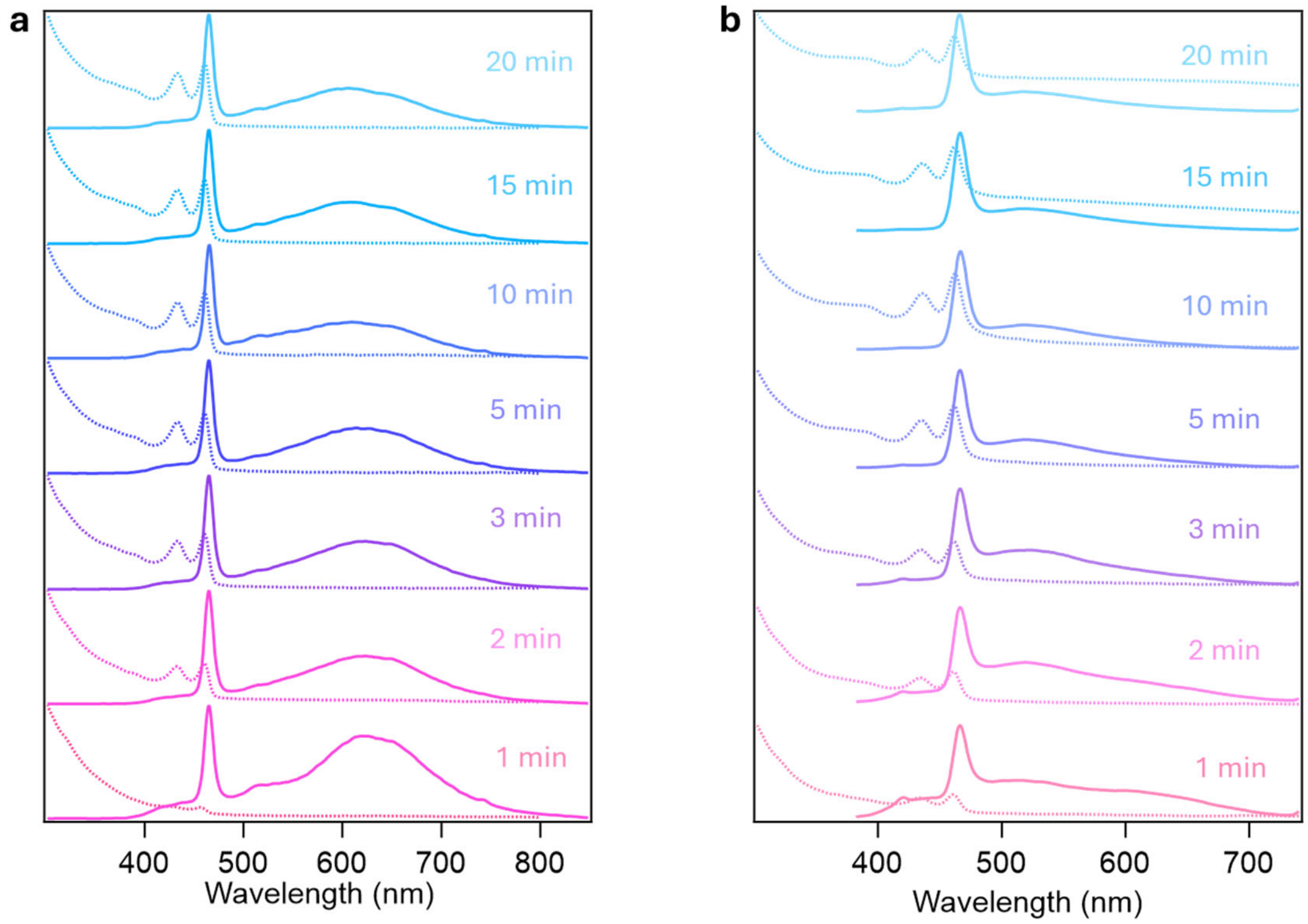

Figure S15. Normalized absorbance (dotted lines) and PL (solid lines) of undoped 3 ML CdSe NPLs at (a) 200 ºC and (b) 250 ºC.

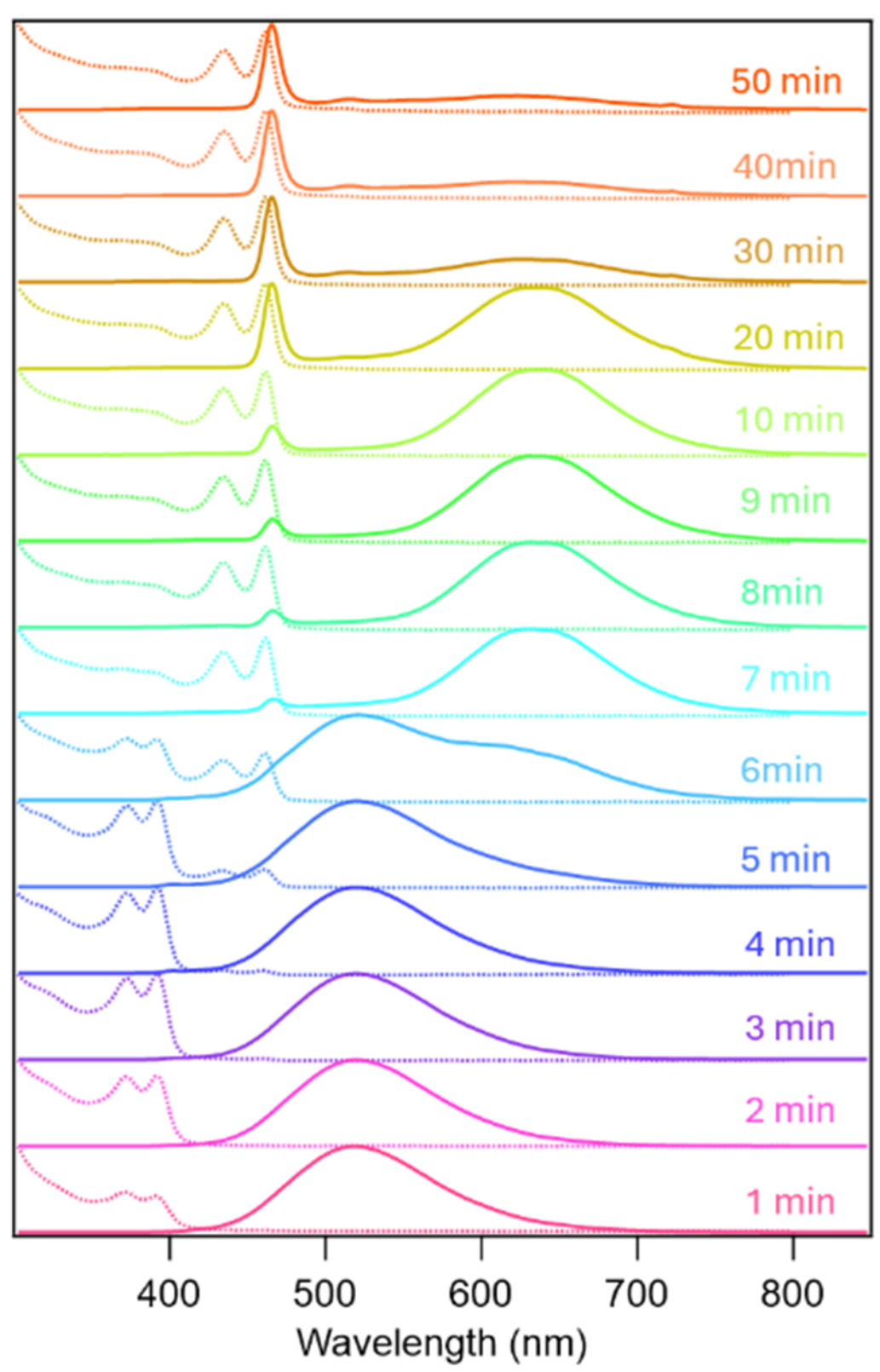

Figure S16. Normalized absorbance (dotted lines) and PL (solid lines) of Cu:CdSe NPLs annealed at 230 ºC for longer reaction time (~1 h).

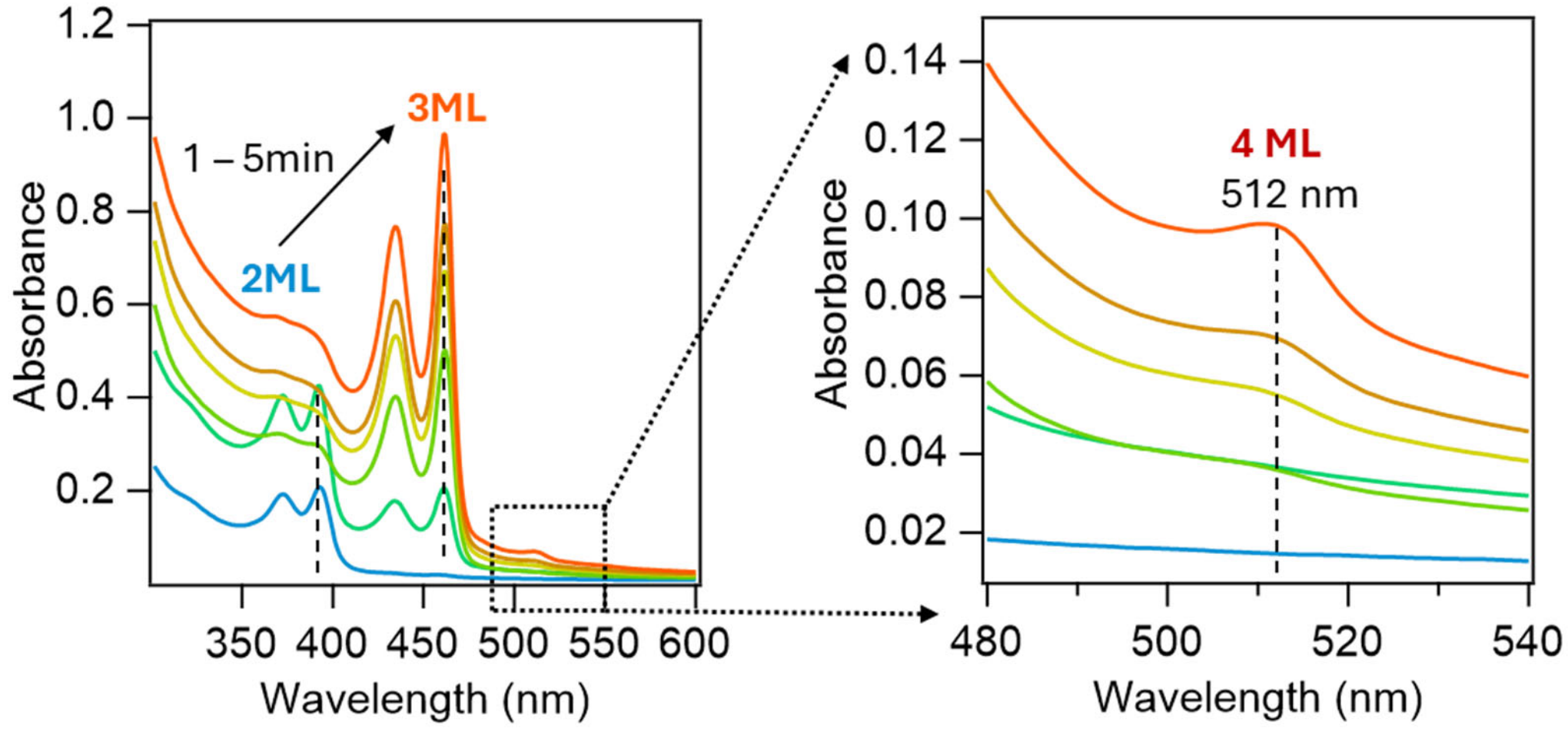

Figure S17. Time-dependent absorbance of Cu:CdSe NPLs at 250 ºC.

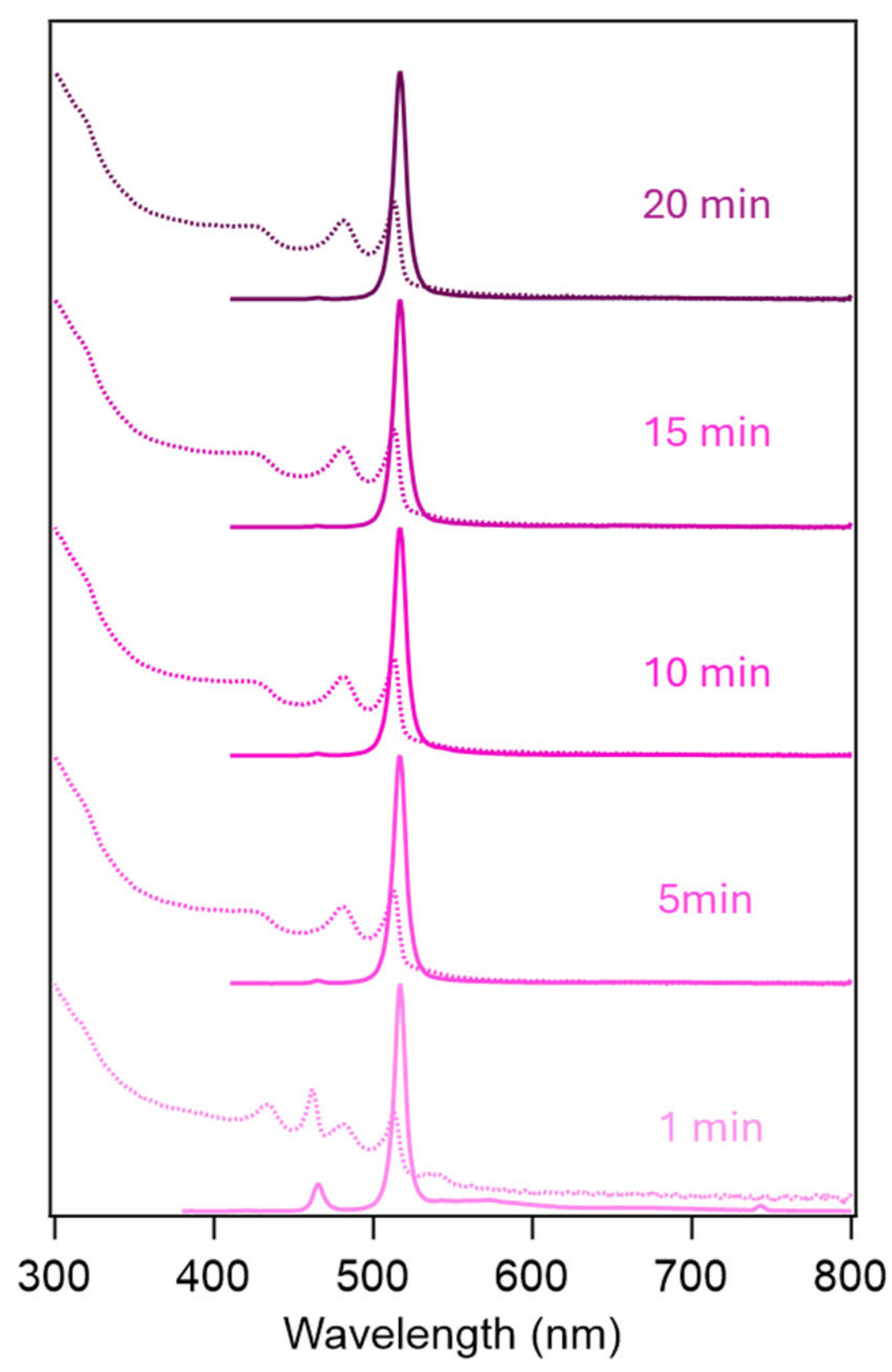

Figure S18. Normalized absorbance (dotted lines) and PL (solid lines) of undoped 4 ML CdSe NPLs.

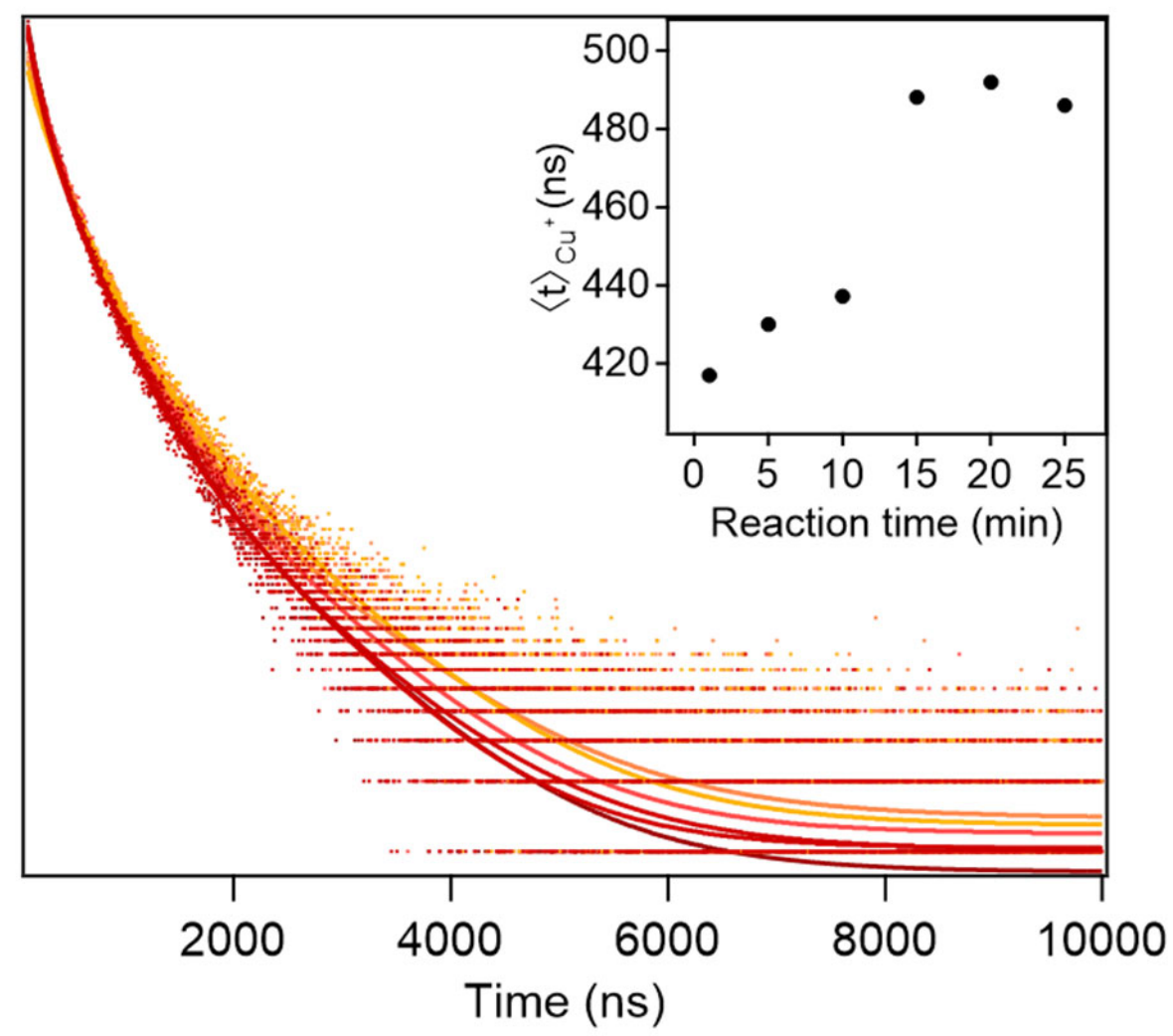

Figure S19. PL lifetime decay of Cu^+ PL at 670 nm is increasing with the formation of 4 ML Cu:CdSe NPLs.

Table S3. The lifetime decay parameters of Cu^+ dopant PL for 3 and 4 ML Cu:CdSe NPLs at three growth stages.

Time (min)	τ_1 (s)	%	τ_2 (s)	%	τ_3 (s)	%
**1**	1.000E-7	**7.8**	3.544E-7	**64.2**	1.000E-6	**28.0**
**5**	1.000E-7	**6.7**	3.714E-7	**59.2**	1.100E-6	**34.1**
**10**	1.000E-7	**6.5**	3.460E-7	**56.4**	9.962E-6	**37.1**
**15**	9.742E-7	**5.0**	3.071E-7	**50.2**	8.805E-6	**44.8**
**20**	1.000E-8	**3.52**	3.364E-7	**24.2**	9.696E-6	**72.2**
**25**	1.000E-8	**2.3**	3.374E-7	**20.6**	9.577E-6	**77.1**

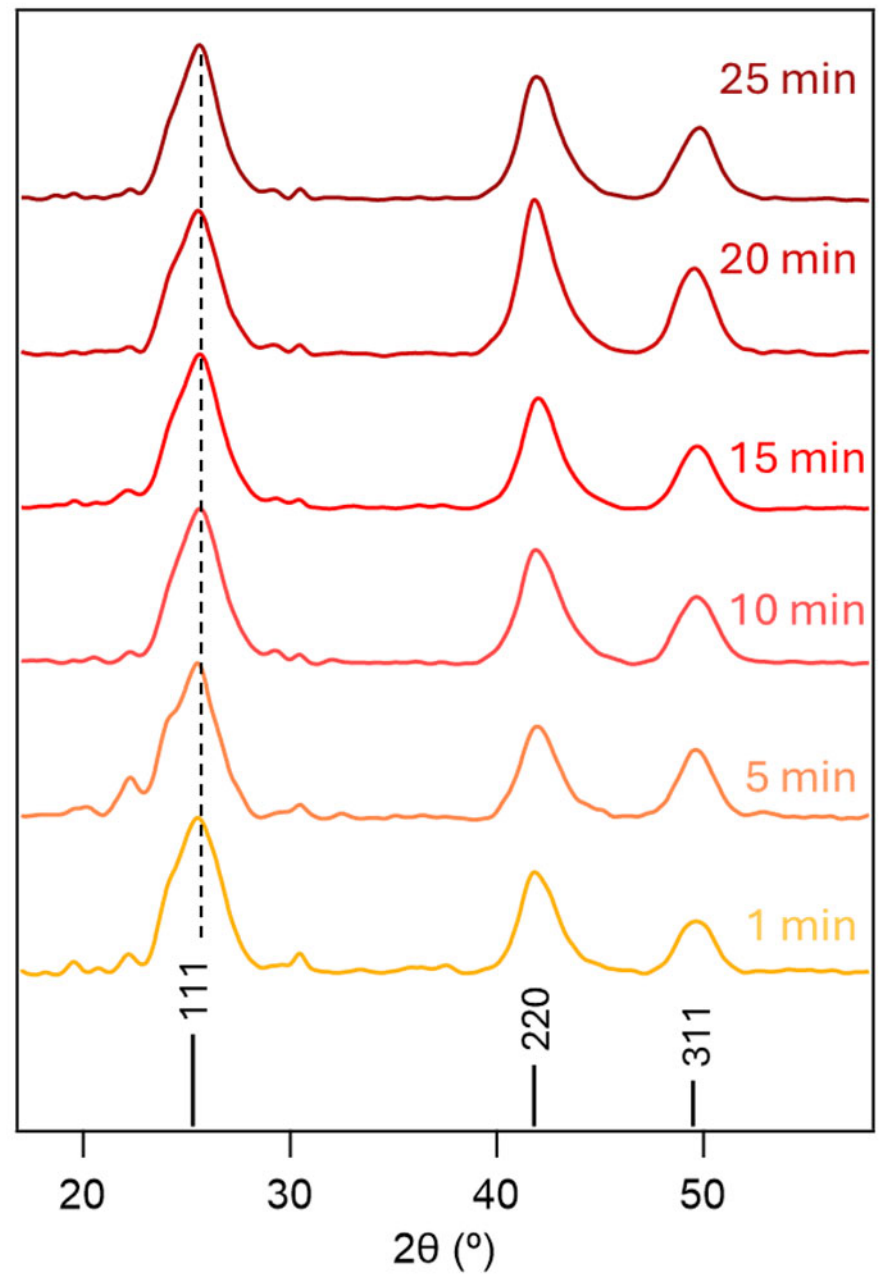

Figure S20. XRD pattern of 4ML Cu:CdSe NPLs as a function of reaction time.

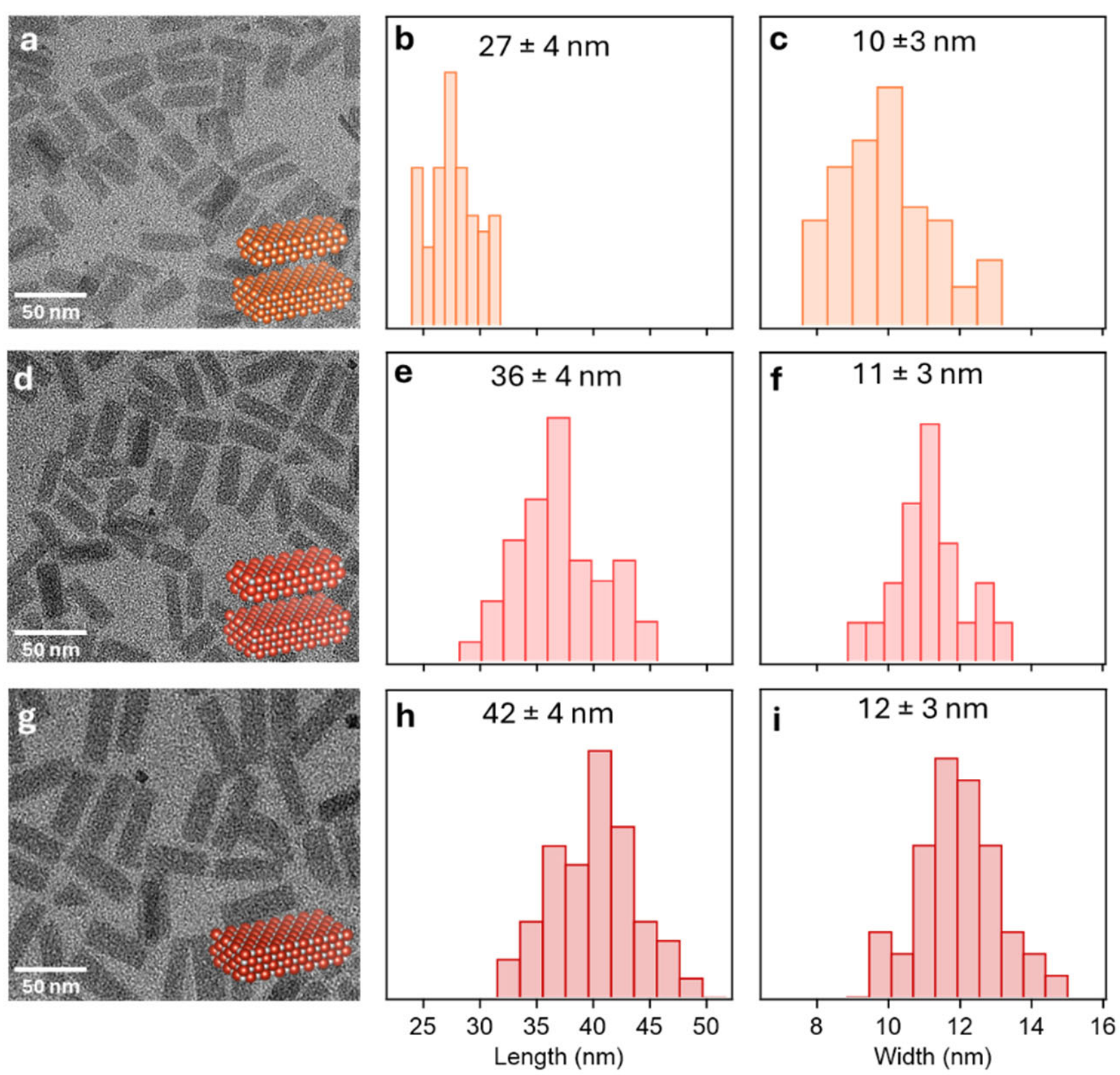

Figure S21. TEM images, size histograms of length, and width of 4 ML Cu:CdSe NPLs at (a-c) 1 min, (d-f) 15 min, and (g-i) 25 min reaction time.

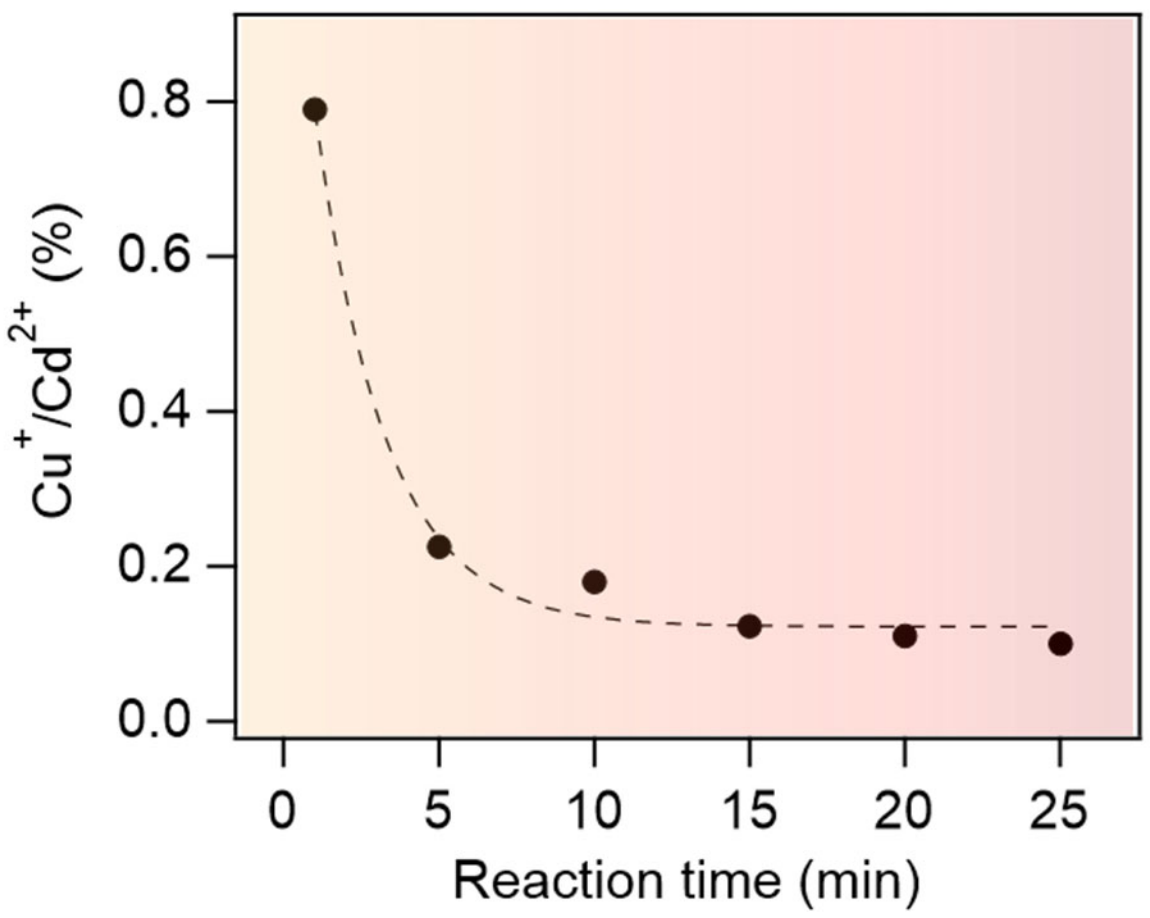

Figure S22. Decreasing Cu^{+}/Cd^{2+} ratio in 4 ML Cu:CdSe NPLs with an increase in reaction time from ICP-OES.

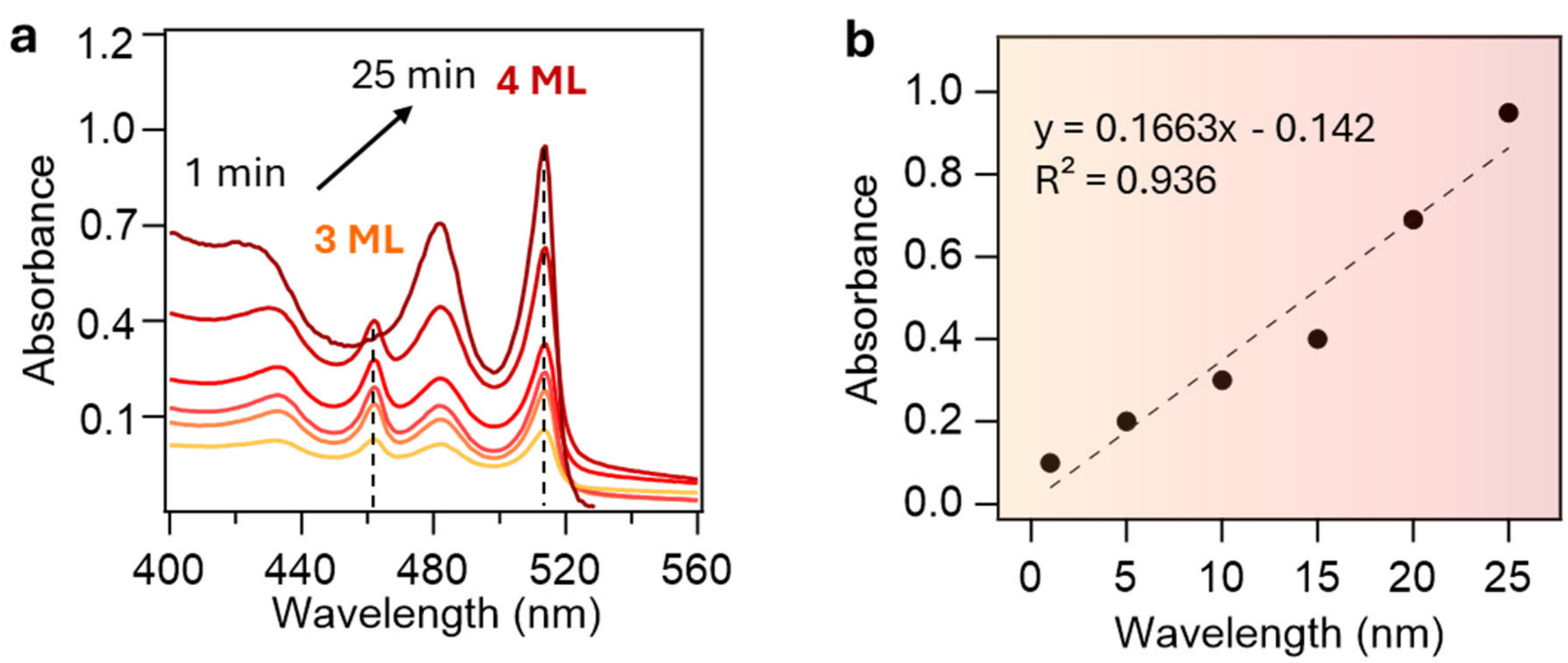

Figure S23. (a) Time-dependent absorbance and (b) linear relationship of 4 ML Cu:CdSe NPLs.

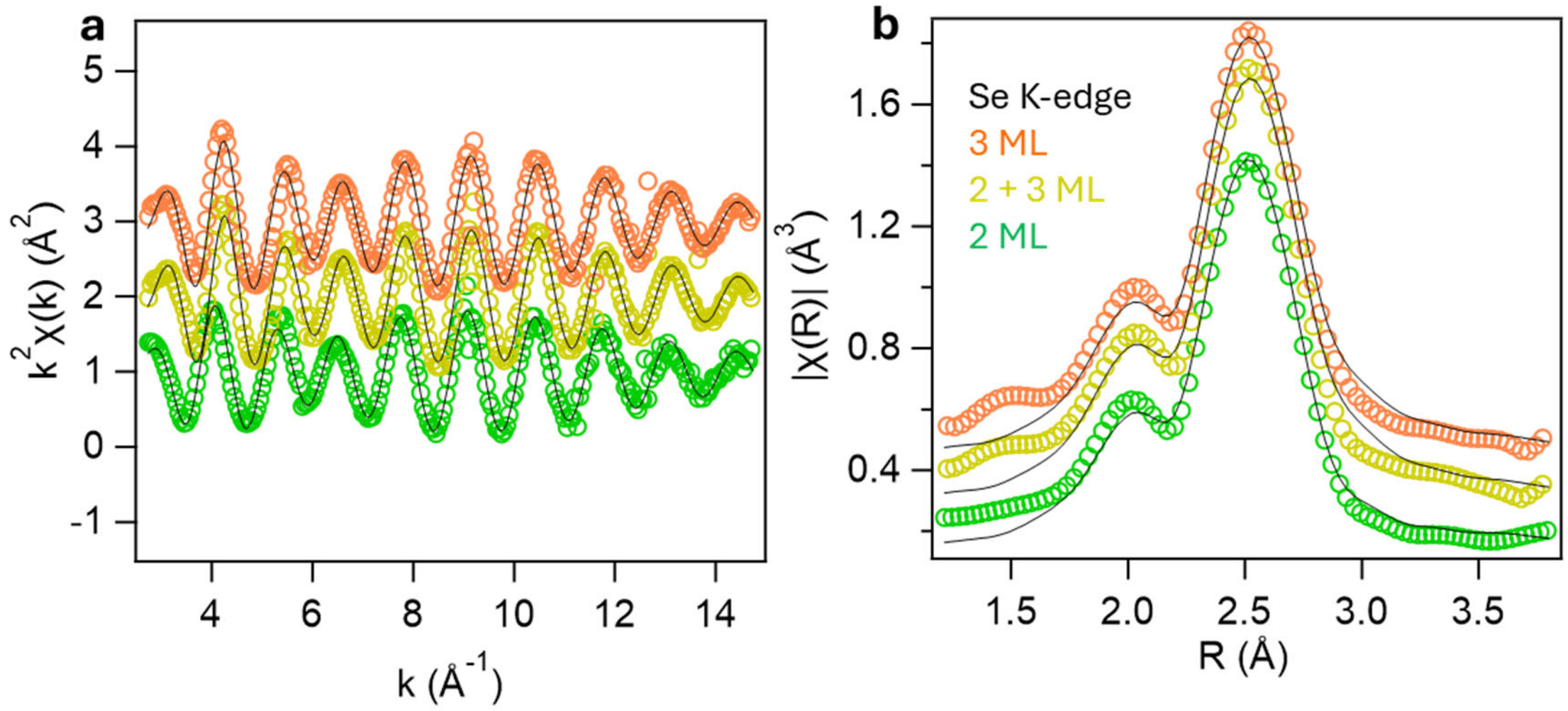

Figure S24. Se K-edge (a) k^2-weighted EXAFS, $k^2c(k)$ and (b) magnitude of the Fourier-transformed EXAFS, $|c(R)|$, of 2 ML (Stage I, green), 2+3 ML (Stage II, yellow), and 3 ML (Stage III, orange) Cu:CdSe NPLs.

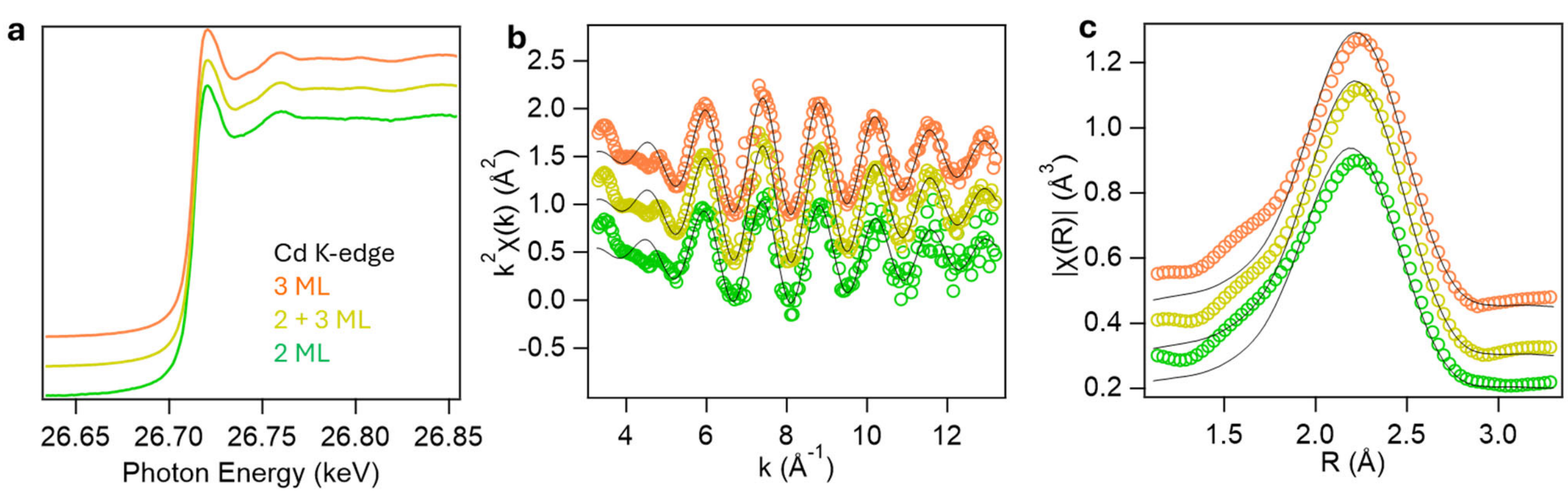

Figure S25. Cd K-edge (a) XANES, (b) k^2-weighted EXAFS, $k^2\chi(k)$, and (c) magnitude of the Fourier-transformed EXAFS, $|\chi(R)|$, of 2 ML (Stage I, green), 2+3 ML (Stage II, yellow), and 3 ML (Stage III, orange) Cu:CdSe NPLs.

Table S4. Summary of Cd and Se K-edge XAFS fits including coordination number (N), bond length (R), mean-squared relative displacements (σ^2), and energy shifts (ΔE_0).

A: Cd K-edge

NPLs	N	R (Å)	ΔE_0 (eV)	σ^2 (Å^2)
Undoped CdSe	4*	2.611±0.019	-0.3±0.020	0.0068±0.012
2 ML Cu:CdSe	3.41±0.070	2.598±0.032	-3.1±0.026	0.0068±0.015
2 +3 ML Cu:CdSe	3.83±0.061	2.610±0.021	-0.5±0.020	0.0067±0.012
3 ML Cu:CdSe	3.83±0.061	2.612±0.019	-0.2±0.020	0.0067±0.012

B: Se K-edge

NPLs	N	R (Å)	ΔE_0 (eV)	σ^2 (Å^2)
Undoped CdSe	4*	2.615±0.016	8.9±0.08	0.00556±0.073
2 ML Cu:CdSe	3.31±0.030	2.612±0.019	3.4±0.07	0.00530±0.063
2 +3 ML Cu:CdSe	3.75±0.037	2.614±0.017	9.5±0.08	0.00562±0.072
3 ML Cu:CdSe	3.77±0.037	2.615±0.016	9.0±0.08	0.00575±0.072

* For the undoped CdSe NPLs, N was fixed at 4 to determine S_0^2 (0.9426 for Cd; 0.9850 for Se). These S_0^2 values were then fixed for all other fits, allowing N to vary.

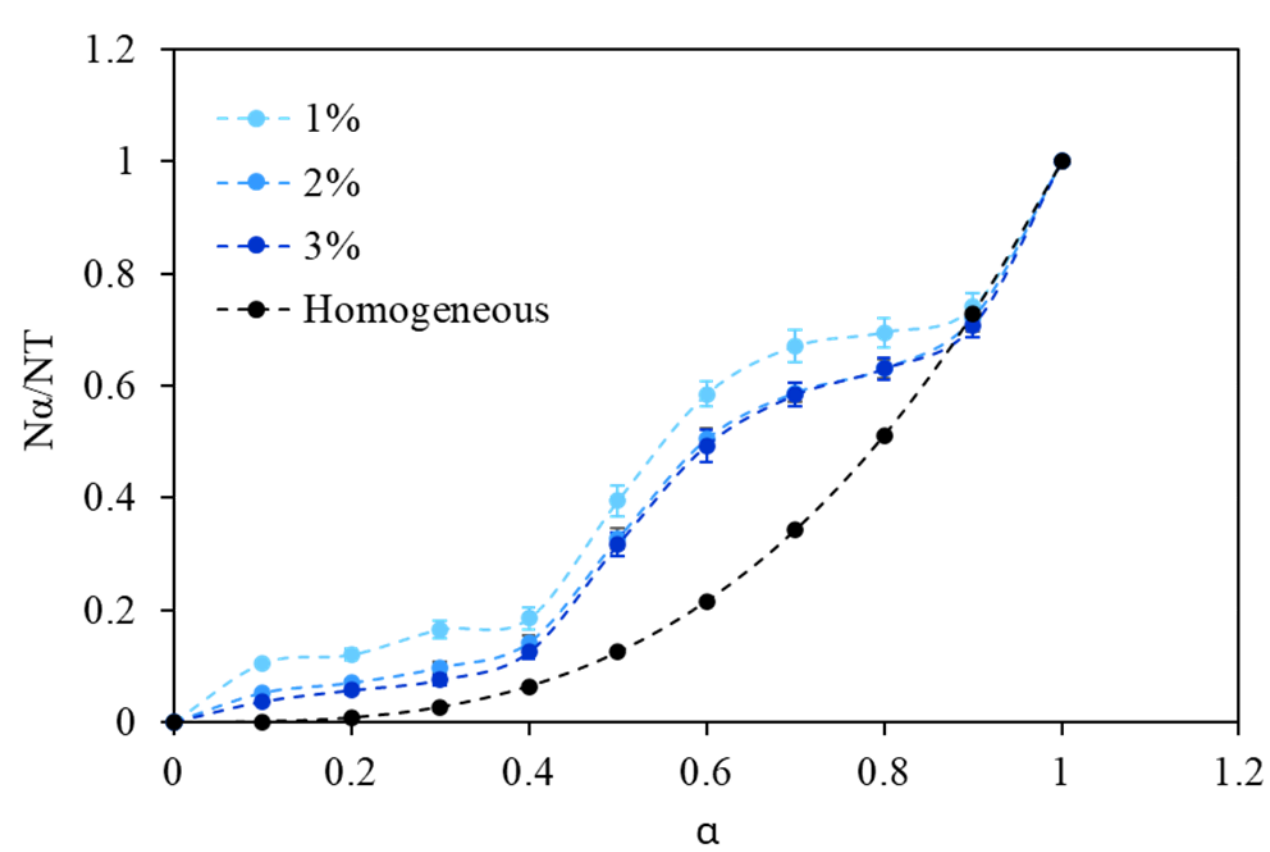

Figure S26. The ratio of number of Cu^+ inside the inner cube and total number of Cu^+ in the NPLs ($N\alpha/NT$) as a function of scale factor, α.

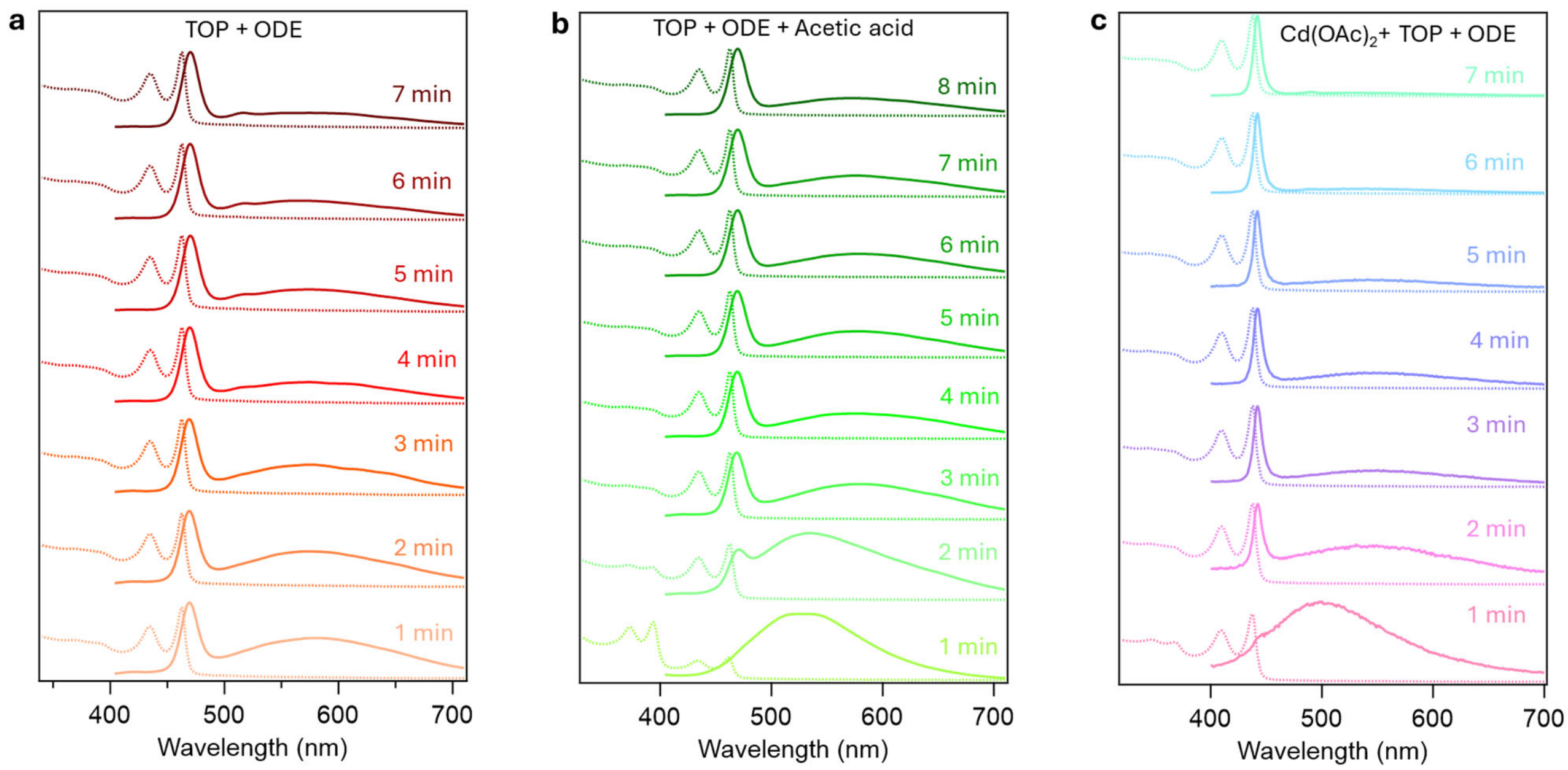

Figure S27. Normalized absorbance (dotted lines) and emission (solid lines) of ligand-dependent synthesis of CdSe NPLs using (a) TOP and ODE, (b) TOP, ODE, and acetic acid, and (c) $Cd(OAc)_2$, TOP, and ODE.

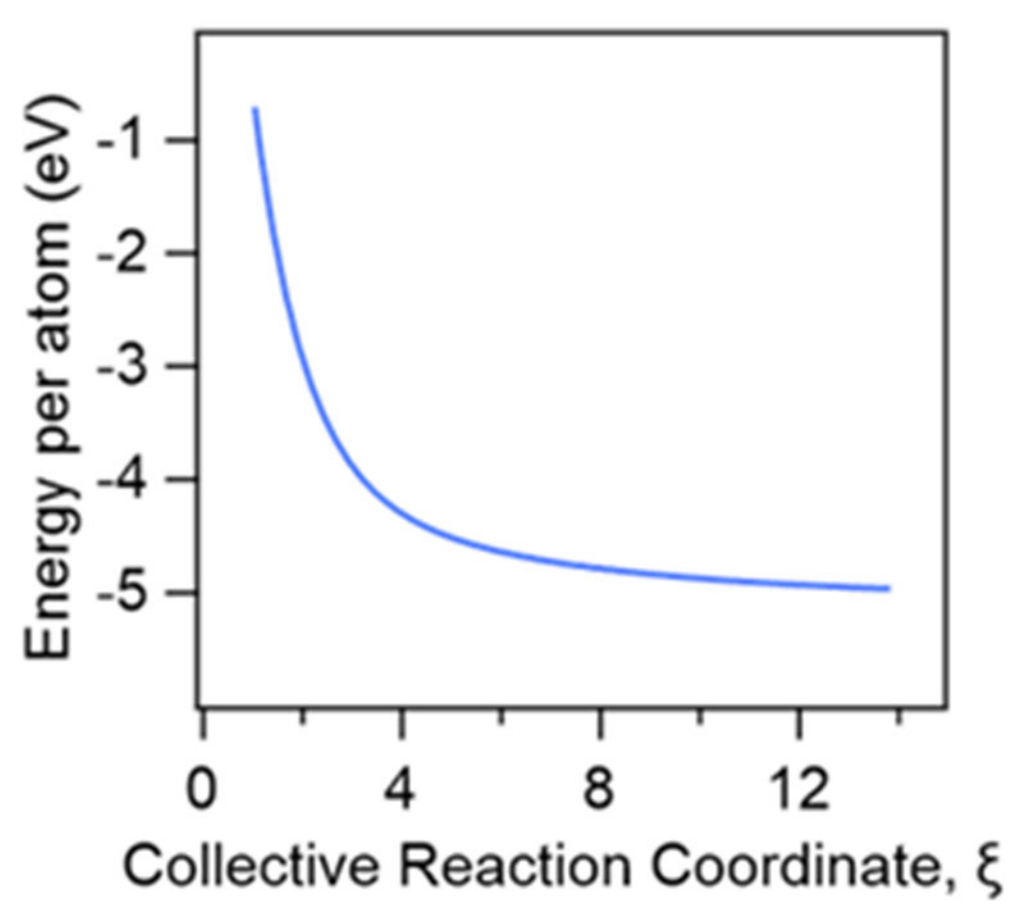

Figure S28. Potential energy per atom as a function of the collective reaction coordinate (ξ) for the undoped CdSe system. The curve reflects the energy-minimizing pathway followed during nanoplatelet growth and is truncated to the first 200 accepted growth steps.

REFERENCES

(1) Mitsuta Y, Yamanaka S, Kawakami T, Okumura M. Automated Search of Minimum Free-Energy Path by Umbrella Integration. *Journal of Computational Chemistry* **2018**, 39(23),1913-21.

(2) Rondina GG, Da Silva JLF. Revised Basin-Hopping Monte Carlo Algorithm for Structure Optimization of Clusters and Nanoparticles. *Journal of Chemical Information and Modeling* **2013**, 53(9), 2282-98.

(3) E W, Ren W, Vanden-Eijnden E. String Method for the Study of Rare events. *Physical Review B* **2002**, 66(5), 052301.

(4) Suzuki Y. Adiabatic Hyperspherical Approach to Large-Scale Nuclear Dynamics. *Progress of Theoretical and Experimental Physics* **2015**, 2015(4).

(5) Namsani S, Gahtori B, Auluck S, Singh JK. An Interaction Potential to Study the Thermal Structure Evolution of a Thermoelectric Material: β-Cu_2Se. *Journal of Computational Chemistry* **2017**, 38(25), 2161-70.

(6) Aquilanti V, Beddoni A, Cavalli S, Lombardi A, Littlejohn R. Collective Hyperspherical Coordinates for Polyatomic Molecules and Clusters. *Molecular Physics* **2000**, 98(21), 1763-70.

(7) Rabani E. An interatomic pair potential for cadmium selenide. *The Journal of Chemical Physics* **2002**, 116(1), 258-62.

(8) Swart JCW, van Helden P, van Steen E. Surface Energy Estimation of Catalytically Relevant fcc Transition Metals Using DFT Calculations on Nanorods. *The Journal of Physical Chemistry C* **2007**, 111(13), 4998-5005.
